# Pesticide degradation capacity of a novel strain belonging to *Serratia sarumanii* with its genomic profile

**DOI:** 10.1007/s10532-025-10144-2

**Published:** 2025-06-01

**Authors:** Gülperi Alatassi, Ömür Baysal, Ragıp Soner Silme, Gülçin Pınar Örnek, Hakan Örnek, Ahmet Can

**Affiliations:** 1 Molecular Microbiology Unit, Department of Molecular Biology and Genetics, Faculty of Science, Muğla Sıtkı Koçman University, Kötekli, Muğla, Turkey; 2 Molecular Plant and Microbial Biosciences Research Unit (MPMB-RU), University of Worcester, Henwick Grove, Worcester, WR2 6AJ UK; 3 Center for Research and Practice in Biotechnology and Genetic Engineering, Istanbul University, Fatih, 34134 Istanbul, Turkey; 4 Republic Turkey Ministry of Agriculture and Forestry, Directorate of Izmir Food Control Laboratory, Izmir, Turkey; 5 Republic Turkey Ministry of Agriculture and Forestry, Directorate of Plant Protection, Reseach Institute Bornova-Izmir, Izmir, Turkey

**Keywords:** Bioremediation, LC–MS/MS, Microbial degradation, *Serratia sarumanii* GBS19, Whole-genome sequence analysis

## Abstract

The extensive use of pesticides in agricultural practices, coupled with the potential for microbial biodegradation of these chemicals, plays a critical role in environmental sustainability. This study aimed to identify microorganisms capable of degrading the most commonly used pesticides in agricultural fields within our region. In vitro screening revealed a microorganism with a broad pesticide degradation spectrum, and whole-genome sequencing further indicated the presence of genomic regions associated with pesticide degradation, a finding that was validated by LC–MS/MS analysis. Detailed genomic analysis, including ribosomal multi-locus sequence typing (rMLST), identified the microorganism as *Serratia sarumanii*. Our results also demonstrated that the introduction of this strain into the environment not only promoted the degradation of specific pesticides but also enhanced the efficacy of certain other pesticides at low concentrations through a synergistic interaction. To further substantiate the biodegradation capabilities of the strain, LC–MS/MS chromatographic analysis of 25 pesticide-active chemicals confirmed that *Serratia sarumanii* effectively biodegrades several pesticide active ingredients, including fludioxonil, fenhexamid, pyrimethanil, and spirodiclofen. These findings underscore the biodegradative potential of *Serratia sarumanii* and its promising application in the bioremediation of pesticide-contaminated soils.

**Graphical abstract:**

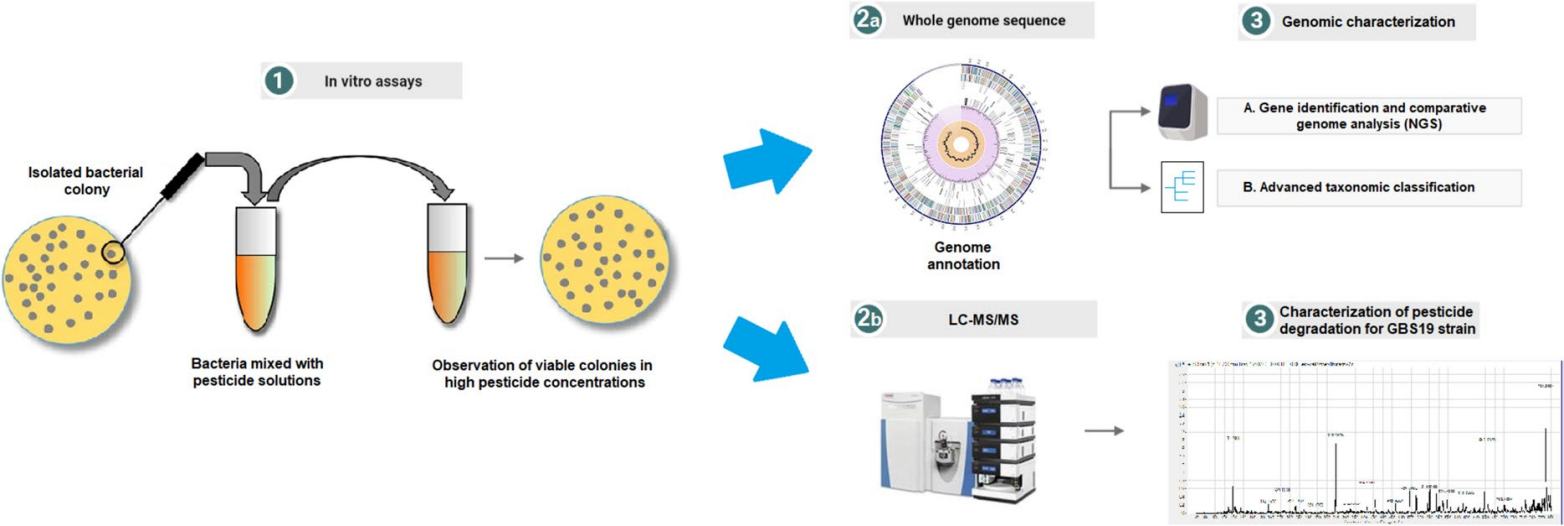

**Supplementary Information:**

The online version contains supplementary material available at 10.1007/s10532-025-10144-2.

## Introduction

The rapid growth of global industrialization and intensive agricultural practices driven by an expanding human population has led to significant pollution of ecosystems, including soil systems (Alori and Fawole 2017). Pollutants present in soil exhibit immunotoxic, carcinogenic, and mutagenic properties, thereby altering the soil’s physical, chemical, and microbiological characteristics (Sales da Silva et al. 2020). Chemical pesticides are extensively used in various sectors of crop production to mitigate pest infestations, maintain crop yields and product quality. A pesticide is any substance or mixture of substances designed to prevent, destroy, repel, or mitigate pests, including insects, mites, nematodes, weeds, and rodents. This category includes insecticides, herbicides, fungicides, and other agents used for pest control (Damalas and Eleftherohorinos 2011; EPA 2012). The application of pesticides has contributed positively by enhancing crop and food productivity, and reducing the incidence of vector-borne diseases (Agrawal et al. 2010). Chemical pesticides can be classified in various ways, with the most common approach being based on their chemical composition. This classification method enables a systematic and scientific grouping of pesticides, facilitating the correlation between their structure, activity, toxicity, and degradation mechanisms, among other characteristics.

Worldwide, the annual pesticide consumption has stood at 3.69 million metric tons (FAO 2024). Among these pesticides, insecticide use in the USA increased tenfold from 1945 to 2000 as reported by Sharma et al. (2019). However, a substantial proportion of these pesticides often fail to reach their intended targets due to degradation, volatilization, and leaching, resulting in significant ecological impacts (Chevillard et al. 2012). In typical agricultural practices, various groups of pesticides are frequently applied either simultaneously or consecutively, leading to interactions among them. Moreover, a population residing in a contaminated site may experience selective pressure from the contamination, potentially resulting in higher resistance levels than a similar population inhabiting an uncontaminated site (Klerks et al. 2011).

Utilizing microorganisms’ capacity to eliminate pollutants in pesticide-contaminated environments has emerged as a potential solution. Bioremediation technology removes contaminants using microbial metabolic activities, which is an affordable, adaptable, and ecologically acceptable technique (Finley et al. 2010). Degradative enzymes that catalyze diverse reactions to transform pesticides into simpler molecules such as CO_2_, water, oxides, or mineral salts are found in microbial species, particularly bacteria (Doolotkeldieva et al. 2018) and fungi (Erguven 2018). These degraded compounds can then be used as carbon, mineral, and energy sources (Senko et al. 2017). Many studies have focused on the metabolic and genetic bases of microbial pesticide-breakdown. In these concepts, pesticide biodegradation-related genes were identified, described, and investigated (Schroll et al. 2004; Aldas-Vargas et al. 2022).

Various types of microorganisms, including bacteria, fungi, and algae, possess the capacity to degrade pesticides. These organisms facilitate the bioremediation of pesticides through diverse metabolic pathways, with enzymatic degradation playing a major role in the chemical transformation of these compounds (Fareed et al. 2017; Yang et al. 2017; Cabrera-Orozco et al. 2017; Herrera-Gallardo et al. 2021; Zhu et al. 2021).

As concerned with our study, the genus *Serratia* is classified within the family *Enterobacteriaceae* of the *Gammaproteobacteria*. *Serratia* spp. are ubiquitous microorganisms found in a wide range of environments, including water, soil, plants, and animals. Some *Serratia* spp. are classified as opportunistic human pathogens (Kurz et al. 2003). Atypical *Serratia* species (Stock et al. 2003), such as *S. ficaria* (Grimont et al. 1979), *S. fonticola* (Gavini et al. 1979), *S. odorifera* (Grimont et al. 1978), *S. plymuthica*, *S. rubidaea* (Ewing et al. 1973), *S. entomophila* (Grimont et al. 1988), and *S. quinivorans* (Ashelford et al. 2002), have been characterized as non-pathogenic (De Vleesschauwer 2008). Unlike many other *Serratia* species, *S. plymuthica* has not concerned with pathogenicity in alternative animal models, such as *C. elegans* (Zachow et al. 2009).

*S. marcescens* is a well-established model organism for investigating biocontrol strategies. In vitro studies demonstrated that the antagonistic bacterium *S. marcescens* B2 suppressed the growth of *Botrytis* spp. and *Rhizoctonia solani* AG-1 IA (Someya et al. 2000; 2001; 2005). *S. marcescens* strain B2 reduced the severity of rice blast disease, resulting in smaller lesion sizes caused by *Pyricularia oryzae* (Someya et al. 2002). Additionally, previous studies identified *Serratia marcescens* strains (MEW06; NCIM 2919; DT-1P) that are able to degraded pesticide and petroleum derivatives as carbon sources (Bidlan and Manonamani 2002; Grewal et al. 2016; Wang et al. 2018).

The aim of this study is to determine the presence of microorganisms that degrade pesticides in soil samples collected from production areas. The samples were collected from production fields to reveal their bioremediation potential. In addition, it was investigated in which pesticide groups this microorganism could be used more effectively. For this purpose, whole-genome sequence analyses of the detected bacteria were performed, and the genes responsible for pesticide degradation were determined. Moreover, advanced chromatographic analyses were performed to investigate the potential for the degradation of chemical compounds belonging to different pesticide groups by the selected bacterial strain.

## Experimental procedures

### Collecting soil samples for bacterial isolation

Soil samples from various tomato greenhouses (located at 36° 50′ 42.47″ N, 28° 44′ 24.55″ E) to be analyzed for pesticide bioremediation were collected from Ortaca district of Muğla province in Turkey. A randomly selected 5 g of soil sample was diluted with 15 ml of distilled water. The solution containing 15 ml soil extract was shaken at 90 rpm for 3 h before filtering using Whatmann filter paper (grade 41, 20–25 μm). Then, 5 µl of cymoxanil fungicide (1.3 × 10⁻^2^ ppm) was added into filtered solution to inhibit fungal growth. 1 ml of the filtered solution was separately added to Nutrient Broth (NB) liquid medium and incubated at 110 rpm under 27 °C for 3 days. Then, 1 ml of the medium was streaked onto NB agar medium. The Petri dishes containing various bacterial colonies were purified and were subsequently screened for their ability to degrade various pesticides (the pesticide list is given in Table S1). The selected colony was also kept in pure culture at + 4 °C and preserved as stock culture in -80 °C with glycerol.

Colony formation and visual appearance on culture media were typical colony morphology of the *Serratia* genus; therefore, we carried out some typical biochemical tests to understand the genus of bacteria in our laboratory conditions (Table S5).

### In vitro biodegradation analysis

The concentrations of all pesticides tested in the culture media for the development of bacterial colonies are presented in Supplementary Tables S1 and S2.

*Insecticide:* Abamectin (18 g/l) which is the active compound of commercial pesticide, was used for testing insecticide biodegradation property of bacterial strain. Media were prepared according to the recommended concentrations of the insecticides (2,5 ml/10 l, 5 ml/10 l, and 7,5 ml/10 l). Adjusted insecticide concentrations were mixed with homogenized sterilized water agar. The pesticide was the sole carbon source for the bacterial strain. Inoculated Petri dishes were cultured at 27 °C for 5 days. The appeared colonies were transferred to Petri dishes containing higher insecticide concentrations as given in Table S2.

*Fungicide:* Penconazole (100 g/l) is another active compound tested for biodegradation. The following concentrations (2,5 ml/10 l, 3,5 ml/10 l, and 5 ml/10 l) were used considering recommended doses. Adjusted fungicide concentrations were mixed with homogenized sterilized water agar. Inoculated Petri dishes were cultured at 27 °C for 5 days. The appeared colonies were transferred to Petri dishes containing higher fungicide concentrations, which were shown in Table S2.

*Herbicide:* Glyphosate (480 g/L) is also other tested active compound. Adjusted herbicide concentrations were 30 ml/10 l, 60 ml/10 l, and 100 ml/10 l. The appeared colonies were selected as described above.

### Selection of a pesticide-biodegrading bacterial strain

Bacterial strains exposed to varying concentrations of different pesticides demonstrated the ability to survive due to their carbon source preferences, indicating their potential for biodegradation as pesticide concentrations increased in the culture media. The bacterial colonies surviving in the highest concentration of whole tested pesticides were selected for cross-tests (the colonies that are able to survive at the highest concentration of any pesticide were transferred from one to another Petri dishes containg different pesticide groups). Finally, the cross-tests indicated that the bacterial strain degraded the entire types of tested pesticides (Supplementary Table S1 and Table S2). Our further studies involved genomic characterization and LC–MS/MS analysis on this selected bacterial strain.

### Whole-genome sequencing of bacterial strain

The bacterial samples were cultured in liquid NB medium at 110 rpm under 27 °C for 1 day, followed by centrifugation at 5000 g for 10 min. DNA was purified from the pellet sections using the CTAB method (Nishiguchi et al. 2002). The purity of the extracted genomic DNA was assessed using 1% agarose gel. The purified DNA was used to construct a library with blunt triple junctions consisting of fragments of approximately 500 base pairs in length. Subsequently, the library was subjected to sequencing using an Illumina Hi-Seq 2500 sequencing system as employed in Bozkurt et al. (2025).

We performed adapter trimming and quality filtering on the reads using Trim Galore version 0.6.7 (Krueger 2024), which incorporates Cutadapt version 3.5 (Martin 2011). The -q parameter was set to 30, and we used the –paired option. The resulting cleaned read pairs served as input for de novo assembly using SPAdes version 3.15.5 (Bankevich et al. 2012). The resulting scaffolds and contigs were re-ordered against the reference genome of strain AR_0027 (NCBI acc. num; CP028702.1). The final assembly was evaluated using QUAST 4.5. Annotation was performed using the PGAP pipeline and RAST (Rapid annotation using subsystem technology; Brettin et al. 2015). The genomic sequence was also annotated using BLAST and compared with the UNiprot and KEGG protein database (Kanehisa et al. 2016; Tatusova et al. 2016).

The final genome assembly comprised 658 contig with a total length of 10,167,833 bp. The longest segment spanned 282,998 bp, and the N50 value was 35,605 (Supplementary data S1). To identify the predicted genes, the assembled genome was subjected to Quast v. 5.0.2 analysis (Gurevich et al. 2013). Subsequently, PATRIC was used for further analysis (Wattam et al. 2017).

Protein sequences were annotated with Enzyme Commission (EC) numbers (Schomburg et al. 2004), Gene Ontology (GO) terms (Ashburner et al. 2000), and KEGG pathways (Kanehisa et al. 2016) using genome annotation pipelines (e.g., RAST and PROKKA) (Aziz et al. 2008; Seemann 2014). The antibiotic production and bacteriocin genes were identified using the SEED viewer server (Aziz et al. 2012). An in-depth analysis of the antibacterial compounds produced by the bacterial strain was conducted using AntiSMASH (version 6.0) to predict gene clusters involved in the production of secondary metabolites (Blin et al. 2021).

From the resulting contig, the 16S rRNA sequence was extracted and compared with reference genomes available in the databases. A phylogenetic tree was constructed using the neighbor-joining method with related sequences obtained from the BLAST output of NCBI (2007).

Gene profiles of the bacterial samples were created by examining the sequence data in the KEGG database. The entire genome of the bacterium (GSB19 NCBI acc. num; PRJNA784190 https://www.ncbi.nlm.nih.gov/nuccore/JAKDDT000000000.1) was mapped against the reference genome (Reference genome: AR_0027 NCBI acc. num; CP028702.1).

Additionally, the GBS19 gbk file was analysed for the average nucleotide identity (ANI) and DNA-DNA hybridisation (dDDH) using The Type (Strain) Genome Server (TYGS) (Meier-Kolthoff and Göker 2019). Then, the fasta sequence of the whole-genome was submitted to PubMLST (https://pubmlst.org/), and allelic variation analyses were performed. Furthermore, the pathogenic gene regions were investigated using Pathogen IslandViewer 4.0 (Bertelli et al. 2017).

### Degradation pathways of *S. sarumanii*

The strain of *Serratia sarumanii* (GBS19) was identified as the bacteria that metabolized pesticides using the full genome sequence. The strain’s position in the phylogenetic tree, the enzymes it encodes, and the degradation processes linked to whole-genome sequencing analysis were visualised. Degradation routes and xenobiotic degradation pathways were assessed among the strain’s related pathways and encoded enzymes.

### *S. sarumanii*’s genes and critical nodes in degradation pathways

Based on the enzymes expressed by the strain, the enzymes playing role in catabolic and metabolic processes at crucial points related to pathways involving pesticide breakdown were evaluated. Using the genes of *Serratia marcescens* AR_0027 (acc. num; CP028702.1) as a guide, the NCBI blast software was used to determine whether our bacterial strain encodes these enzymes.

### Prediction of metabolomics profile

The annotated fasta aminoacid sequences of the whole-genome data were submitted to PIFAR-Pred and PGPT-Pred of PLaBAse (https://plabase.cs.uni-tuebingen.de/pb/plabase.php) using Python modules (Ashrafi et al. 2022; Martínez-García et al. 2016; Patz et al. 2021; 2024), and the results were depicted with KronaTools (Ondov et al. 2011).

### LC–MS/MS analysis for the degradation of active substances

Twenty-five pesticide active components were tested using our strain for biodegradation analysis using LC–MS/MS (TSQ Quantiva/ Ultimate 3000 (Pump)/ Ultimate 3000 (Autosempler Optiplex 9020/Sogevac). The tested concentrations prepared with these active components were incubated with 1 ml bacterial suspension adjusted to 1,7 × 10^8^ cfu/ml at OD_600_ and was growth in shaking culture at 110 rpm for 72 h. Biodegradation ratio of these active compounds was measured using LC–MS/MS. Calibration curves were constructed for analysing the degredation of all active substances by our strain using various concentrations of the tested active compounds (Supplementary data S2, Supplementary Table S3, Supplementary Table S4).

A sensitive scale was used to weigh the powder formulations of active compounds, which were then placed in 5 l sterile bottles containing up to half water and stirred with a magnetic stirrer for 5 min. All preparations containing active substances were prepared in the specified quantities. Depending on the application dose, various dilution factors have been established for each active component. Supplementary Table S4 provides information on the active components of the pesticides used in LC–MS/MS analyses and provides details on the agricultural pests and diseases targeted by the active ingredients. Analysis was also performed with LC–MS and GC–MS depending on the active ingredient of the pesticide and the degradation amounts were determined according to the best response value compared to blank samples. Detailed information on GC–MS analysis was given in Supplementary Table S4.

## Results

Bacterial groups in labeled tubes were examined for microbial biodegradation after incubation. The isolates were analyzed for the biodegradation of commercial pesticides such as insecticides, herbicides, and fungicides.

The preliminary phenotypic characterization tests indicated that the bacterial isolate belonged to the *Serratia* genus (Suplementary Table S5, Supplementary Figure S1). To identify the bacteria, we continued our studies with genome-scale analysis.

In vitro tests showed that our strain can degrade various pesticides, because the colonies were viable and visible on culture media even at high concentrations. Afterwards, we carried out LC–MS/MS analysis to observe the degradation capacity considering active compound quantity.

### Whole-genome sequence analysis and identification

**Fig. 1:**
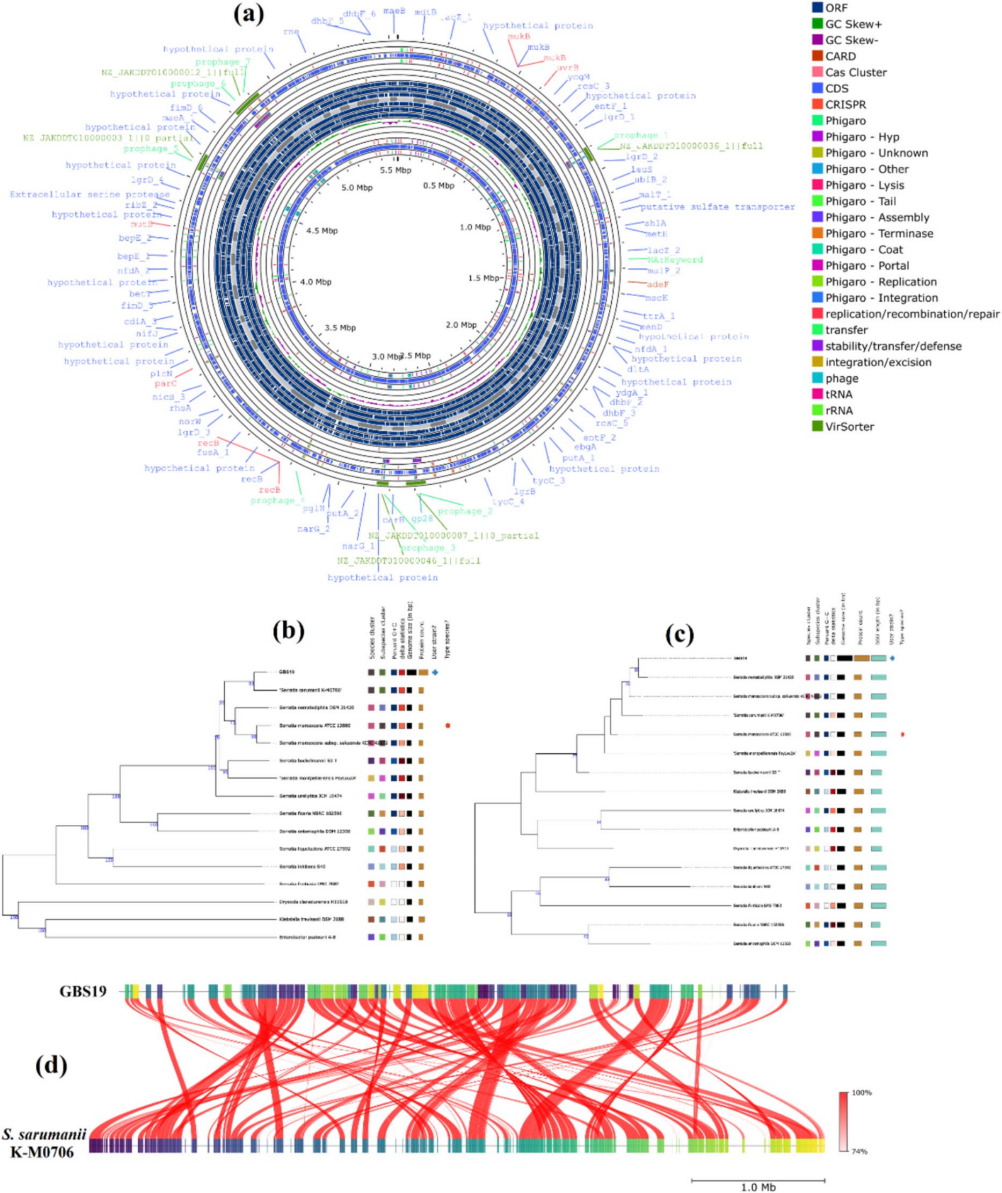
The illustration shows genetical identification depending on whole-genome sequence data of our strain. **a** The illustration shows a circular graphical display of the distribution of the genome annotations: CDS, tRNA genes, rRNA genes, ORF regions, GC skew ± , CARD genes, and phage integration regions in the bacterial genome. **b** The data depicts DNA-DNA hybridisation (dDDH) using the TYGS database. “** + **” denotes our strain, which is genetically similar to *S. sarumanii* K-M0706. **c** The figure shows the 16S rDNA results obtained using the TYGS database. “** + **” denotes our strain which is genetically similar to *S. nematodiphila* DSM 21420. **d** Whole-genome ANI comparison between reference *S. sarumanii* K-M0706 and GBS19

**Fig. 2:**
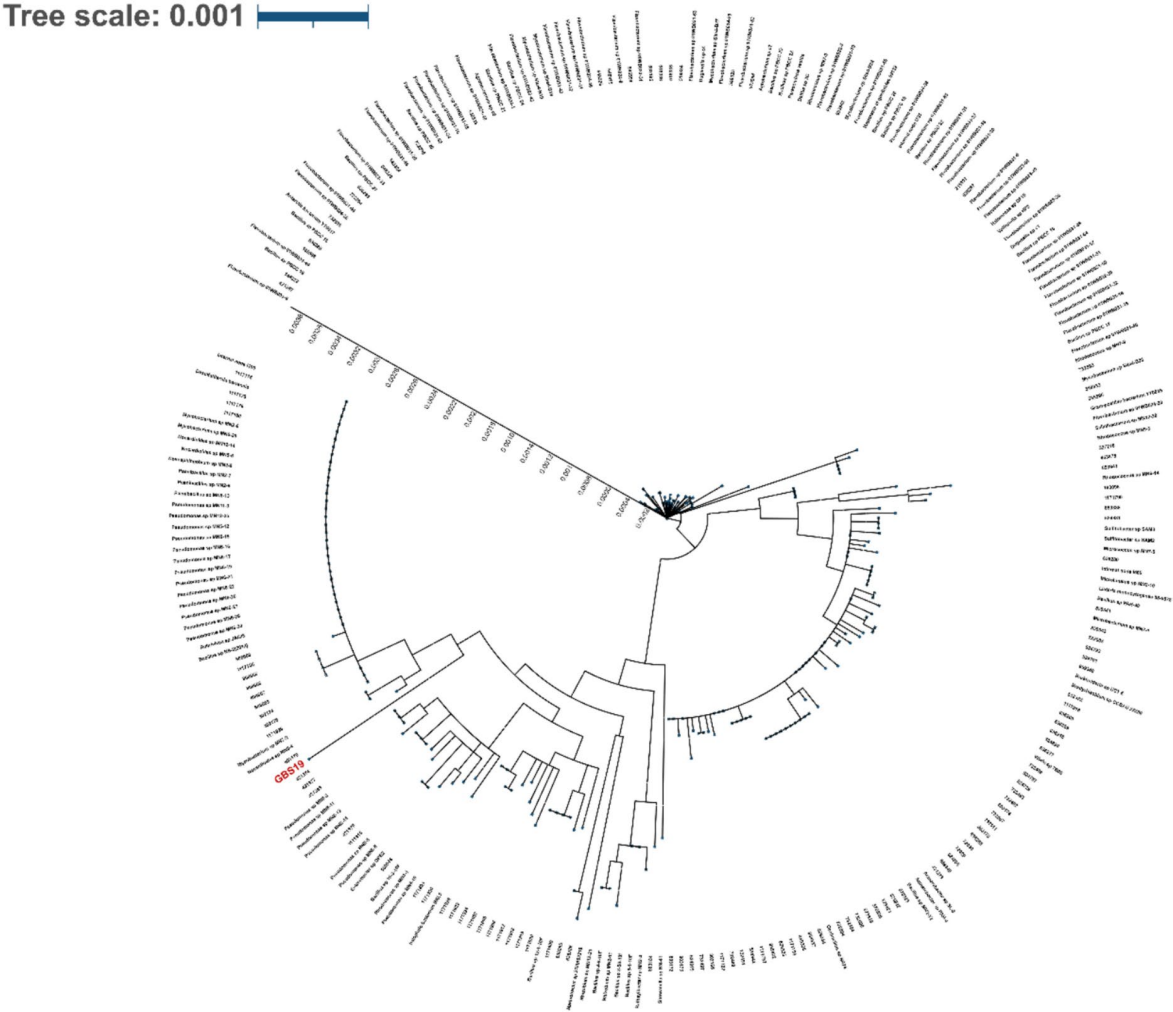
Ribosomal MLST analyses output obtained from PubMLST database and the phylogenetic tree was constructed considering allelic variations of GBS19 (shown in red) using the ITOL tool

The whole-genome sequence and its encoding enzymes with phylogenetic tree analyses have been extensively investigated (Supplementary data S3). We submitted genomic data to NCBI with the accession number PRJNA784190. Genetical identification was illustrated using PROKSEE and whole-genome data, and the distribution of CDS, tRNA genes, rRNA genes, ORF regions, GC skew ± , CARD genes, and phage integration regions in the genome of the bacterial strain has been shown in Fig. 1a (Supplementary data S4). The ANI result was depicted considering reference genome *S. sarumanii* K-M0706. Even 16S rDNA analysis indicated that our strain GBS19 was genetically close to *S. nematodiphila* DSM 21420, *S. marcescens* ATCC 13880 and *S. marcescens* subsp. *sakuensis* KCTC 42172 (Fig. 1b, c). Genomic comparison based on DNA-DNA hybridisation (dDDH) proved that our strain belongs to *S. sarumanii* genus (Supplementary data S5) (Fig. 1b), but it was genetically close to *S. marcescens* subsp. *sakuensis* KCTC 42172, which was confirmed by ANI analysis (Fig. 1d) that some regions are different which could be associated with the evolutionary diversification of the strain. To validate our genomic identification of our strain, we confirmed our results with PubMLST database. In addition, detailed genomic analyses linked to allele sequence definitions for ribosomal MLST have shown that this strain belongs to *Serratia sarumanii* compared to *S. marcescens* and *S. nematodiphila* under the *Serratia* genus, according to rMLST (Fig. 2; Supplementary data S6). Furthermore, 7 housekeeping genes with allelic variations were determined using MLST analysis by PubMLST after submitting genomic data to the server (Jolley et al. 2012; Supplementary data S7).

**Fig. 3:**
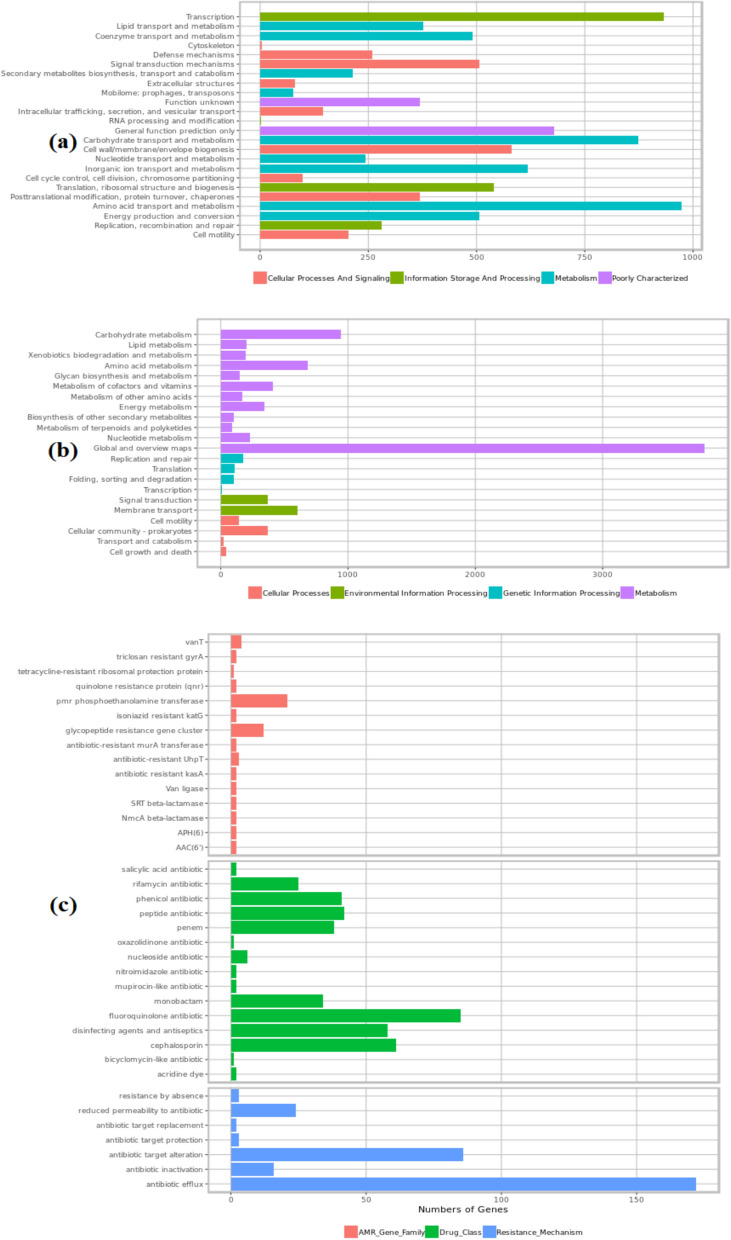
Number of our strain’s genes related to **a** molecular functions and cellular components, **b** biological processes, **c** comprehensive antibiotic resistance database outputs according to genomic data submitted from SEEDviewer 2.0

Our strain’s genes related to molecular functions and cellular components (Fig. 3a), biological processes (Fig. 3b), and comprehensive antibiotic resistance (Fig. 3c) are presented. In accordance with the subsystem feature count of the genes in the genome linked to the metabolism of aromatic compounds, 71 genes were identified in the strain (Supplementary Figure S2). Supplementary Table S6, S7 and S8 show the encoding genes linked to degradation pathways in *S. sarumanii* genome.

Our strain’s molecular function, cellular component, and biological process percentages were determined using the entire genome sequence information (Fig. 3). We searched for links between the enzyme information encoded by our strain GBS19 and mechanisms related to enzymatic pathways. The KEGG database mapping metabolic pathways and enzyme classification were determined according to Kanehisa et al. (2016; Supplementary data S8).

**Fig. 4:**
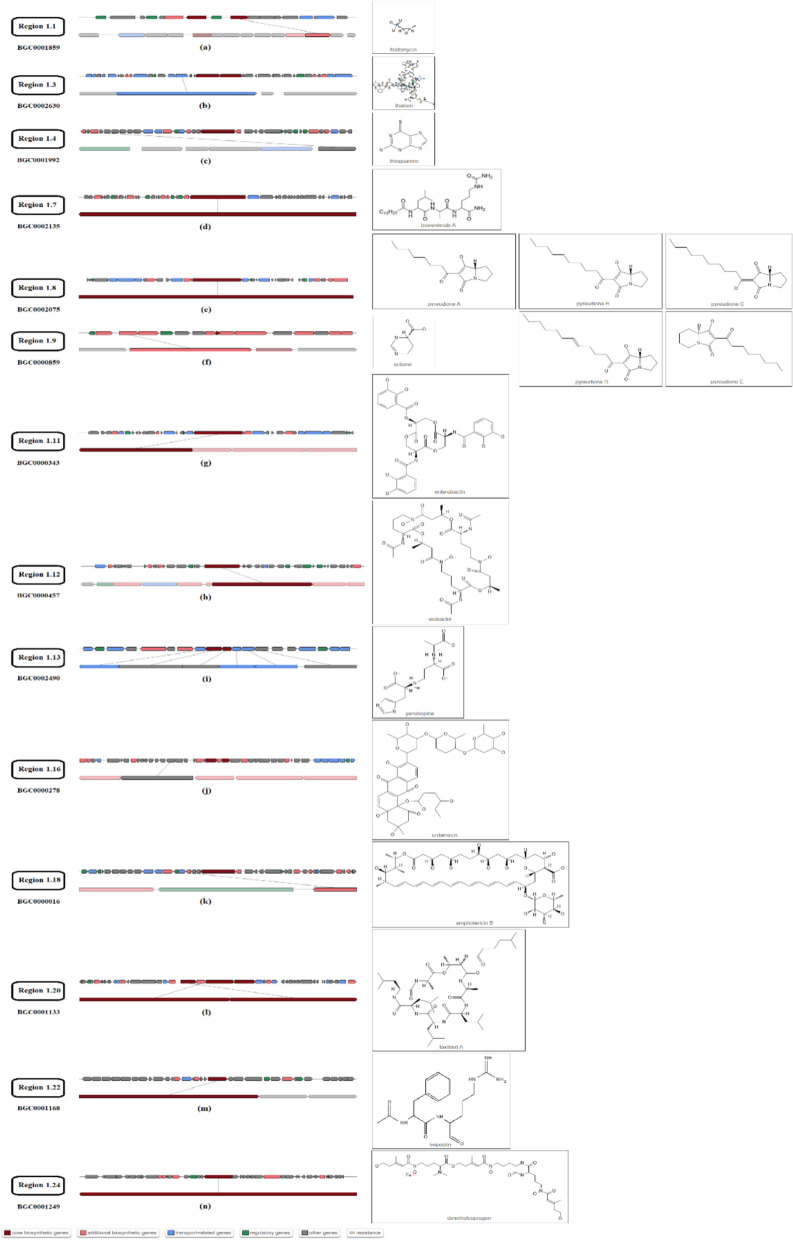
Antimicrobial metabolites produced by our strain were identified using the antiSMASH database according to whole-genome sequence data. Genes encoding proteins responsible for biosynthesis of **a** fosfomycin, **b** thatisin, **c** thioguanine, **d** bovienimide A, **e** pyreudione A, B, C, D, E, **f** ectoine, **g** enterobactin, **h** vicibactin, **i** yersinopine, **j** urdamycin, **k** amphotericin B, **l** taxlllaid A, **m** livipeptin, and **n** dimethylcoprogen. Repetitive metabolites and metabolites below 0.20 MIBiG comparison value were ignored and not considered

The whole-genome sequence of our strain was submitted to the antiSMASH database and compared with the other available gram-negative and gram-positive prokaryotic genomes. The results showed a similarity between the antimicrobial metabolites, as depicted in Fig. 4 (Supplementary data S9). Furthermore, Pathogen IslandViewer 4.0 results revealed that there were no pathogenic regions in our strain (Supplementary Figure S3).

As depicted in supplementary data 1, the genes encoding enzymes were identified and listed. All genes related to the degradation of aromatic compounds showed that the strain can degrade hydrocarbons and various chemicals.

### Predictive metabolomics profiling

**Fig. 5:**
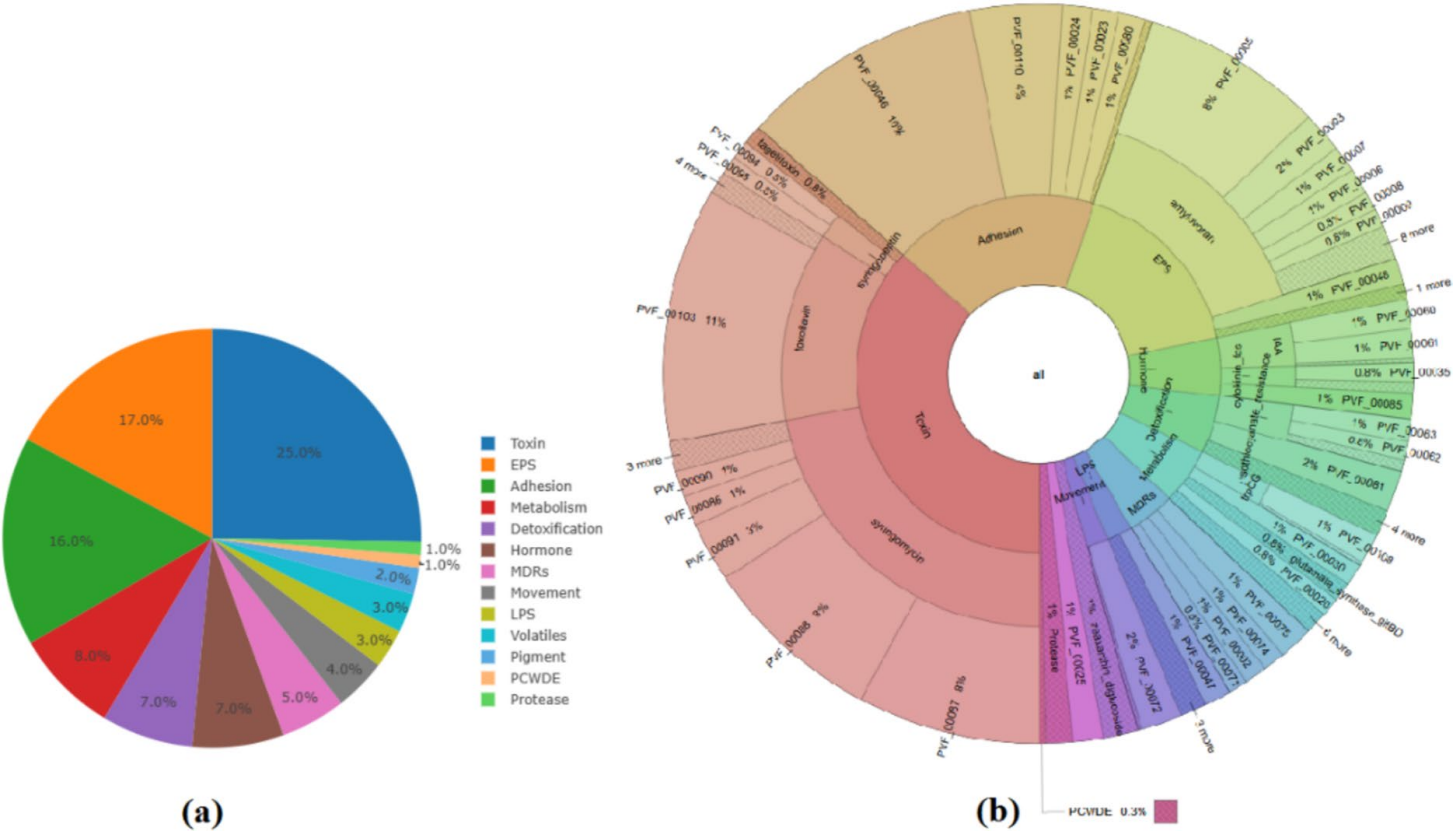
**a** Pie chart and **b** Krona chart of predicted metabolites produced by the bacterial strain

Predictive metabolomics profiling of the strain using the PLaBAse database indicated a variety of disparities in the produced metabolites. These genes mainly encode toxins, exopolysaccharides (EPS), adhesion and e.g. related compounds (Fig. 5a and b).

### LC–MS/MS analysis results for the pesticide-active substance degradation

Among all tested pesticide groups shown in the Supplementary Table S4, the strain was found to significantly degrade one pesticide from Group 2, three from Group 3, one from Group 5, three from Group 6, one from Group 7, and two from Group 8 (Table 1). The highest degradation rates were observed for Pyrimethanil, Fenhexamid, Spirodiclofen, and Fludioxonil (Supplementary Table S4).

**Table 1:** LC–MS/MS analysis of the active ingredients

Groups	Pesticide active ingredients	Standard	GBS19 ( +) 72 h	GBS19 (-) 72 h	Biodegradation ratio %
2. Group	Pyrimethanil	957 ± 0.02	435** ± 0.01	899 ± 0.02	**51,62**
3. Group	Fenhexamid	2829 ± 0.03	1458** ± 0.02	1771 ± 0.01	17,67
	Indoxacarb	131 ± 0.03	111* ± 0.02	217 ± 0.01	**48,85**
	Spirodiclofen	420 ± 0.02	98*** ± 0.03	306 ± 0.03	**67,97**
5. Group	Metrafenon	426 ± 0.01	302* ± 0.03	354 ± 0.01	14,69
6. Group	Azoxystrobin	613 ± 0.01	379* ± 0.03	416 ± 0.01	8,9
	Difenoconazole	324 ± 0.03	319* ± 0.02	411 ± 0.01	22,39
	Fludioxonil	477 ± 0.03	255** ± 0.03	334 ± 0.02	23,65
7. Group	Gamma Cyhalothrin	42 ± 0.02	40* ± 0.03	48 ± 0.01	16,67
8. Group	Boscalid	5503 ± 0.01	4199* ± 0.01	4388 ± 0.03	4,31
	Spirotetramat	322 ± 0.02	199* ± 0.01	287 ± 0.03	30,66

Values of ± denote the standard deviation of 3 replicates

Asterisks indicate the significance of the measurements

Standard; pure active ingredient of the pesticide, GBS19 ( +) 72 h; the solution containing pesticide incubated with bacterial strain for 72 h, GBS19 (-) 72 h; the solution containing pesticide incubated without bacterial strain for 72 h

The bold numbers indicate biodegradation ratio higher than 45%

## Discussion

Worldwide, annual pesticide consumption shows an ascending trend, depending on agricultural production and consumption. Nevertheless, the environmental behavior and ecotoxicological impacts of many pesticides remain poorly understood (Carvalho 2017). Post-application, a considerable number of pesticides penetrate the soil, where processes such as mineral adsorption and microbial degradation act as natural filters, protecting groundwater and surface water from contamination (Keesstra et al. 2012; Sun et al. 2010). The ability of microorganisms to remove pollutants by disintegration through various enzymatic reactions in areas contaminated with pesticides is an alternative and promising method. The methods of microorganisms to remove pollutants are provided via a technology known as bioremediation, which has emerged as an economical and environmentally friendly approach (Finley et al. 2010). The underlying principle of bioremediation is to decompose toxic contaminants through biodegradation and transform them into less toxic or non-toxic elements or compounds (Strong and Burges 2008). Silva et al. (2019) reported that only 17% of 317 agricultural topsoil samples from the European Union were free of pesticide residues. While 25% of the soils were contaminated with individual pesticide residues, 58% contained pesticide mixtures at medium and high concentrations ranging from 0.02 to 0.04 mg kg^−1^ and 0.31 to 0.41 mg kg^−1^, respectively.

Despite their biodegradability under ideal laboratory conditions, numerous organic pollutants persist in soil environments (e.g., 2,4-dichlorophenoxyacetic acid) (Fenner et al. 2013; Nowak et al. 2011). Numerous studies have highlighted the adverse environmental impacts of conventional pesticides. In addition to harming non-target organisms within the ecosystem, these chemical pesticides can also migrate beyond the application site (Kalia and Gosal 2011). Since the side effects of pesticides on human and animal health have shown by different studies (Bassil et al. 2007; Hayes et al. 2010), there is a great interest among researchers and farmers for more environmentally friendly approaches (DG-SANTE 2024).

The population dynamism of microflora may differ at genomic level according to abiotic and biotic stress. In particular, bacteria become resistant to various stresses, including antibiotics and pesticides, because of their evolved genes. This approach changes the genome stability and the survival of bacteria (Davies and Davies 2010). Pesticide-degrading enzymes are encoded by the genes of bacteria present on chromosomes, transposons, and plasmids (Bahig et al. 2008).

The bacterium identified as capable of metabolizing pesticides was classified to be *Serratia sarumanii* based on genomic analyses, including ribosomal multilocus sequence typing (rMLST) and multilocus sequence typing (MLST) using seven housekeeping genes. The identification analysis of the strain revealed that it was closely matched by 81% with *Serratia sarumanii* and 10% with *Serratia nematodiphila* (Supplementary data S6; S7). In our research, we submitted this strain into the database as GBS19 and registered its whole-genome sequence as a novel strain in the NCBI (acc. num: PRJNA784190). Furthermore, in the identification of the strain, there may be situations in which using 16sRNA analysis alone, in addition to phenotypic characterization, may give misleading results, and even MALDI-TOF MS analyses could be insufficient to respond to new taxonomic distinctions at the subspecies level if MALDI-TOF MS library is not expanded well by introducing reference strains of various bacterial species, we have also experienced this situation during our previous studies on this strain. In accordance with this finding, this strain was previously identified as *Serratia marcescens* but rMLST analyses indicated precisely its subspecies level under *Serratia* genus as *Serratia sarumanii.*

Following preliminary phenotypic characterization and initial genomic analyses, the strain was identified as *Serratia nematodiphila*, showing close genetic similarity to *Serratia marcescens* based on 16S rRNA gene sequencing. However, subsequent genome-to-genome comparisons revealed that the strain aligned more closely with *Serratia sarumanii*, leading to taxonomic ambiguity. To resolve this uncertainty, further analyses were conducted using ribosomal multilocus sequence typing (rMLST) and multilocus sequence typing (MLST) databases. These additional methods confirmed that the strain belonged to *Serratia sarumanii*, consistent with the findings from genome-to-genome hybridization analysis.

Our data show that when identifying a bacterium at the genomic level, examining genetic allelic variations and characterizing them via housekeeping genes yield more reliable results. We found it appropriate to highlight this output as it will help in further studies on the identification of various bacterial strains.

As sequence data in genomic databases continue to accumulate, it becomes increasingly likely that certain populations currently classified within *Serratia marcescens* may be reclassified. These populations may exhibit closer genetic affiliation with other subspecies, particularly those aligned with *Serratia sarumanii*, and could therefore be reassigned as part of this or a related taxonomic group based on emerging phylogenetic evidence (Klages et al. 2024). In fact, since our strain was isolated from a soil where agricultural production was carried out, it showed a close relationship to *S. nematodiphila*, the closest to it, based on genetic results, rather than being a human pathogen. Therefore, the probability of this strain interacting with humans or exhibiting pathogenic potential appears to be low. We also confirmed the nonpathogenic properties of our strain using Pathogen IslandViewer 4.0 (Bertelli et al. 2017).

AntiSMASH analysis also revealed that our strain produces several antimicrobial compounds that inhibit various pathogenic microorganisms and enhance soil antipathogenic potential (Yegen and Heitefuß 1970)*.* These protein-encoding genes can be used in recombinant DNA technology for large-scale production and formulation.

Our strain possesses the genetic foundation necessary to activate appropriate metabolic pathways by encoding enzymes. The genes found in the *S. sarumanii* strain are linked to degradation processes. We found that the *S. sarumanii* strain expresses various genes related to biodegradation and metabolic processes in multiple pathways. Predictive metabolomics analysis revealed that the strain exhibits a variety of PVF-mediated signalling properties, which can be linked to cellular physiology and increasing of its virulence. Additionally, degradation of pesticides by bacteria could lead to synthesizing of various metabolites related to plant defence and hormones, from which the plant uses these secreted compounds for its life-cycle. The genomic data highlighted the presence of genes encoding toxins, EPS, adhesion factors, and other compounds that are involved in PVF-mediated signalling pathways. These pathways play a significant role in bacterial virulence, colonization, and competition within soil microflora (Kretsch et al. 2021).

Our findings indicated that our strain can metabolize some of the active components of tested pesticides to lower quantities and use them as a carbon source. Interestingly, LC–MS/MS analysis revealed that the active compound concentrations of certain pesticides increased following their interaction with the bacterial strain. These results should be expanded and confirmed with new studies. However, the results indicate that the combined application of GBS19 with pesticide concentrations below the recommended levels could produce a synergistic effect, which will result in comparable efficacy with full recommended dose. In the pesticide residue degradation analyses, the initial concentration of 11 out of 25 active substances degraded in the solutions in which the bacteria and pesticide were tested and incubated for 72 h. As well known, when plants are exposed to pesticides, the chemicals start to degrade from the initial day; however, when they are prepared at the application dose and preserved in a closed tube until analyses, the degradation rates have shown differentiation compared to the introduced doses on plants. Pesticide degradation depends on many factors, such as the pH of solution and the water solubility coefficient of the active substance, as well as other plant-related factors. On account of the pesticide *K*_*ow*_ values​​ = indicating relationship between lipophilicity and hydrophilicity = directly affect the recovery values ​​in pesticide analyses, the waiting period (72 h) for some pesticides causes different results depending on the initial concentration. Since the tested bacteria use the main active pesticide molecule as a carbon source, they produce metabolites that change the pH and dissolution coefficient of the solution (water + pesticide). As a result of degradation in the amount of pesticide, it is anticipated that this situation will cause some pesticides to give a higher recovery and response rate, which was detected in the LC–MS/MS analysis (Supplementary Table S4). We determined these changes in LC–MS/MS analysis, when bacteria were encountered in the pesticide solution. In this study, we noticed that when identifying bacteria at the species level, not only genomic analyses alone, but also multilocus sequencing analysis should be performed. When genomic analyses were performed by searching the contig sequences of the bacteria, it was determined that the strain was classified as *Serratia marcescens*, but in subsequent analyses, it was also closely related to *S. nematodiphila*. Based on this finding, it was necessary to expand the analyses and examine allelic variations using MLST analysis. We confirmed that this strain was identified as *S. sarumanii*.

This study demonstrated for the first time that the genetic structure of *S. sarumanii* can produce enzymes that degrade various active pesticide compounds. The finding reveals that the potential of the strain owing to its biodegradation property can be integrated into pest management and chemical practice to reduce environmental pollution. Therefore, this investigation could be a piooner study on *S. sarumanii*’s microbial biodegradation property. A comprehensive understanding of the enzymes involved in these metabolic pathways linked to biodegradation capacity will enable the application of metabolic engineering or DNA recombinant techniques to use these biomolecules independently of microorganisms. Analyzing the genes involved in pesticide biodegradation is crucial for understanding this process. By exploring the genetic basis of pesticide biodegradation, we can not only trace the evolutionary history of this process but also engineer microorganisms with enhanced decomposer capabilities and develop innovative bioremediation strategies for contaminated soils, sediments, and water.

## Conclusion

Our study demonstrated the potential of the *Serratia sarumanii* strain as a promising agent for pesticide biodegradation in agricultural protective applications. The identification of microorganisms capable of degrading commonly used pesticides contributes significantly to environmental conservation efforts and underscores the importance of preserving ecological balance. The strain’s ability to degrade certain pesticide compounds while enhancing the efficacy of others suggests a dual benefit to sustainable agriculture. Furthermore, the genomic insights gained from this research pave the way for innovative bioremediation applications based on recombinant DNA technology. Overall, these findings underscore the critical role that microbial interventions can play in improving agricultural practices and minimizing environmental impacts.

## Supplementary Information

Below is the link to the electronic supplementary material.

Supplementary file1 (ZIP 20243 KB)

Supplementary file2 (PDF 365 KB)

Supplementary file3 (PDF 408 KB)

## Data Availability

The raw sequence data of this study are openly available in NCBI database (NCBI accession number: PRJNA784190). The data that supports the findings of this study are available in the supplementary material of this article.
