# Supplementary material for: Pesticide degradation capacity of a novel strain belonging to Serratia sarumanii with its genomic profile
Source: Biodegradation. 2025 Jun 1;36(3):49. doi: 10.1007/s10532-025-10144-2 (PMC12127232; doi:10.1007/s10532-025-10144-2)
Supplement: Supplementary file 1 — Supplementary file1 (ZIP 20243 KB) [file 10532_2025_10144_MOESM1_ESM.zip › Supplementary data1.pdf]

SEED Viewer version 2.0

Welcome to the SEED Viewer - a read-only browser of the curated SEED data.
For more information about The SEED please visit theSEED.org.

»Navigate »Organism »Comparative Tools »Help find Omur Baysal

Organism Overview for Proteobacteria Serratia GBS19 (23480194.3)

**Genome**	Proteobacteria Serratia GBS19 (Taxonomy ID: 23480194)
**Domain**	Bacteria
**Taxonomy**	Bacteria; Proteobacteria Serratia GBS19
**Neighbors**	View closest neighbors
**Size**	10,167,833
**GC Content**	59.9
**N50**	35605
**L50**	74
**Number of Contigs (with PEGs)**	658
**Number of Subsystems**	387
**Number of Coding Sequences**	10280
**Number of RNAs**	132

For each genome we offer a wide set of information to browse, compare and download.

Browse | Compare | Download | Annotate

Browse through the features of Proteobacteria Serratia GBS19 both graphically and through a table. Both allow quick navigation and filtering for features of your interest. Each feature is linked to its own detail page.

Click here to get to the Genome Browser

Subsystem Information

As an annotator you have the option of recomputing the subsystems for this genome, based on the current annotations. The computation will take several minutes. You can revert to the previous version of subsystem calculation by clicking the 'revert to last version' button (only available if a previous version exists).

recompute subsystems revert to last version

Subsystem Statistics | Features in Subsystems

Subsystem Coverage **Subsystem Category Distribution** **Subsystem Feature Counts**

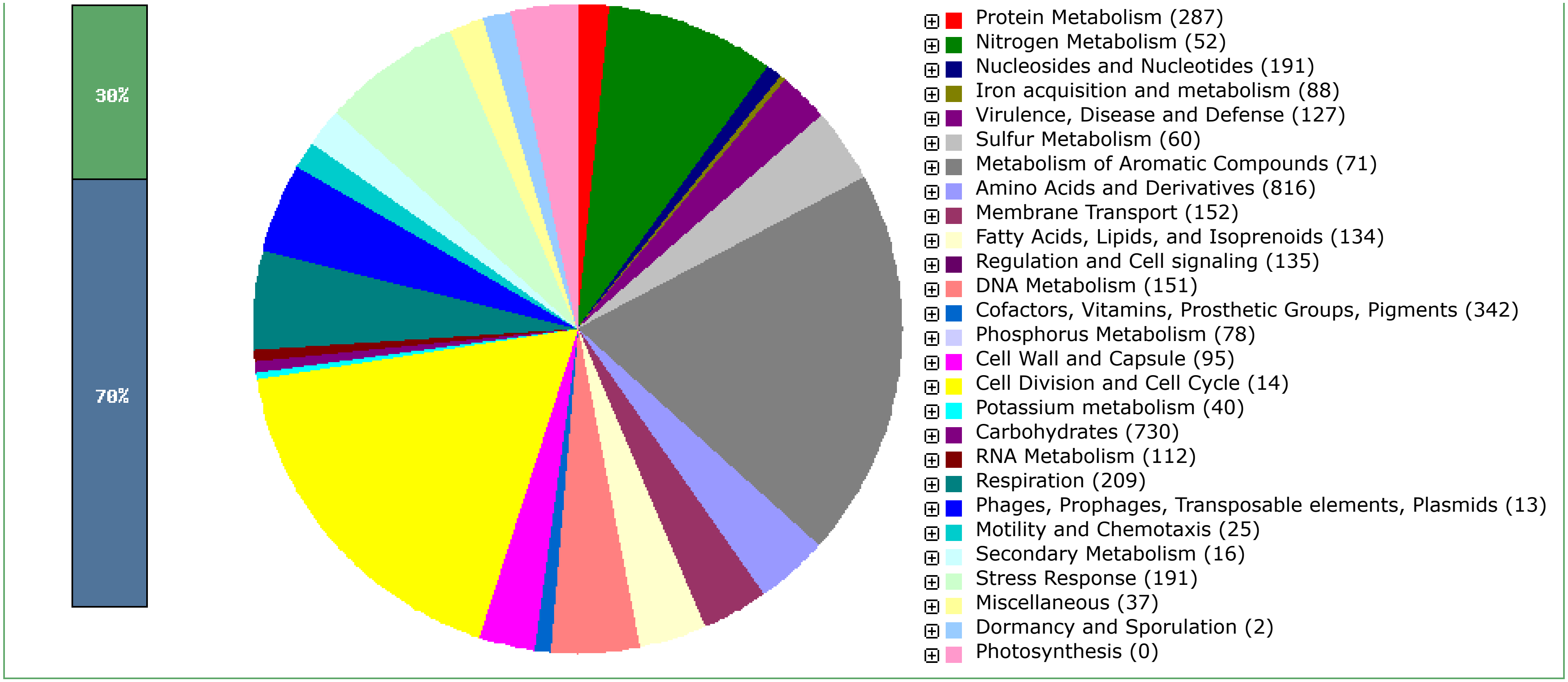
30%
70%
Protein Metabolism (287)
Nitrogen Metabolism (52)
Nucleosides and Nucleotides (191)
Iron acquisition and metabolism (88)
Virulence, Disease and Defense (127)
Sulfur Metabolism (60)
Metabolism of Aromatic Compounds (71)
Amino Acids and Derivatives (816)
Membrane Transport (152)
Fatty Acids, Lipids, and Isoprenoids (134)
Regulation and Cell signaling (135)
DNA Metabolism (151)
Cofactors, Vitamins, Prosthetic Groups, Pigments (342)
Phosphorus Metabolism (78)
Cell Wall and Capsule (95)
Cell Division and Cell Cycle (14)
Potassium metabolism (40)
Carbohydrates (730)
RNA Metabolism (112)
Respiration (209)
Phages, Prophages, Transposable elements, Plasmids (13)
Motility and Chemotaxis (25)
Secondary Metabolism (16)
Stress Response (191)
Miscellaneous (37)
Dormancy and Sporulation (2)
Photosynthesis (0)
