# Supplementary material for: Pesticide degradation capacity of a novel strain belonging to Serratia sarumanii with its genomic profile
Source: Biodegradation. 2025 Jun 1;36(3):49. doi: 10.1007/s10532-025-10144-2 (PMC12127232; doi:10.1007/s10532-025-10144-2)
Supplement: Supplementary file 1 — Supplementary file1 (ZIP 20243 KB) [file 10532_2025_10144_MOESM1_ESM.zip › Supplementary data2.pdf]

Compound Calibration Report

Lab Name: Default Laboratory
Instrument: Thermo Scientific Instrument
User: Quantiva_2
Batch: Karisim_Projesi_

Method: Karisim_Projesi__Karisim_Projesi_2020
Karisim_Projesi_2020
Cali File: Karisim_Projesi_.calx

Compound Name: **Cypermethrin**

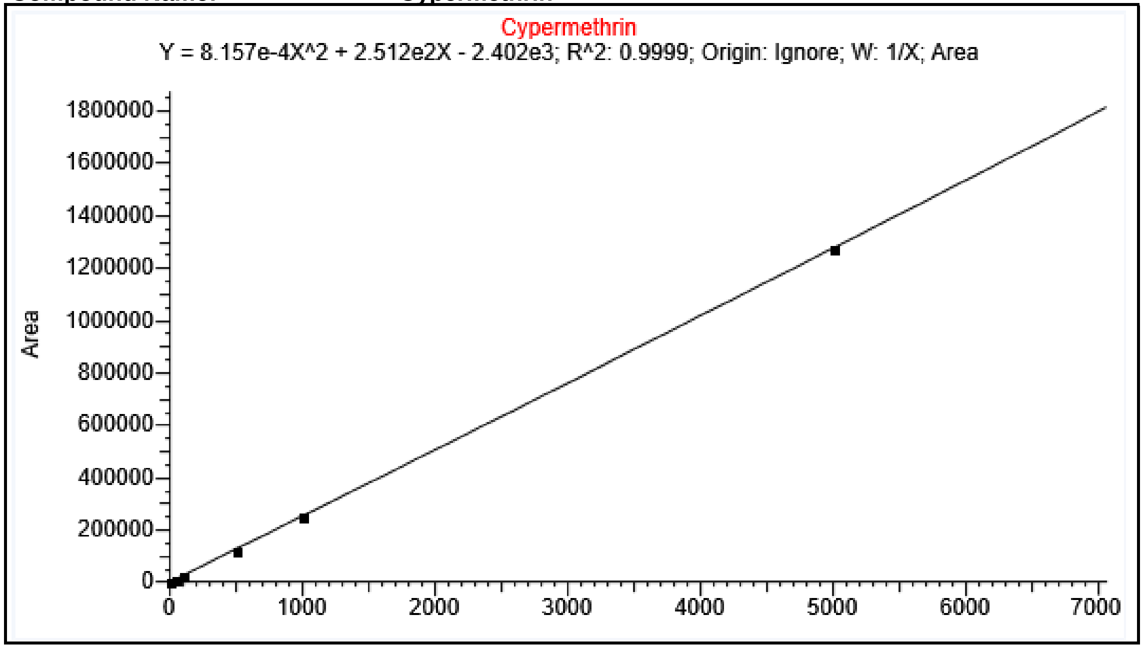

Quadratic
Pass

Level	Std Amount	Std Area	IS Amount	IS Area	Resp factor/ ratio	Calc Amount	Units	% CV	% RSD
3	10	309			30.856	10.788		N/A	N/A
5	50	9325			186.503	46.674		N/A	N/A
6	100	22481			224.807	99.017		N/A	N/A
8	500	121566			243.133	492.691		N/A	N/A
9	1000	252789			252.789	1012.507		N/A	N/A
10	5000	1273617			254.723	4998.321		N/A	N/A

Bordered cell = Manually Integrated; Calibration flags: D=RSD; F=Response factor; R=R Squared; A=Amount.

Compound Calibration Report

Lab Name: Default Laboratory
Instrument: Thermo Scientific Instrument
User: Quantiva_2
Batch: Karisim_Projesi_

Method: Karisim_Projesi__Karisim_Projesi_2020
Karisim_Projesi_2020
Cali File: Karisim_Projesi_.calx

Compound Name: **LambdaCyhalothrin**

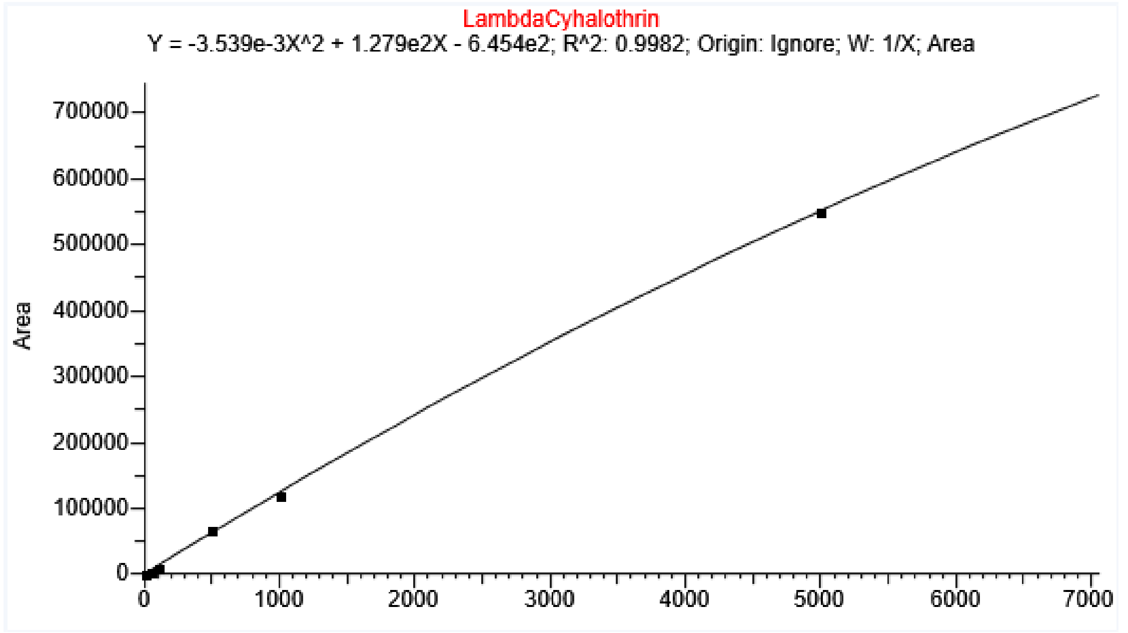

Quadratic
Pass

Level	Std Amount	Std Area	IS Amount	IS Area	Resp factor/ ratio	Calc Amount	Units	% CV	% RSD
3	10	863			86.296	11.798		N/A	N/A
5	50	5201			104.028	45.776		N/A	N/A
6	100	10050			100.496	83.823		N/A	N/A
8	500	68613			137.226	549.926		N/A	N/A
9	1000	119630			119.63	966.32		N/A	N/A
10	5000	550559			110.112	5002.603		N/A	N/A

Bordered cell = Manually Integrated; Calibration flags: D=RSD; F=Response factor; R=R Squared; A=Amount.

Compound Calibration Report

Lab Name: Default Laboratory
Instrument: Thermo Scientific Instrument
User: Quantiva_2
Batch: Karisim_Projesi_

Method: Karisim_Projesi__Karisim_Projesi_2020
Karisim_Projesi_2020
Cali File: Karisim_Projesi_.calx

Compound Name: **Thiophanate-Methyl**

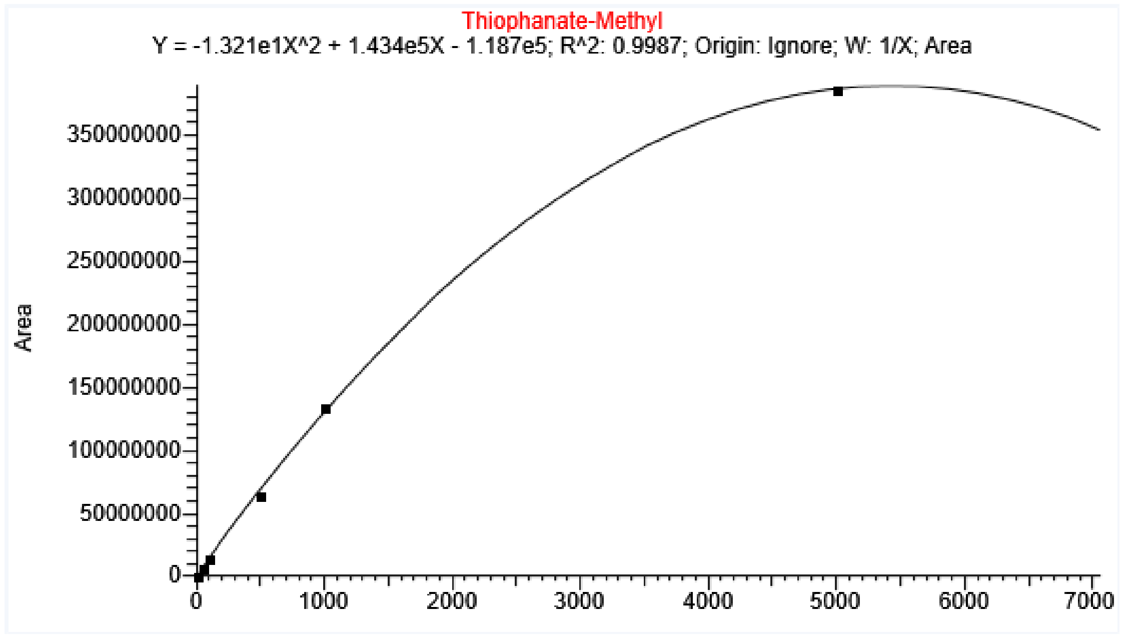

Quadratic
Pass

Level	Std Amount	Std Area	IS Amount	IS Area	Resp factor/ ratio	Calc Amount	Units	% CV	% RSD
3	10	1436378			143637.817	10.857		N/A	N/A
5	50	6472118			129442.367	46.167		N/A	N/A
6	100	14314448			143144.48	101.622		N/A	N/A
8	500	63943682			127887.364	466.922		N/A	N/A
9	1000	135140153			135140.153	1043.839		N/A	N/A
10	5000	385808103			77161.621	4950.567		N/A	N/A

Bordered cell = Manually Integrated; Calibration flags: D=RSD; F=Response factor; R=R Squared; A=Amount.

Compound Calibration Report

Lab Name: Default Laboratory
Instrument: Thermo Scientific Instrument
User: Quantiva_2
Batch: Karisim_Projesi_

Method: Karisim_Projesi__Karisim_Projesi_2020
Karisim_Projesi_2020
Cali File: Karisim_Projesi_.calx

Compound Name: **Chlorantraniliprole**

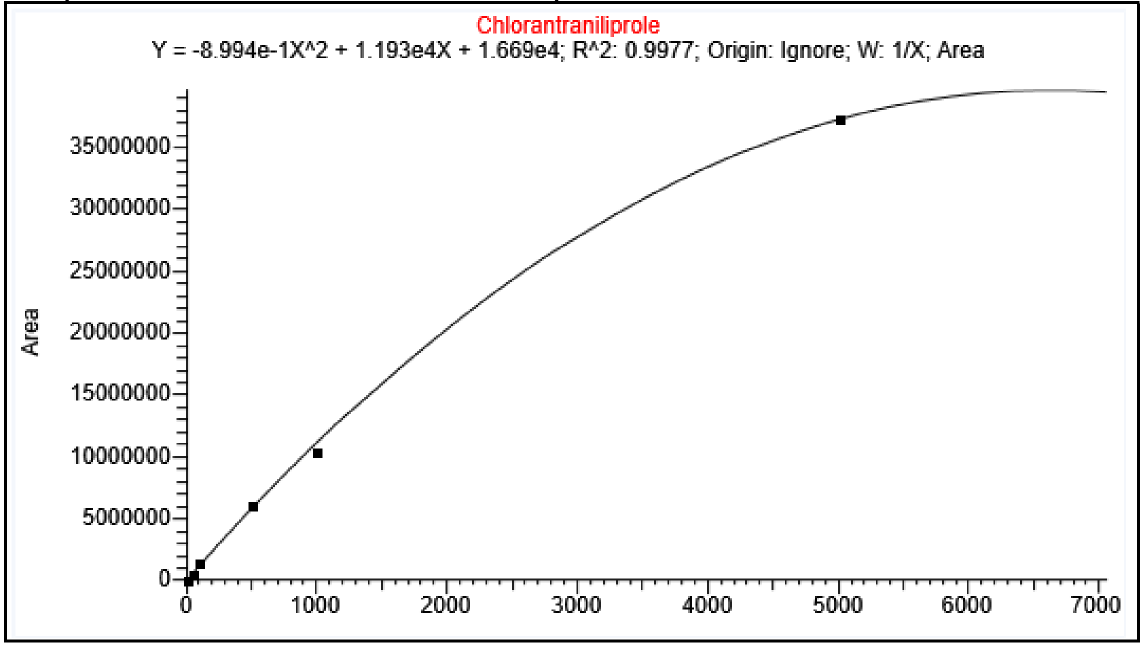

Quadratic
Pass

Level	Std Amount	Std Area	IS Amount	IS Area	Resp factor/ ratio	Calc Amount	Units	% CV	% RSD
3	10	120786			12078.644	8.731		N/A	N/A
5	50	582079			11641.572	47.557		N/A	N/A
6	100	1394783			13947.831	116.523		N/A	N/A
8	500	6122477			12244.953	533.159		N/A	N/A
9	1000	10455573			10455.573	941.747		N/A	N/A
10	5000	37267617			7453.523	5026.99		N/A	N/A

Bordered cell = Manually Integrated; Calibration flags: D=RSD; F=Response factor; R=R Squared; A=Amount.

Compound Calibration Report

Lab Name: Default Laboratory
Instrument: Thermo Scientific Instrument
User: Quantiva_2
Batch: Karisim_Projesi_

Method: Karisim_Projesi__Karisim_Projesi_2020
Karisim_Projesi_2020
Cali File: Karisim_Projesi_.calx

Compound Name: **Azoxystrobin**

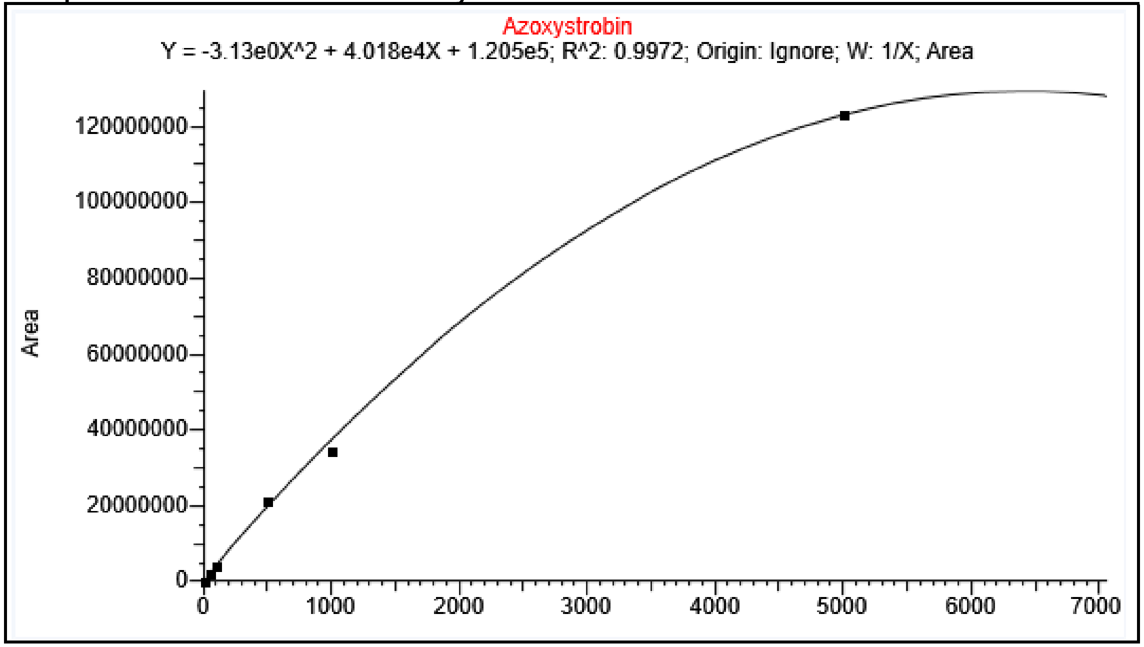

Quadratic
Pass

Level	Std Amount	Std Area	IS Amount	IS Area	Resp factor/ ratio	Calc Amount	Units	% CV	% RSD
3	10	471438			47143.828	8.74		N/A	N/A
5	50	2168657			43373.144	51.175		N/A	N/A
6	100	4383057			43830.57	106.97		N/A	N/A
8	500	21300666			42601.333	550.712		N/A	N/A
9	1000	34736675			34736.675	928.623		N/A	N/A
10	5000	123088525			24617.705	5033.536		N/A	N/A

Bordered cell = Manually Integrated; Calibration flags: D=RSD; F=Response factor; R=R Squared; A=Amount.

Compound Calibration Report

Lab Name: Default Laboratory
Instrument: Thermo Scientific Instrument
User: Quantiva_2
Batch: Karisim_Projesi_

Method: Karisim_Projesi__Karisim_Projesi_2020
Karisim_Projesi_2020
Cali File: Karisim_Projesi_.calx

Compound Name: **Pyrimethanil**

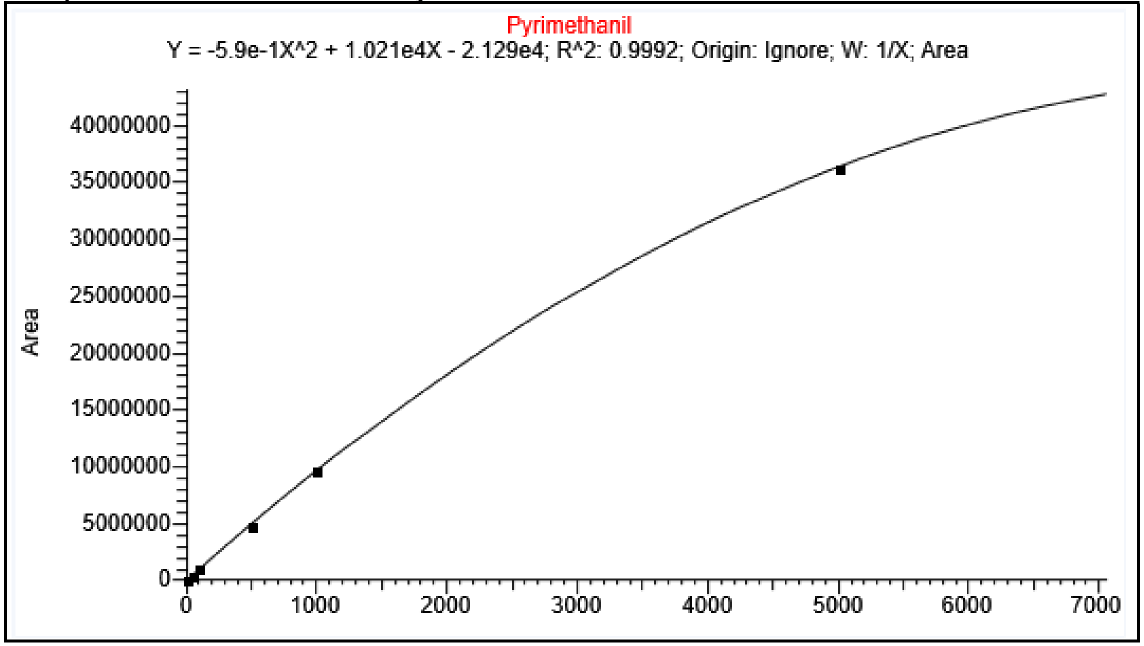

Quadratic
Pass

Level	Std Amount	Std Area	IS Amount	IS Area	Resp factor/ ratio	Calc Amount	Units	% CV	% RSD
3	10	76891			7689.052	9.621		N/A	N/A
5	50	447253			8945.061	46.009		N/A	N/A
6	100	1140066			11400.664	114.493		N/A	N/A
8	500	4776135			9552.271	483.328		N/A	N/A
9	1000	9656259			9656.259	1006.267		N/A	N/A
10	5000	36285202			7257.04	5000.518		N/A	N/A

Bordered cell = Manually Integrated; Calibration flags: D=RSD; F=Response factor; R=R Squared; A=Amount.

Compound Calibration Report

Lab Name: Default Laboratory
Instrument: Thermo Scientific Instrument
User: Quantiva_2
Batch: Karisim_Projesi_

Method: Karisim_Projesi__Karisim_Projesi_2020
Karisim_Projesi_2020
Cali File: Karisim_Projesi_.calx

Compound Name: **Boscalid**

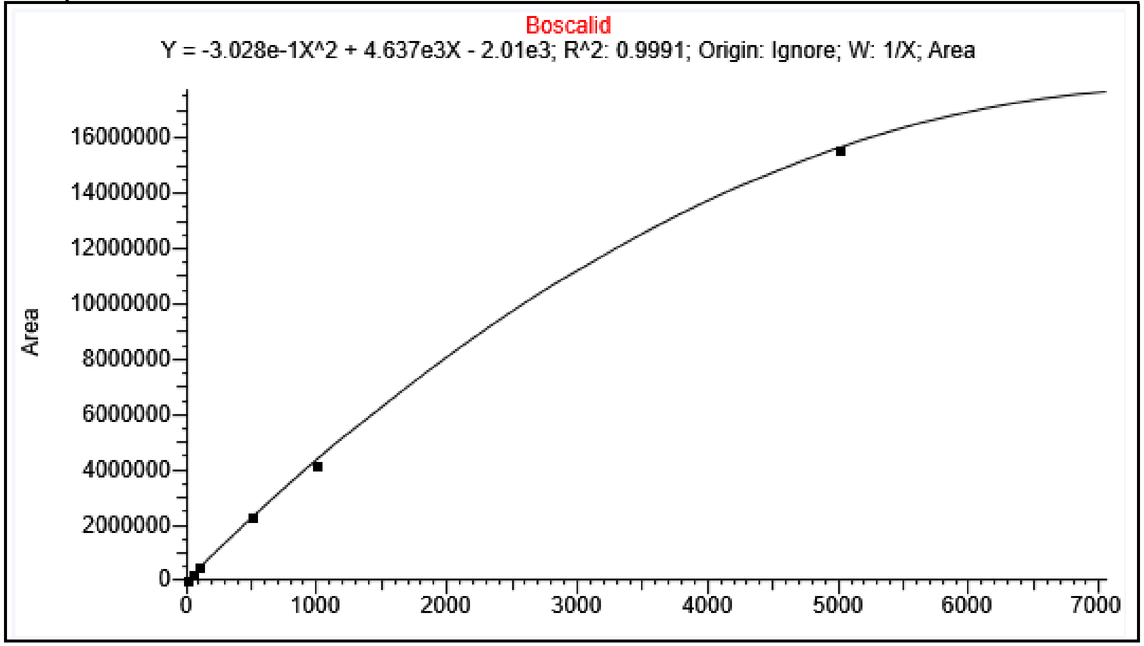

Quadratic
Pass

Level	Std Amount	Std Area	IS Amount	IS Area	Resp factor/ ratio	Calc Amount	Units	% CV	% RSD
3	10	42770			4277.028	9.664		N/A	N/A
5	50	210778			4215.556	46.029		N/A	N/A
6	100	504859			5048.594	110.104		N/A	N/A
8	500	2341429			4682.858	523.273		N/A	N/A
9	1000	4186743			4186.743	964.053		N/A	N/A
10	5000	15629649			3125.93	5011.372		N/A	N/A

Bordered cell = Manually Integrated; Calibration flags: D=RSD; F=Response factor; R=R Squared; A=Amount.

Compound Calibration Report

Lab Name: Default Laboratory
Instrument: Thermo Scientific Instrument
User: Quantiva_2
Batch: Karisim_Projesi_

Method: Karisim_Projesi__Karisim_Projesi_2020
Karisim_Projesi_2020
Cali File: Karisim_Projesi_.calx

Compound Name: **Dimethomorph**

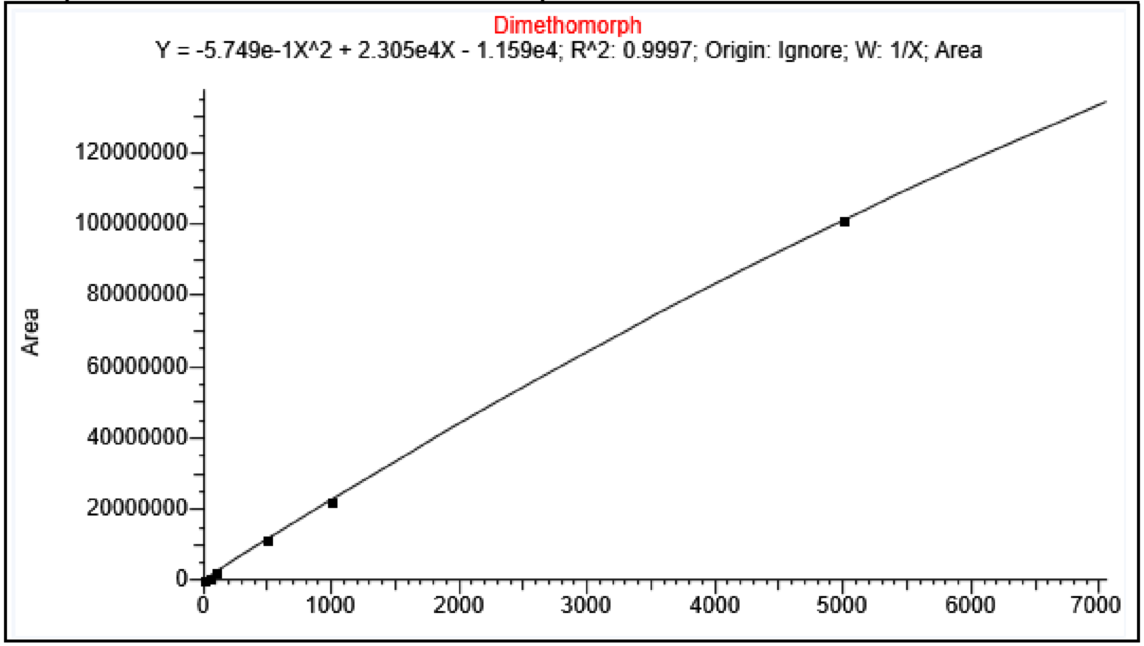

Quadratic
Pass

Level	Std Amount	Std Area	IS Amount	IS Area	Resp factor/ ratio	Calc Amount	Units	% CV	% RSD
3	10	233153			23315.315	10.621		N/A	N/A
5	50	1013632			20272.642	44.529		N/A	N/A
6	100	2354176			23541.761	102.902		N/A	N/A
8	500	11775991			23551.983	518.096		N/A	N/A
9	1000	22062112			22062.112	981.698		N/A	N/A
10	5000	100903493			20180.699	5002.28		N/A	N/A

Bordered cell = Manually Integrated; Calibration flags: D=RSD; F=Response factor; R=R Squared; A=Amount.

Compound Calibration Report

Lab Name: Default Laboratory
Instrument: Thermo Scientific Instrument
User: Quantiva_2
Batch: Karisim_Projesi_

Method: Karisim_Projesi__Karisim_Projesi_2020
Karisim_Projesi_2020
Cali File: Karisim_Projesi_.calx

Compound Name: **Fludioxonil**

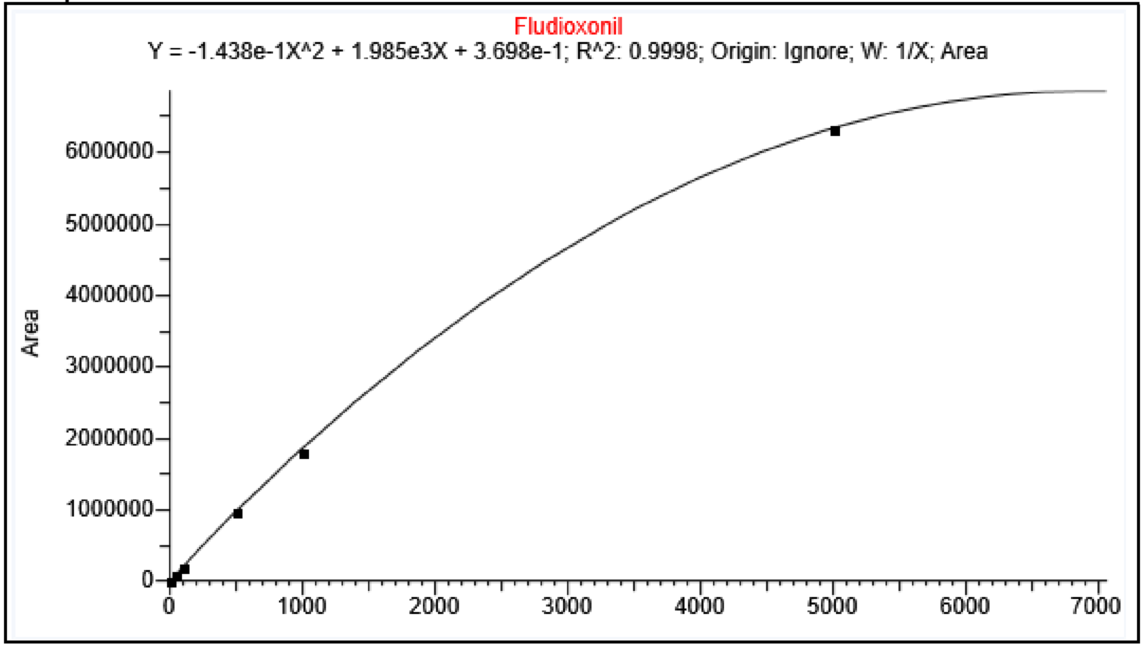

Quadratic
Pass

Level	Std Amount	Std Area	IS Amount	IS Area	Resp factor/ ratio	Calc Amount	Units	% CV	% RSD
3	10	19616			1961.61	9.89		N/A	N/A
5	50	94882			1897.63	47.97		N/A	N/A
6	100	206557			2065.567	104.864		N/A	N/A
8	500	970866			1941.732	507.824		N/A	N/A
9	1000	1818415			1818.415	986.686		N/A	N/A
10	5000	6331986			1266.397	5005.375		N/A	N/A

Bordered cell = Manually Integrated; Calibration flags: D=RSD; F=Response factor; R=R Squared; A=Amount.

Compound Calibration Report

Lab Name: Default Laboratory
Instrument: Thermo Scientific Instrument
User: Quantiva_2
Batch: Karisim_Projesi_

Method: Karisim_Projesi__Karisim_Projesi_2020
Karisim_Projesi_2020
Cali File: Karisim_Projesi_.calx

Compound Name: **Fluopyram**

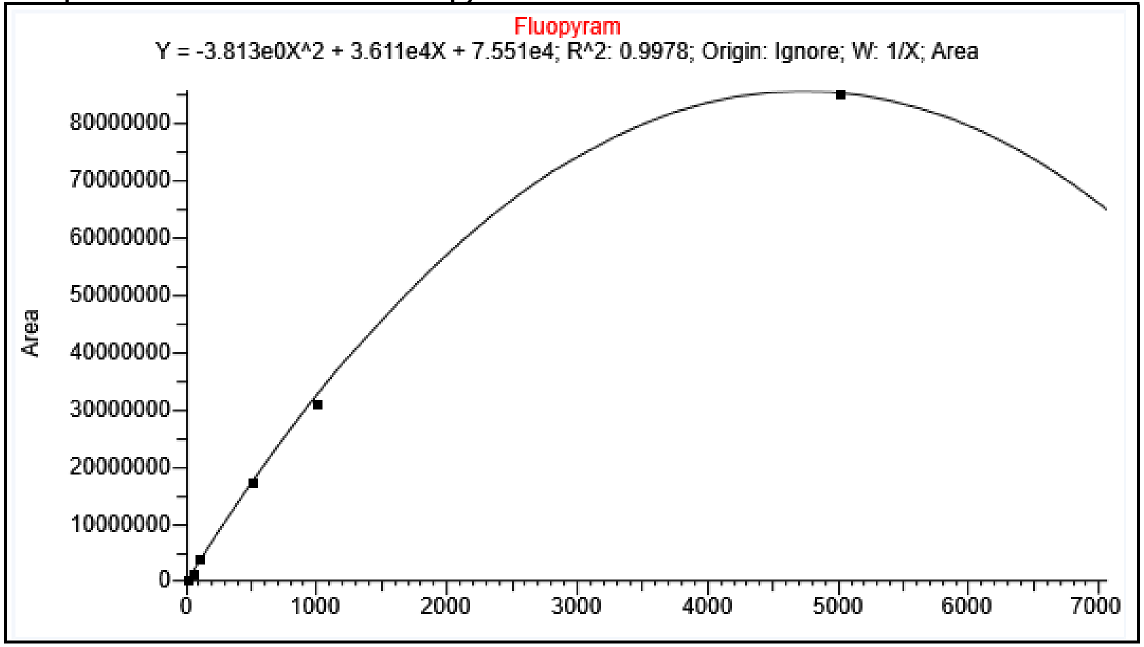

Quadratic
Pass

Level	Std Amount	Std Area	IS Amount	IS Area	Resp factor/ ratio	Calc Amount	Units	% CV	% RSD
3	10	415569			41556.95	9.427		N/A	N/A
5	50	1651863			33037.264	43.858		N/A	N/A
6	100	4299393			42993.929	118.457		N/A	N/A
8	500	17652179			35304.359	514.744		N/A	N/A
9	1000	31336723			31336.723	963.842		N/A	N/A
10	5000	85442087			17088.417	4557.896		N/A	N/A

Bordered cell = Manually Integrated; Calibration flags: D=RSD; F=Response factor; R=R Squared; A=Amount.

Compound Calibration Report

Lab Name: Default Laboratory
Instrument: Thermo Scientific Instrument
User: Quantiva_2
Batch: Karisim_Projesi_

Method: Karisim_Projesi__Karisim_Projesi_2020
Karisim_Projesi_2020
Cali File: Karisim_Projesi_.calx

Compound Name: **Spirotetramat**

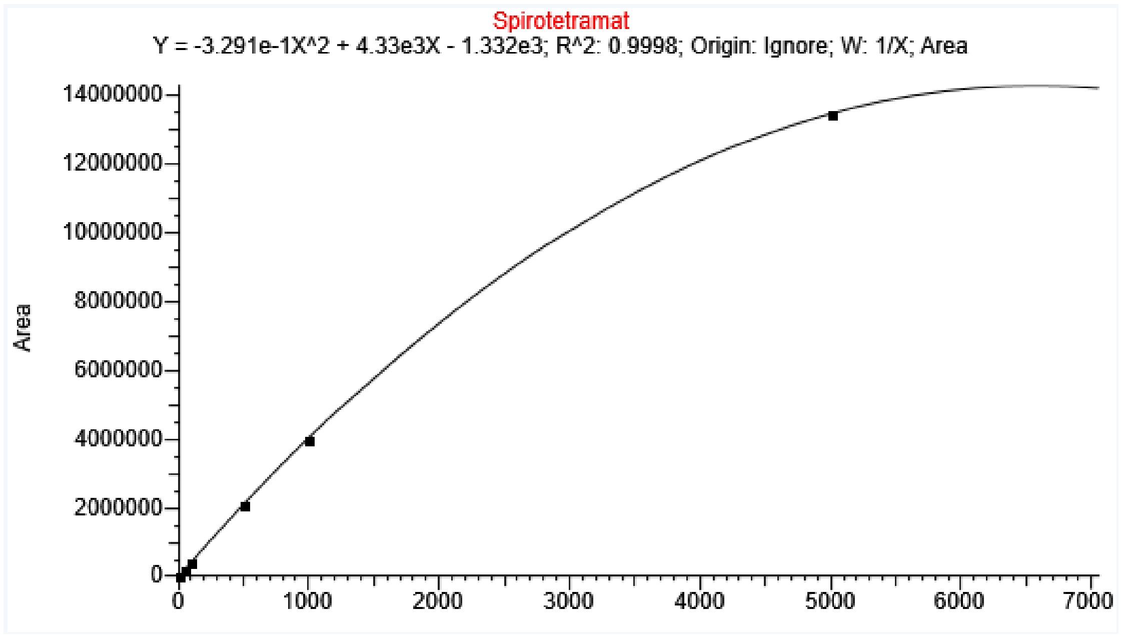

Quadratic
Pass

Level	Std Amount	Std Area	IS Amount	IS Area	Resp factor/ ratio	Calc Amount	Units	% CV	% RSD
3	10	43661			4366.069	10.4		N/A	N/A
5	50	217117			4342.349	50.65		N/A	N/A
6	100	400005			4000.053	93.359		N/A	N/A
8	500	2110520			4221.04	507.336		N/A	N/A
9	1000	3995265			3995.265	998.935		N/A	N/A
10	5000	13418229			2683.646	4998.445		N/A	N/A

Bordered cell = Manually Integrated; Calibration flags: D=RSD; F=Response factor; R=R Squared; A=Amount.

Compound Calibration Report

Lab Name: Default Laboratory
Instrument: Thermo Scientific Instrument
User: Quantiva_2
Batch: Karisim_Projesi_

Method: Karisim_Projesi__Karisim_Projesi_2020
Karisim_Projesi_2020
Cali File: Karisim_Projesi_.calx

Compound Name: **Fenhexamid**

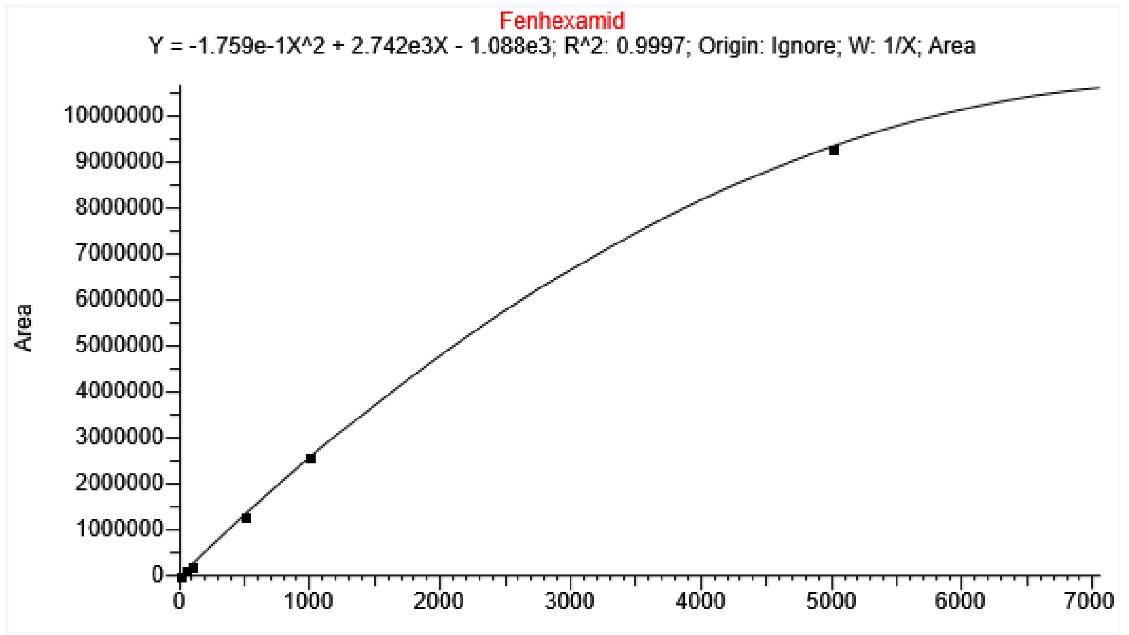

Quadratic
Pass

Level	Std Amount	Std Area	IS Amount	IS Area	Resp factor/ ratio	Calc Amount	Units	% CV	% RSD
3	10	27883			2788.274	10.573		N/A	N/A
5	50	139756			2795.124	51.536		N/A	N/A
6	100	245323			2453.227	90.391		N/A	N/A
8	500	1321330			2642.66	498.211		N/A	N/A
9	1000	2594895			2594.895	1012.526		N/A	N/A
10	5000	9306370			1861.274	4994.87		N/A	N/A

Bordered cell = Manually Integrated; Calibration flags: D=RSD; F=Response factor; R=R Squared; A=Amount.

Compound Calibration Report

Lab Name: Default Laboratory
Instrument: Thermo Scientific Instrument
User: Quantiva_2
Batch: Karisim_Projesi_

Method: Karisim_Projesi__Karisim_Projesi_2020
Karisim_Projesi_2020
Cali File: Karisim_Projesi_.calx

Compound Name: **Cyprodinil**

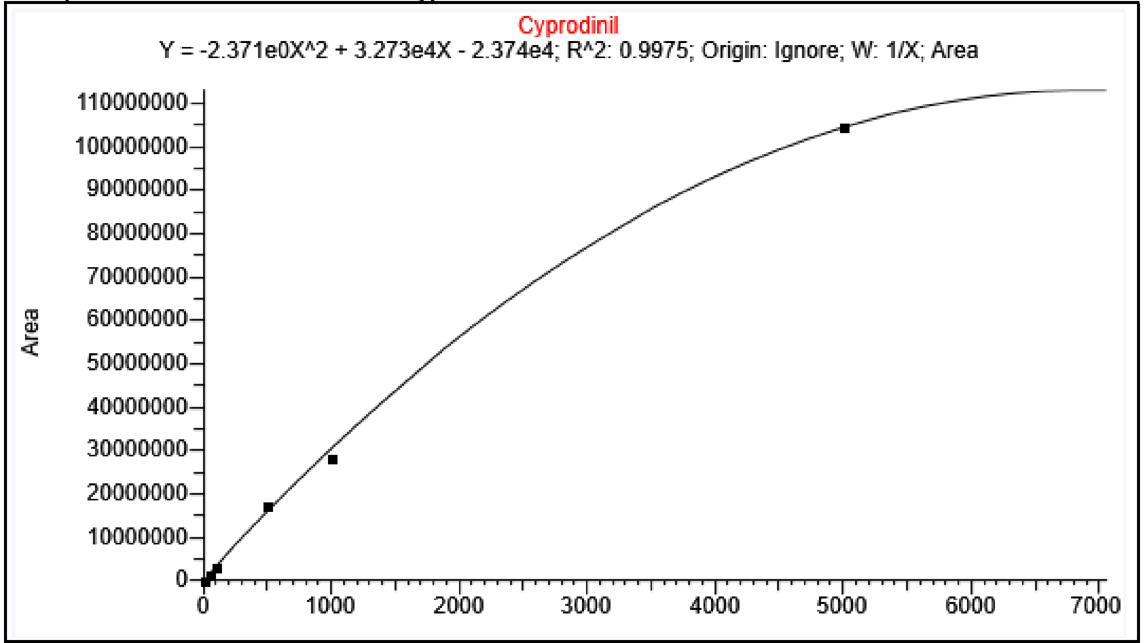

Quadratic
Pass

Level	Std Amount	Std Area	IS Amount	IS Area	Resp factor/ ratio	Calc Amount	Units	% CV	% RSD
3	10	299284			29928.389	9.875		N/A	N/A
5	50	1482507			29650.147	46.17		N/A	N/A
6	100	3375933			33759.328	104.653		N/A	N/A
8	500	17328761			34657.522	552.201		N/A	N/A
9	1000	28538412			28538.412	936.026		N/A	N/A
10	5000	104572210			20914.442	5022.347		N/A	N/A

Bordered cell = Manually Integrated; Calibration flags: D=RSD; F=Response factor; R=R Squared; A=Amount.

Compound Calibration Report

Lab Name: Default Laboratory
Instrument: Thermo Scientific Instrument
User: Quantiva_2
Batch: Karisim_Projesi_

Method: Karisim_Projesi__Karisim_Projesi_2020
Karisim_Projesi_2020
Cali File: Karisim_Projesi_.calx

Compound Name: **Penconazole**

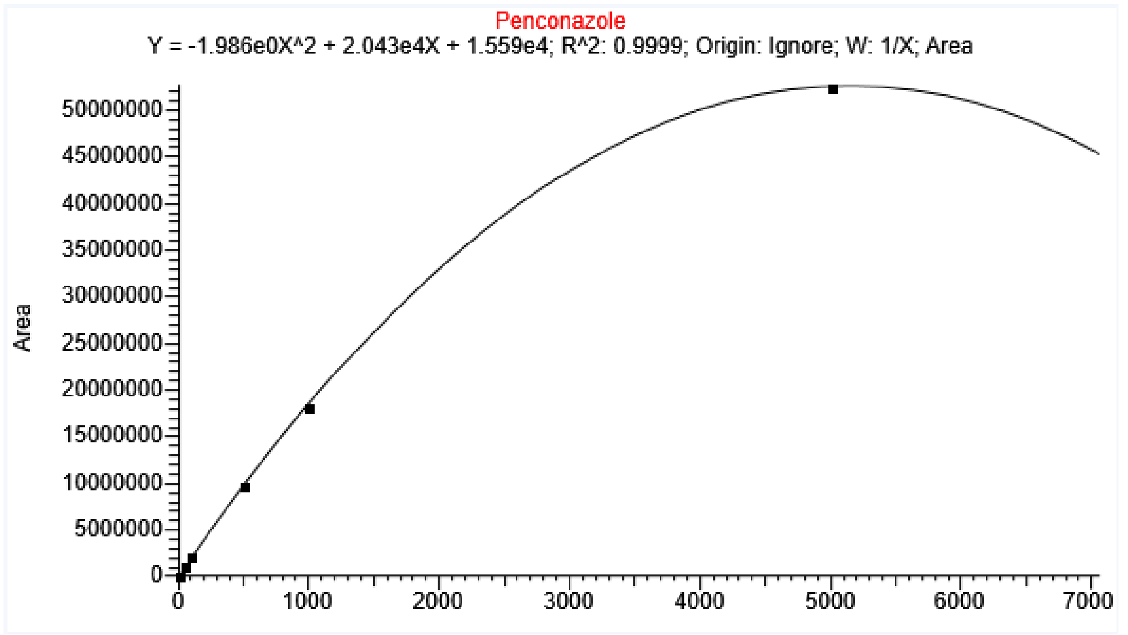

Quadratic
Pass

Level	Std Amount	Std Area	IS Amount	IS Area	Resp factor/ ratio	Calc Amount	Units	% CV	% RSD
3	10	204842			20484.235	9.272		N/A	N/A
5	50	1079760			21595.208	52.354		N/A	N/A
6	100	2096299			20962.994	102.872		N/A	N/A
8	500	9805803			19611.606	503.875		N/A	N/A
9	1000	18268968			18268.968	988.398		N/A	N/A
10	5000	52550022			10510.004	5067.432		N/A	N/A

Bordered cell = Manually Integrated; Calibration flags: D=RSD; F=Response factor; R=R Squared; A=Amount.

Compound Calibration Report

Lab Name: Default Laboratory
Instrument: Thermo Scientific Instrument
User: Quantiva_2
Batch: Karisim_Projesi_

Method: Karisim_Projesi__Karisim_Projesi_2020
Karisim_Projesi_2020
Cali File: Karisim_Projesi_.calx

Compound Name: **Tebuconazole**

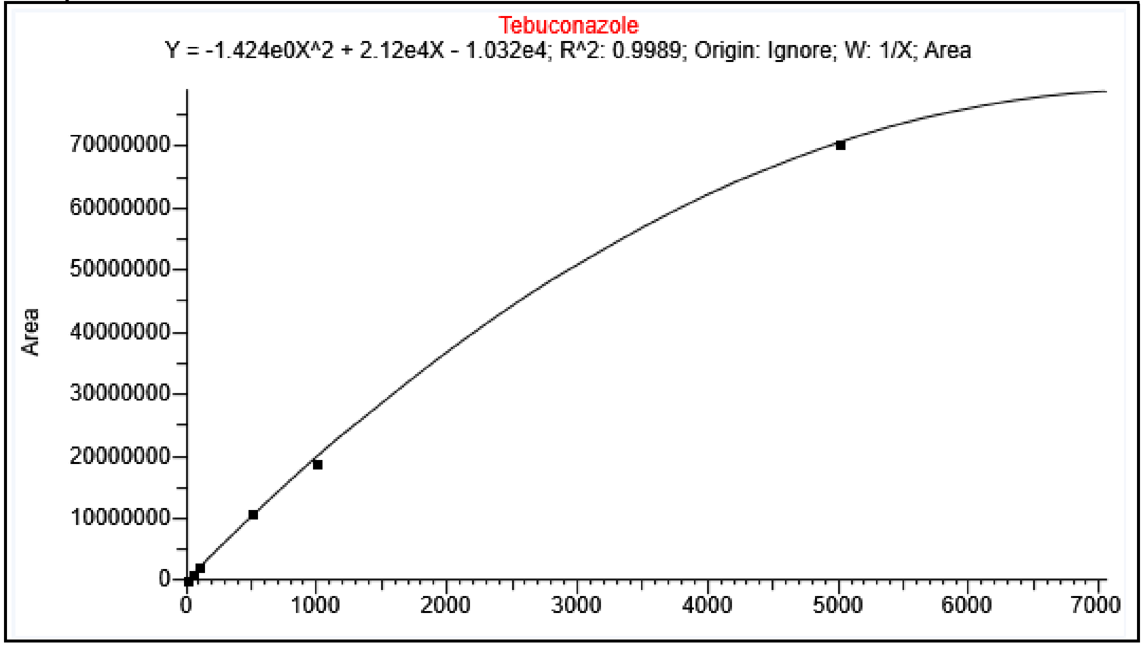

Quadratic
Pass

Level	Std Amount	Std Area	IS Amount	IS Area	Resp factor/ ratio	Calc Amount	Units	% CV	% RSD
3	10	187285			18728.524	9.325		N/A	N/A
5	50	1017164			20343.273	48.614		N/A	N/A
6	100	2257848			22578.484	107.744		N/A	N/A
8	500	10829799			21659.599	530.075		N/A	N/A
9	1000	18958487			18958.487	955.907		N/A	N/A
10	5000	70515358			14103.072	5014.422		N/A	N/A

Bordered cell = Manually Integrated; Calibration flags: D=RSD; F=Response factor; R=R Squared; A=Amount.

Compound Calibration Report

Lab Name: Default Laboratory
Instrument: Thermo Scientific Instrument
User: Quantiva_2
Batch: Karisim_Projesi_

Method: Karisim_Projesi__Karisim_Projesi_2020
Karisim_Projesi_2020
Cali File: Karisim_Projesi_.calx

Compound Name: **Pyraclostrobin**

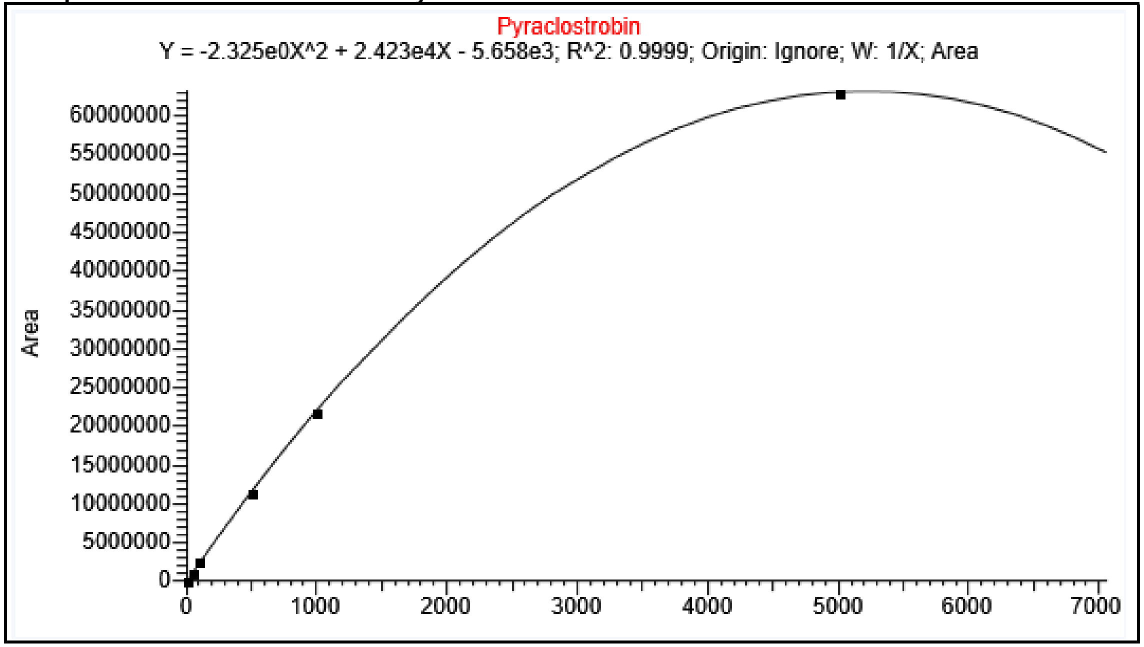

Quadratic
Pass

Level	Std Amount	Std Area	IS Amount	IS Area	Resp factor/ ratio	Calc Amount	Units	% CV	% RSD
3	10	235791			23579.089	9.976		N/A	N/A
5	50	1150608			23012.158	47.948		N/A	N/A
6	100	2511343			25113.428	104.951		N/A	N/A
8	500	11467833			22935.667	497.323		N/A	N/A
9	1000	21879872			21879.872	999.163		N/A	N/A
10	5000	63016698			12603.34	5007.572		N/A	N/A

Bordered cell = Manually Integrated; Calibration flags: D=RSD; F=Response factor; R=R Squared; A=Amount.

Compound Calibration Report

Lab Name: Default Laboratory
Instrument: Thermo Scientific Instrument
User: Quantiva_2
Batch: Karisim_Projesi_

Method: Karisim_Projesi__Karisim_Projesi_2020
Karisim_Projesi_2020
Cali File: Karisim_Projesi_.calx

Compound Name: **Metrafenone**

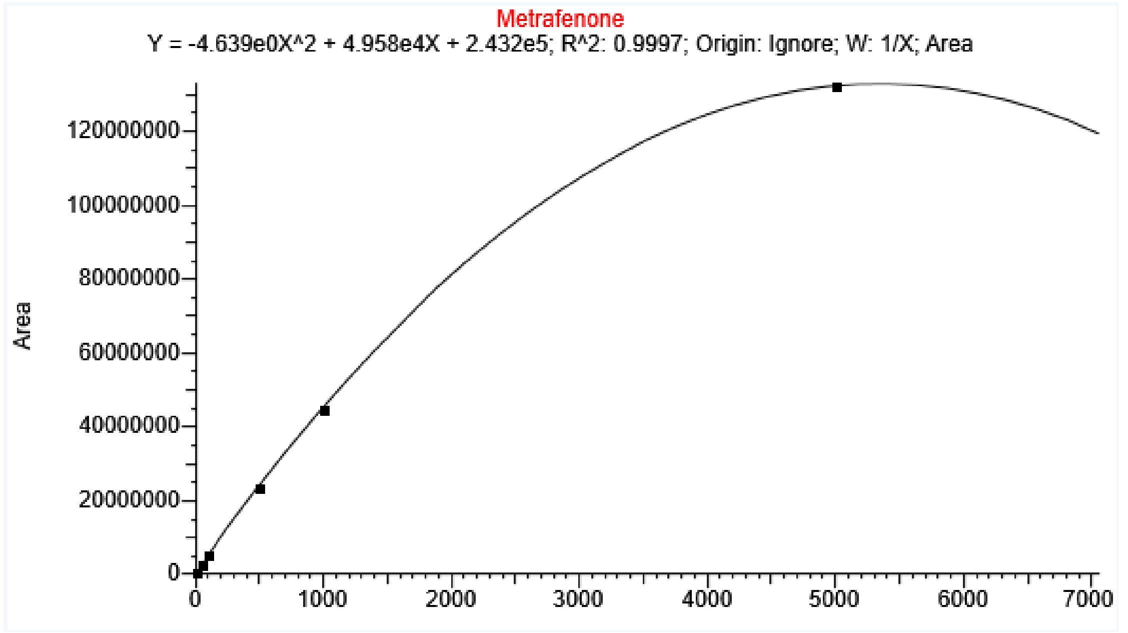

Quadratic
Pass

Level	Std Amount	Std Area	IS Amount	IS Area	Resp factor/ ratio	Calc Amount	Units	% CV	% RSD
3	10	696275			69627.542	9.146		N/A	N/A
5	50	2742684			54853.671	50.651		N/A	N/A
6	100	5565957			55659.574	108.453		N/A	N/A
8	500	23820857			47641.715	498.805		N/A	N/A
9	1000	44756675			44756.675	989.35		N/A	N/A
10	5000	132261209			26452.242	5027.026		N/A	N/A

Bordered cell = Manually Integrated; Calibration flags: D=RSD; F=Response factor; R=R Squared; A=Amount.

Compound Calibration Report

Lab Name: Default Laboratory
Instrument: Thermo Scientific Instrument
User: Quantiva_2
Batch: Karisim_Projesi_

Method: Karisim_Projesi__Karisim_Projesi_2020
Karisim_Projesi_2020
Cali File: Karisim_Projesi_.calx

Compound Name: **Difenconazole**

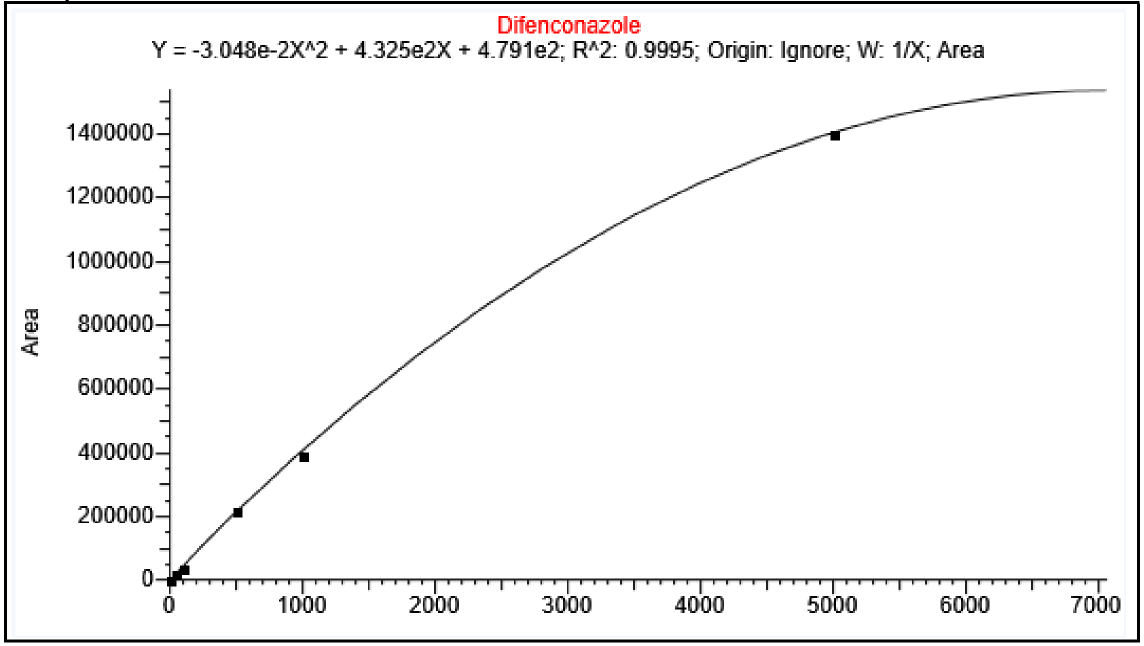

Quadratic
Pass

Level	Std Amount	Std Area	IS Amount	IS Area	Resp factor/ ratio	Calc Amount	Units	% CV	% RSD
3	10	5021			502.121	10.51		N/A	N/A
5	50	21417			428.332	48.578		N/A	N/A
6	100	41299			412.985	95.018		N/A	N/A
8	500	218515			437.03	523.452		N/A	N/A
9	1000	394984			394.984	979.837		N/A	N/A
10	5000	1401448			280.29	5004.766		N/A	N/A

Bordered cell = Manually Integrated; Calibration flags: D=RSD; F=Response factor; R=R Squared; A=Amount.

Compound Calibration Report

Lab Name: Default Laboratory
Instrument: Thermo Scientific Instrument
User: Quantiva_2
Batch: Karisim_Projesi_

Method: Karisim_Projesi__Karisim_Projesi_2020
Karisim_Projesi_2020
Cali File: Karisim_Projesi_.calx

Compound Name: **Indoxacarb**

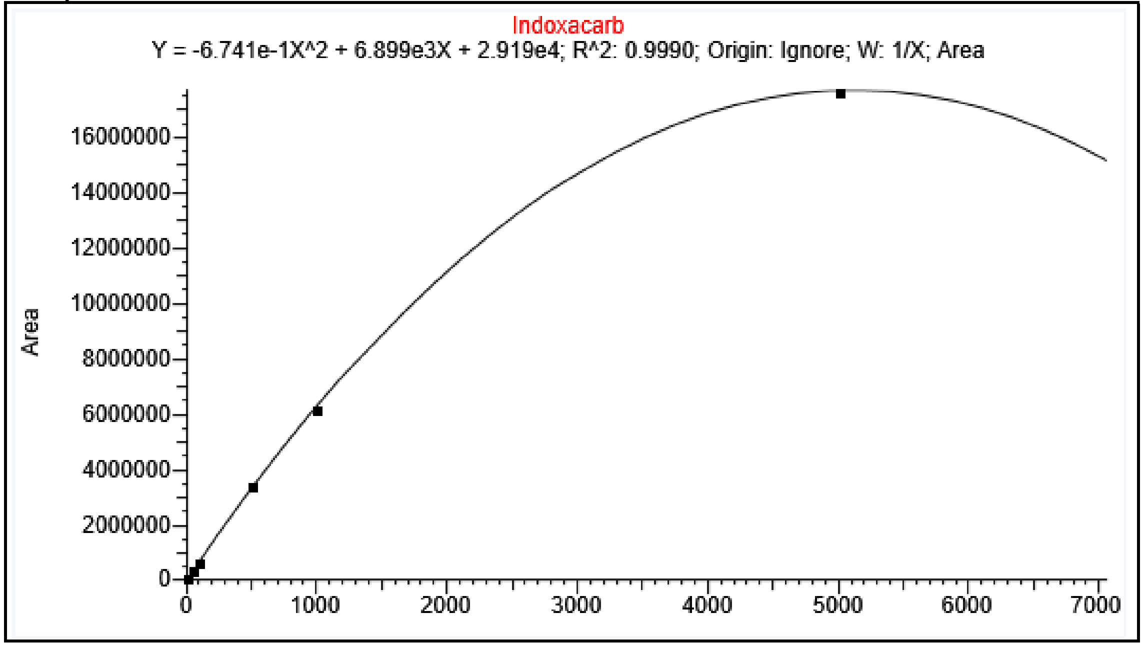

Quadratic
Pass

Level	Std Amount	Std Area	IS Amount	IS Area	Resp factor/ ratio	Calc Amount	Units	% CV	% RSD
3	10	97122			9712.209	9.856		N/A	N/A
5	50	411431			8228.614	55.708		N/A	N/A
6	100	630938			6309.383	87.979		N/A	N/A
8	500	3402130			6804.26	514.796		N/A	N/A
9	1000	6203371			6203.371	990.873		N/A	N/A
10	5000	17673152			3534.63	5014.817		N/A	N/A

Bordered cell = Manually Integrated; Calibration flags: D=RSD; F=Response factor; R=R Squared; A=Amount.

Compound Calibration Report

Lab Name: Default Laboratory
Instrument: Thermo Scientific Instrument
User: Quantiva_2
Batch: Karisim_Projesi_

Method: Karisim_Projesi__Karisim_Projesi_2020
Karisim_Projesi_2020
Cali File: Karisim_Projesi_.calx

Compound Name: **Spinetoram**

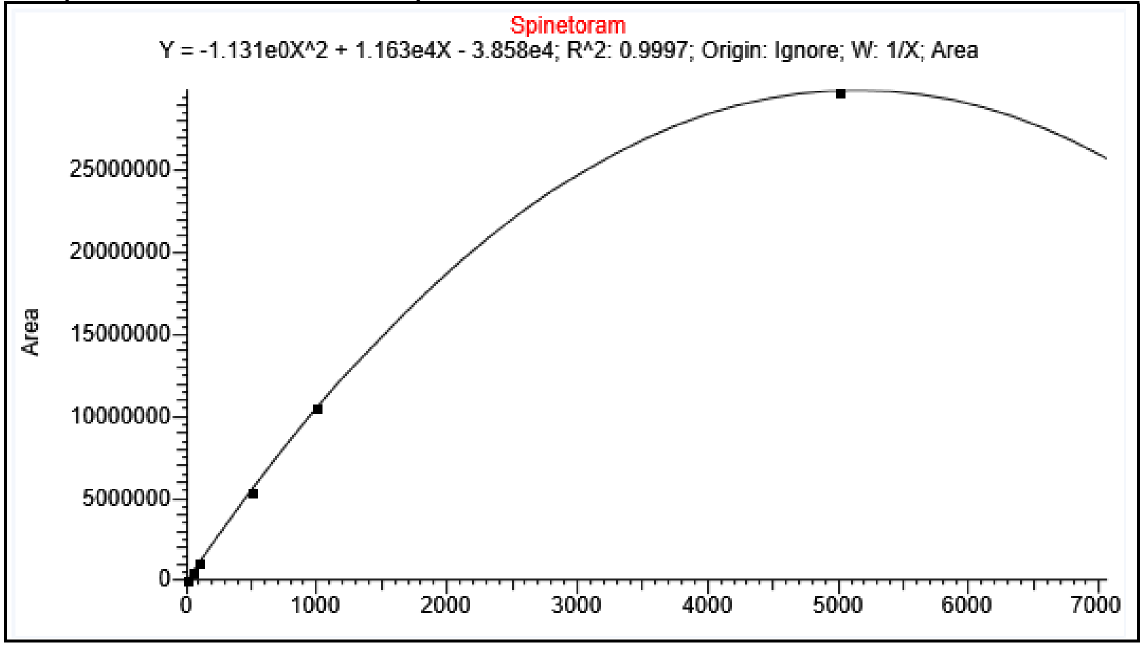

Quadratic
Pass

Level	Std Amount	Std Area	IS Amount	IS Area	Resp factor/ ratio	Calc Amount	Units	% CV	% RSD
3	10	89676			8967.632	11.041		N/A	N/A
5	50	494651			9893.02	46.059		N/A	N/A
6	100	1079021			10790.207	97.019		N/A	N/A
8	500	5449244			10898.487	495.809		N/A	N/A
9	1000	10591957			10591.957	1014.146		N/A	N/A
10	5000	29814154			5962.831	4945.212		N/A	N/A

Bordered cell = Manually Integrated; Calibration flags: D=RSD; F=Response factor; R=R Squared; A=Amount.

Compound Calibration Report

Lab Name: Default Laboratory
Instrument: Thermo Scientific Instrument
User: Quantiva_2
Batch: Karisim_Projesi_

Method: Karisim_Projesi__Karisim_Projesi_2020
Karisim_Projesi_2020
Cali File: Karisim_Projesi_.calx

Compound Name: **Emamectin-B1B-Benzoate**

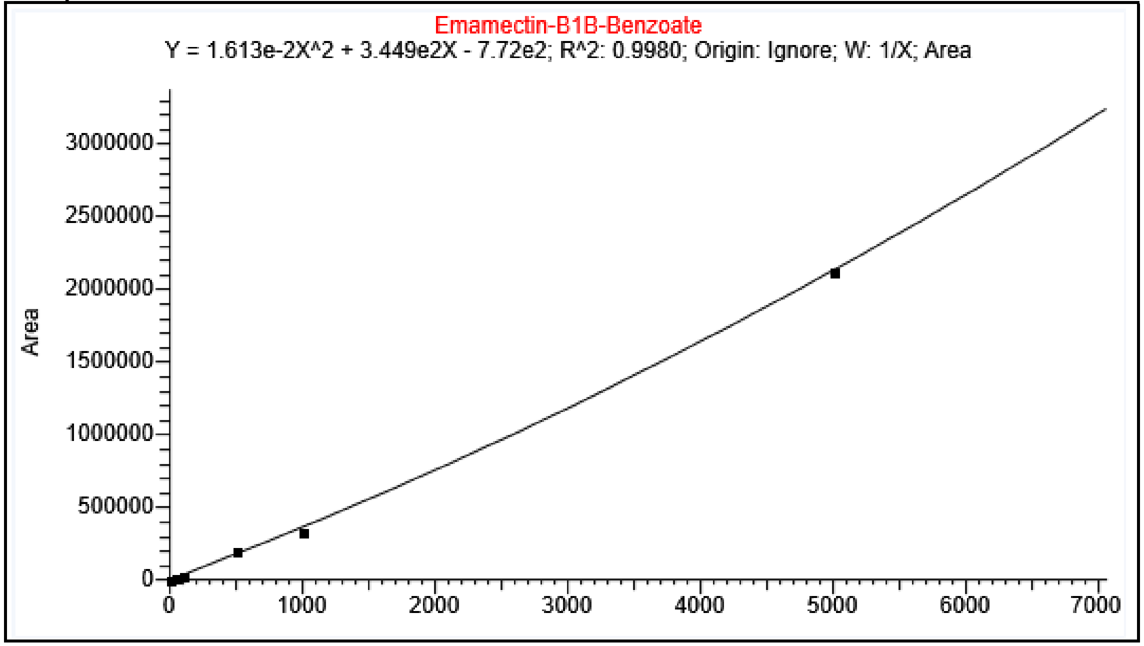

Quadratic
Pass

Level	Std Amount	Std Area	IS Amount	IS Area	Resp factor/ ratio	Calc Amount	Units	% CV	% RSD
3	10	3040			304.028	11.046		N/A	N/A
5	50	14106			282.113	43.045		N/A	N/A
6	100	32296			322.958	95.44		N/A	N/A
8	500	201980			403.96	572.468		N/A	N/A
9	1000	335238			335.238	933.382		N/A	N/A
10	5000	2129558			425.912	5004.822		N/A	N/A

Bordered cell = Manually Integrated; Calibration flags: D=RSD; F=Response factor; R=R Squared; A=Amount.

Compound Calibration Report

Lab Name: Default Laboratory
Instrument: Thermo Scientific Instrument
User: Quantiva_2
Batch: Karisim_Projesi_

Method: Karisim_Projesi__Karisim_Projesi_2020
Karisim_Projesi_2020
Cali File: Karisim_Projesi_.calx

Compound Name: **avermectin b1b**

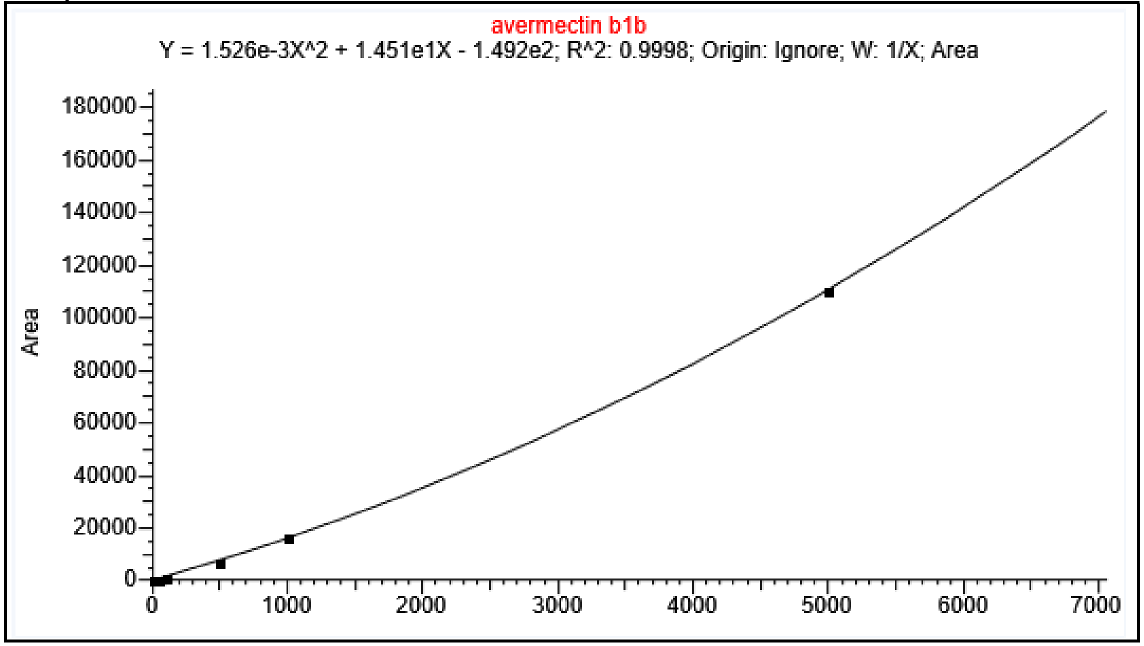

Quadratic
Pass

Level	Std Amount	Std Area	IS Amount	IS Area	Resp factor/ ratio	Calc Amount	Units	% CV	% RSD
3	10	23			2.347	11.889		N/A	N/A
5	50	470			9.391	42.458		N/A	N/A
6	100	1272			12.722	96.981		N/A	N/A
8	500	7218			14.437	483.235		N/A	N/A
9	1000	16345			16.345	1026.089		N/A	N/A
10	5000	110480			22.096	4997.882		N/A	N/A

Bordered cell = Manually Integrated; Calibration flags: D=RSD; F=Response factor; R=R Squared; A=Amount.

Compound Calibration Report

Lab Name: Default Laboratory
Instrument: Thermo Scientific Instrument
User: Quantiva_2
Batch: Karisim_Projesi_

Method: Karisim_Projesi__Karisim_Projesi_2020
Karisim_Projesi_2020
Cali File: Karisim_Projesi_.calx

Compound Name: **EmamectinBenzoateB1A**

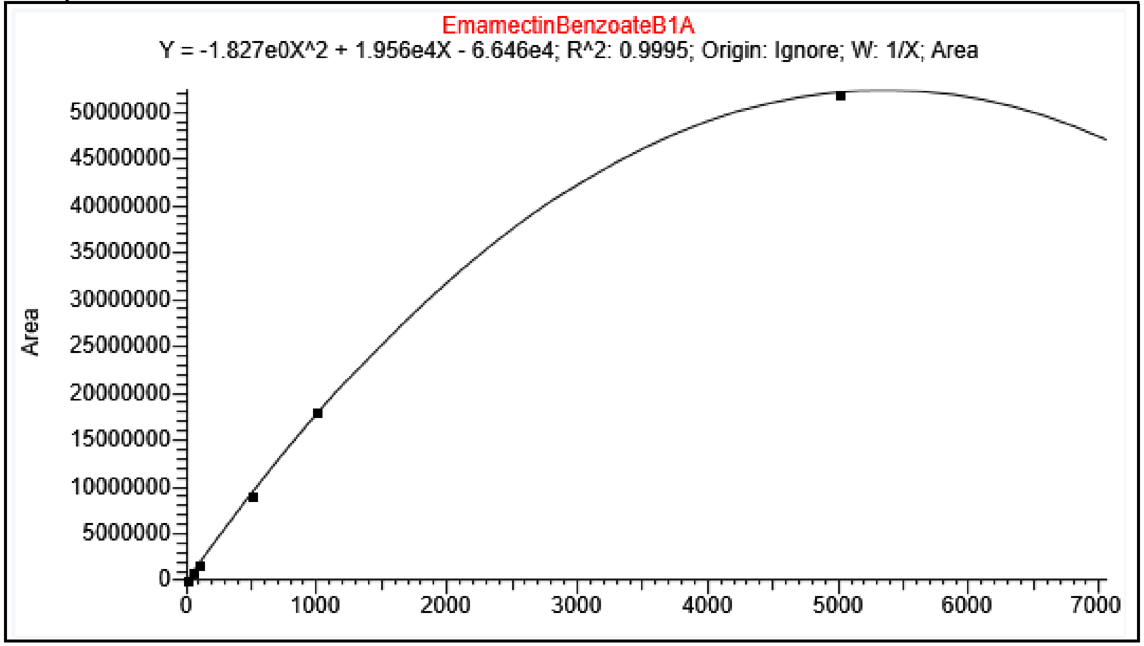

Quadratic
Pass

Level	Std Amount	Std Area	IS Amount	IS Area	Resp factor/ ratio	Calc Amount	Units	% CV	% RSD
3	10	150746			15074.589	11.116		N/A	N/A
5	50	853984			17079.672	47.268		N/A	N/A
6	100	1761693			17616.935	94.298		N/A	N/A
8	500	9044307			18088.615	488.053		N/A	N/A
9	1000	18076865			18076.865	1025.926		N/A	N/A
10	5000	51993469			10398.694	4957.293		N/A	N/A

Bordered cell = Manually Integrated; Calibration flags: D=RSD; F=Response factor; R=R Squared; A=Amount.

Compound Calibration Report

Lab Name: Default Laboratory
Instrument: Thermo Scientific Instrument
User: Quantiva_2
Batch: Karisim_Projesi_

Method: Karisim_Projesi__Karisim_Projesi_2020
Karisim_Projesi_2020
Cali File: Karisim_Projesi_.calx

Compound Name: **Avermectin-B1A**

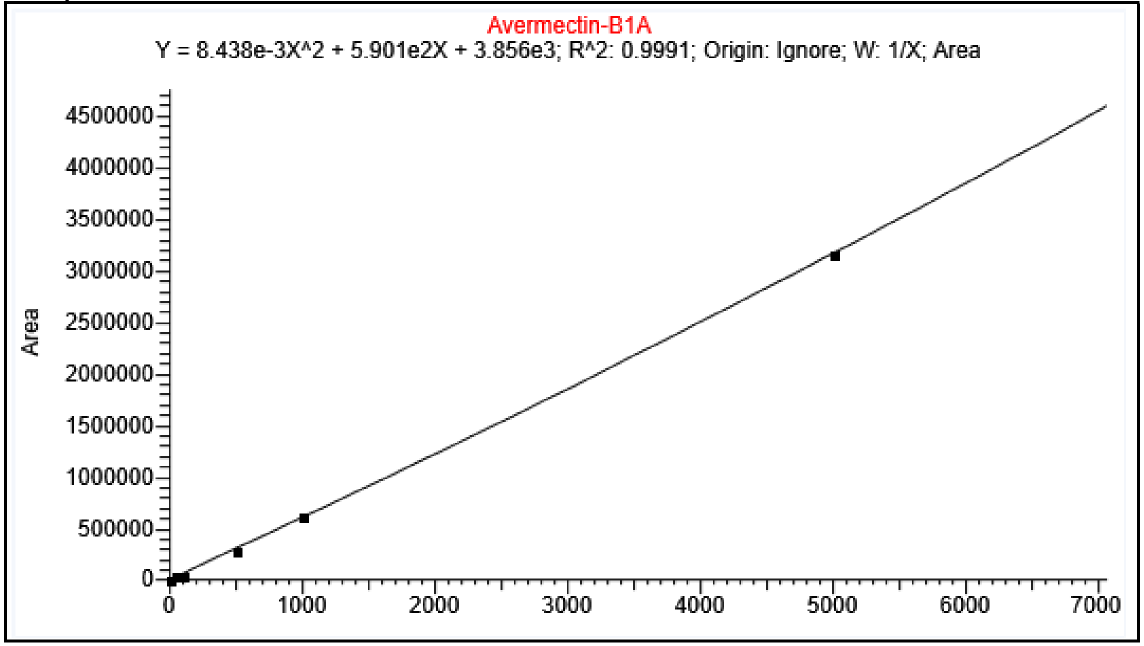

Quadratic
Pass

Level	Std Amount	Std Area	IS Amount	IS Area	Resp factor/ ratio	Calc Amount	Units	% CV	% RSD
3	10	9648			964.815	9.814		N/A	N/A
5	50	39250			784.995	59.927		N/A	N/A
6	100	52656			526.565	82.599		N/A	N/A
8	500	291343			582.686	483.824		N/A	N/A
9	1000	618787			618.787	1026.972		N/A	N/A
10	5000	3163230			632.646	4996.797		N/A	N/A

Bordered cell = Manually Integrated; Calibration flags: D=RSD; F=Response factor; R=R Squared; A=Amount.

Compound Calibration Report

Lab Name: Default Laboratory
Instrument: Thermo Scientific Instrument
User: Quantiva_2
Batch: Karisim_Projesi_

Method: Karisim_Projesi__Karisim_Projesi_2020
Karisim_Projesi_2020
Cali File: Karisim_Projesi_.calx

Compound Name: **Spirodiclofen**

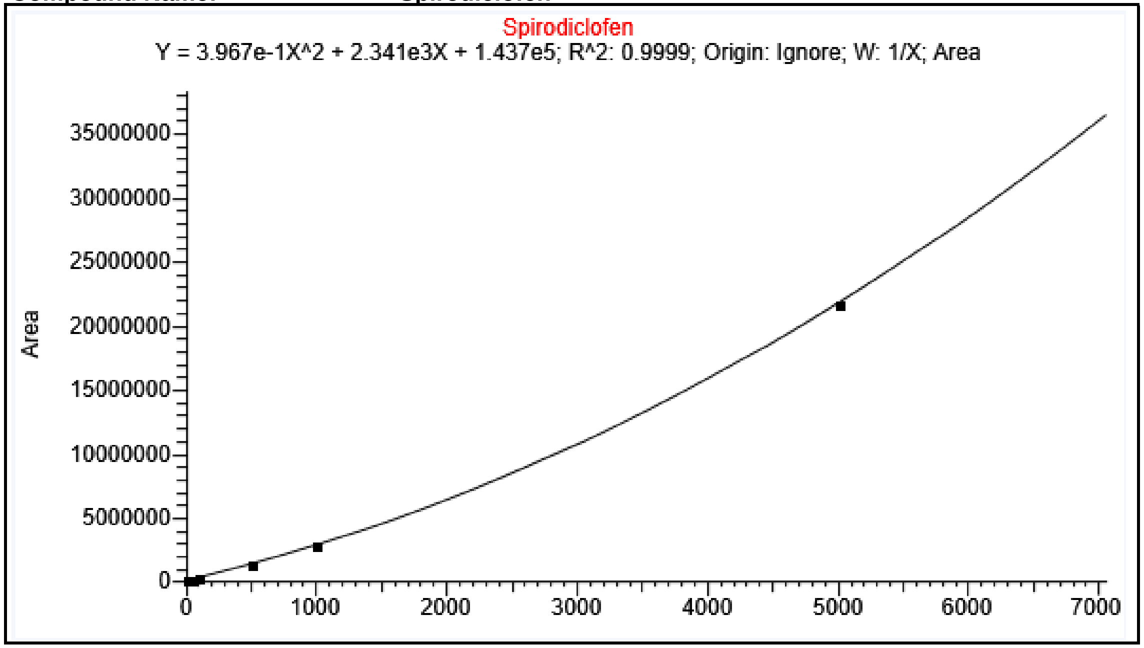

Quadratic
Pass

Level	Std Amount	Std Area	IS Amount	IS Area	Resp factor/ ratio	Calc Amount	Units	% CV	% RSD
3	10	167344			16734.4	10.096		N/A	N/A
5	50	257966			5159.31	48.429		N/A	N/A
6	100	383068			3830.678	100.557		N/A	N/A
8	500	1453472			2906.943	514.659		N/A	N/A
9	1000	2838645			2838.645	986.402		N/A	N/A
10	5000	21769608			4353.922	5000.713		N/A	N/A

Bordered cell = Manually Integrated; Calibration flags: D=RSD; F=Response factor; R=R Squared; A=Amount.
