# Supplementary material for: Pesticide degradation capacity of a novel strain belonging to Serratia sarumanii with its genomic profile
Source: Biodegradation. 2025 Jun 1;36(3):49. doi: 10.1007/s10532-025-10144-2 (PMC12127232; doi:10.1007/s10532-025-10144-2)
Supplement: Supplementary file 1 — Supplementary file1 (ZIP 20243 KB) [file 10532_2025_10144_MOESM1_ESM.zip › Supplementary data5.pdf]

TYPE (STRAIN) GENOME SERVER

JOB SUMMARY

PRINT DATE:	2024-11-04 19:32:35 +0100
JOB ID:	b5a77037-40ef-4f1a-a0db-fce3caa9916e
RESULT PAGE:	https://tygs.dsmz.de/user_results/show?guid=b5a77037-40ef-4f1a-a0db-fce3caa9916e

Table 1: Phylogenies

Publication-ready versions of both the genome-scale GBDP tree and the 16S rRNA gene sequence tree can be customized and exported either in SVG (vector graphic) or PNG format from within the phylogeny viewers in your TYGS result page. For publications the **SVG format is recommended** because it is lossless, always keeps its high resolution and can also be easily converted to other popular formats such as PDF or EPS. Please follow the link provided above!

Table 2: Identification

The below list contains the result of the TYGS species identification routine.

Explanation of remarks that might occur in the below table:

remark [R1]: The TYGS type strain database is automatically updated on an almost daily basis. However, if a particular type strain genome is not available in the TYGS database, this can have several reasons which are detailed in the FAQ. You can request an extended 16S rRNA gene analysis via the 16S tree viewer found in your result page to detect **not yet genome-sequenced** type strains relevant for your study.

remark [R2]: > 70% dDDH value (formula d_4) and (almost) minimal dDDH values for gene-content formulae d_0 and d_6 indicate a potentially unreliable identification result and should thus be checked via the 16S rRNA gene sequence similarity. Such strong deviations can, in principle, be caused by sequence contamination.

remark [R3]: G+C content difference of > 1 % indicates a potentially unreliable identification result because within species G+C content varies no more than 1 %, if computed from genome sequences (PMID: 24505073).

Strain	Conclusion	Identification result	Remark
**'result'**	belongs to known species	*Serratia sarumanii*	

Table 3: Pairwise comparisons of user genomes vs. type-strain genomes

The following table contains the pairwise dDDH values between your user genomes and the selected type-strain genomes. The dDDH values are provided along with their confidence intervals (C.I.) for the three different GBDP formulas:

- formula d_0 (a.k.a. GGDC formula 1): length of all HSPs divided by total genome length
- formula d_4 (a.k.a. GGDC formula 2): sum of all identities found in HSPs divided by overall HSP length
- formula d_6 (a.k.a. GGDC formula 3): sum of all identities found in HSPs divided by total genome length

Note: Formula d_4 is independent of genome length and is thus robust against the use of incomplete draft genomes. For other reasons for preferring formula d_4, see the FAQ.

Query	Subject	d_0	C.I. d_0	d_4	C.I. d_4	d_6	C.I. d_6	Diff. G+C Percent
**'result.fasta'**	*Serratia sarumanii* K-M0706	88.5	[85.0 - 91.2]	79.9	[76.9 - 82.5]	89.8	[87.1 - 92.1]	0.0
**'result.fasta'**	*Serratia nematodiphila* DSM 21420	87.2	[83.7 - 90.1]	78.1	[75.1 - 80.8]	88.5	[85.6 - 90.9]	0.33
**'result.fasta'**	*Serratia marcescens* subsp. *sakuensis* KCTC 42172	83.7	[79.9 - 86.9]	69.2	[66.2 - 72.0]	83.9	[80.7 - 86.7]	0.21
**'result.fasta'**	*Serratia marcescens* ATCC 13880	83.2	[79.3 - 86.4]	69.1	[66.1 - 72.0]	83.5	[80.2 - 86.3]	0.03
**'result.fasta'**	*Serratia bockelmannii* S3 T	78.9	[75.0 - 82.4]	63.4	[60.5 - 66.2]	78.6	[75.2 - 81.7]	0.81
**'result.fasta'**	*Serratia montpellierensis* PsyLou2A	83.3	[79.5 - 86.5]	62.5	[59.7 - 65.4]	82.1	[78.7 - 85.0]	0.29
**'result.fasta'**	*Serratia ureilytica* JCM 16474	81.7	[77.9 - 85.1]	61.9	[59.0 - 64.7]	80.6	[77.2 - 83.6]	0.4
**'result.fasta'**	*Serratia ficaria* NBRC 102596	59.5	[55.8 - 63.0]	34.9	[32.5 - 37.4]	53.3	[50.2 - 56.4]	0.05
**'result.fasta'**	*Serratia entomophila* DSM 12358	62.5	[58.8 - 66.1]	33.6	[31.1 - 36.1]	54.9	[51.8 - 58.0]	0.94
**'result.fasta'**	*Serratia inhibens* S40	45.9	[42.5 - 49.3]	28.3	[25.9 - 30.8]	40.5	[37.6 - 43.6]	4.0
**'result.fasta'**	*Serratia liquefaciens* ATCC 27592	50.1	[46.6 - 53.5]	27.1	[24.8 - 29.6]	42.9	[39.9 - 45.9]	4.53
**'result.fasta'**	*Serratia fonticola* LMG 7882	25.6	[22.3 - 29.3]	24.8	[22.4 - 27.2]	24.3	[21.5 - 27.4]	6.25
**'result.fasta'**	*Dryocola clanedunensis* H11S18	15.5	[12.6 - 19.0]	20.5	[18.2 - 22.9]	15.6	[13.0 - 18.5]	6.01
**'result.fasta'**	*Klebsiella trevisanii* DSM 2688	15.4	[12.5 - 18.9]	20.5	[18.3 - 22.9]	15.5	[13.0 - 18.4]	4.71
**'result.fasta'**	*Enterobacter pasteurii* A-8	16.7	[13.7 - 20.2]	20.5	[18.3 - 23.0]	16.6	[14.0 - 19.5]	3.46

Table 4: Strains in your dataset

Joint dataset of automatically determined closest type strains (if this mode was chosen), manually selected type strains (if selected accordingly) and the provided user strains, if provided (marked in yellow).

Strain	Authority	Other deposits	Synonyms	Base pairs	Percent G+C	No. proteins	Goldstamp	Bioproject accession	Biosample accession	Assembly accession	IMG OID
Serratia inhibens S40	Hennessy et al. 2020	NCIMB 15235; LMG 3146	*Serratia inhibens*	5376 162	55.9	4875		PRJNA491277	SAMN10068376	GCA_003591175	
Serratia entomophila DSM 12358	Grimont et al. 1988	A1; CIP 102919; ATCC 43705	*Serratia entomophila*	5147 822	58.9	4716		PRJNA727746	SAMN19031985	GCA_021462285	
Enterobacter pasteurii A-8	Rahi et al. 2024	and NCTC 13380; CIP 103550; ATCC 23355; CCUG 33777; CECT 5075; DSM 26481; WDCM 82	*Enterobacter pasteurii*	4810 455	56.4	4376		PRJNA937600	SAMN33411377	GCA_028890245	
Serratia marcescens subsp. *sakuensis* KCTC 42172	Ajithkumar et al. 2003	CIP 107489; DSM 17174; JCM 11315; KRED	*Serratia marcescens* subsp. *sakuensis*	5058 045	59.6	4719		PRJNA484649	SAMN09767465	GCA_003428265	
Serratia montpellierensis PsyLou2A	Blackburn et al. 2024	NRRL B-65689; LMG 32817; Lou2A	*Serratia montpellierensis*	5149 841	59.6	4734		PRJNA556397	SAMN12349642	GCA_020858655	
Serratia ureilytica JCM 16474	Bhadra et al. 2005	CCUG 50595; DSM 16952; LMG 22860; NIVA 51	*Serratia ureilytica*	5310 236	59.5	4988		PRJDB10510	SAMD00645019	GCA_039523675	
Serratia sarumanii K-M0706	Klages et al. 2024	DSM 116040; LMG 33111	*Serratia sarumanii*	5185 617	59.9	4769		PRJNA923618	SAMN32727621	GCA_029962605	

Strain	Authority	Other deposits	Synonyms	Base pairs	Percent G+C	No. proteins	Goldstamp	Bioproject accession	Biosample accession	Assembly accession	IMG OID
Serratia marcescens ATCC 13880	Bizio 1823	CFBP 4226; CIP 103235; NRRL B-2544; CCUG 1647; DSM 30121; JCM 1239; NBRC 102204; NCTC 10211; VKM B-1248; HAMBI 1286; LMG 2792	*Serratia marcescens*; *Serratia marcescens* subsp. *marcescens*	5092 451	59.8	4739	Gp0009486	PRJNA59561	SAMN02743269	GCA_000735445	
Serratia nematodiphila DSM 21420	Zhang et al. 2009	CGMCC 1.6853; KCTC 22130; DZ0503SBS1	*Serratia nematodiphila*	5224 920	59.5	4629	Gp0103438	PRJNA257492	SAMN02952129	GCA_000738675	
Serratia ficaria NBRC 102596	Grimont et al. 1981 emend. García-Fraile et al. 2020	4024; CIP 79.23; ATCC 33105; DSM 4569; JCM 1241; NCTC 12148; ICPB 4050; LMG 7881	*Serratia ficaria*	5261 721	59.9	4830	Gp0070998	PRJDB1514	SAMD00046907	GCA_001590885	
Serratia fonticola LMG 7882	Gavini et al. 1979 emend. Kämpfer and Glaeser 2015	11; CIP 78.64; ATCC 29844; CCUG 14186; CCUG 37824; DSM 4576; JCM 1242; NBRC 102597; NCTC 12965; HAMBI 1274; IAM 1242	*Serratia fonticola*	5895 861	53.6	5468	Gp0045178	PRJNA213314	SAMN02471347	GCA_000469035	

Strain	Authority	Other deposits	Synonyms	Base pairs	Percent G+C	No. proteins	Goldstamp	Bioproject accession	Biosample accession	Assembly accession	IMG OID
Serratia liquefaciens ATCC 27592	(Grimes and Hennerty 1931) Bascomb et al. 1971	CIP 103238; CCUG 9285; DSM 4487; JCM 1245; NCTC 12962; NCTC 13756; LMG 7884	*Aerobacter liquefaciens*; *Serratia liquefaciens*	5282 719	55.3	4894	Gp0029592	PRJNA208332	SAMN02604177	GCA_000422085	2563366574
Klebsiella trevisanii DSM 2688	Ferragut et al. 1983	CIP 81.36; ATCC 33558; CUETM 78-120; Gavini K70; HAMBI 1303; LMG 3072	*Klebsiella trevisanii*	6215 359	55.2	5947	Gp0290555	PRJNA500331	SAMN10362885	GCA_004345285	2788499846
Dryocola clanedunensis H11S18	Maddock et al. 2023	CCUG 76181; LMG 32611	*Dryocola clanedunensis*	5220 473	53.9	4755		PRJNA814476	SAMN26554632	GCA_025215155	
Serratia bockelmannii S3 T	Cho et al. 2020	DSM 110152; LMG 31535	*Serratia bockelmannii*	5284 737	59.1	4907		PRJNA554602	SAMN12274630	GCA_008011855	
result.fasta				1016 7833	59.9	9520					

Methods, Results and References

The genome sequence data were uploaded to the Type (Strain) Genome Server (TYGS), a free bioinformatics platform available under https://tygs.dsmz.de, for a whole genome-based taxonomic analysis [1]. The analysis also made use of recently introduced methodological updates and features [2]. Information on nomenclature, synonymy and associated taxonomic literature was provided by TYGS's sister database, the List of Prokaryotic names with Standing in Nomenclature (LPSN, available at https://lpsn.dsmz.de) [2]. The results were provided by the TYGS on 2024-11-03. The TYGS analysis was subdivided into the following steps:

Determination of closely related type strains

Determination of closest type strain genomes was done in two complementary ways: First, all user genomes were compared against all type strain genomes available in the TYGS database via the MASH algorithm, a fast approximation of intergenomic relatedness [3], and, the ten type strains with the smallest MASH distances chosen per user genome. Second, an additional set of ten closely related type strains was determined via the 16S rDNA gene sequences. These were extracted from the user genomes using RNAmmer [4] and each sequence was subsequently BLASTed [5] against the 16S rDNA gene sequence of each of the currently 22010 type strains available in the TYGS database. This was used as a proxy to find the best 50 matching type strains (according to the bitscore) for each user genome and to subsequently calculate precise distances using the Genome BLAST Distance Phylogeny approach (GBDP) under the algorithm 'coverage' and distance formula d_5 [6]. These distances were finally used to determine the 10 closest type strain genomes for each of the user genomes.

Pairwise comparison of genome sequences

For the phylogenomic inference, all pairwise comparisons among the set of genomes were conducted using GBDP and accurate intergenomic distances inferred under the algorithm 'trimming' and distance formula d_5 [6]. 100 distance replicates were calculated each. Digital DDH values and confidence intervals were calculated using the recommended settings of the GGDC 4.0 [2,6].

Phylogenetic inference

The resulting intergenomic distances were used to infer a balanced minimum evolution tree with branch support via FASTME 2.1.6.1 including SPR postprocessing [7]. Branch support was inferred from 100 pseudo-bootstrap replicates each. The trees were rooted at the midpoint [8] and visualized with PhyD3 [9].

Type-based species and subspecies clustering

The type-based species clustering using a 70% dDDH radius around each of the 15 type strains was done as previously described [1]. The resulting groups are shown in Table 1 and 4. Subspecies clustering was done using a 79% dDDH threshold as previously introduced [10].

Results

Type-based species and subspecies clustering

The resulting species and subspecies clusters are listed in Table 4, whereas the taxonomic identification of the query strains is found in Table 1. Briefly, the clustering yielded 13 species clusters and the provided query strains were assigned to 1 of these. Moreover, user strains were located in 1 of 14 subspecies clusters.

Figure caption SSU tree

Figure 1. Tree inferred with FastME 2.1.6.1 [7] from GBDP distances calculated from 16S rDNA gene sequences. The branch lengths are scaled in terms of GBDP distance formula d_5. The numbers above branches are GBDP pseudo-bootstrap support values > 60 % from 100 replications, with an average branch support of 65.1 %. The tree was rooted at the midpoint [8].

Figure caption genome tree

Figure 2. Tree inferred with FastME 2.1.6.1 [7] from GBDP distances calculated from genome sequences. The branch lengths are scaled in terms of GBDP distance formula d_5. The numbers above branches are GBDP pseudo-bootstrap support values > 60 % from 100 replications, with an average branch support of 91.2 %. The tree was rooted at the midpoint [8].

References

[1] Meier-Kolthoff JP, Göker M. TYGS is an automated high-throughput platform for state-of-the-art genome-based taxonomy. Nat. Commun. 2019;10: 2182. DOI: 10.1038/s41467-019-10210-3

[2] Meier-Kolthoff JP, Sardà Carbasse J, Peinado-Olarte RL, Göker M. TYGS and LPSN: a database tandem for fast and reliable genome-based classification and nomenclature of prokaryotes. Nucleic Acid Res. 2022;50: D801–D807. DOI: 10.1093/nar/gkab902

[3] Ondov BD, Treangen TJ, Melsted P, et al. Mash: Fast genome and metagenome distance estimation using MinHash. Genome Biol 2016;17: 1–14. DOI: 10.1186/s13059-016-0997-x

[4] Lagesen K, Hallin P. RNAmmer: consistent and rapid annotation of ribosomal RNA genes. Nucleic Acids Res. Oxford Univ Press; 2007;35: 3100–3108. DOI: 10.1093/nar/gkm160

[5] Camacho C, Coulouris G, Avagyan V, Ma N, Papadopoulos J, Bealer K, et al. BLAST+: architecture and applications. BMC Bioinformatics. 2009;10: 421. DOI: 10.1186/1471-2105-10-421

[6] Meier-Kolthoff JP, Auch AF, Klenk H-P, Göker M. Genome sequence-based species delimitation with confidence intervals and improved distance functions. BMC Bioinformatics. 2013;14: 60. DOI: 10.1186/1471-2105-14-60

[7] Lefort V, Desper R, Gascuel O. FastME 2.0: A comprehensive, accurate, and fast distance-based phylogeny inference program. Mol Biol Evol. 2015;32: 2798–2800. DOI: 10.1093/molbev/msv150

[8] Farris JS. Estimating phylogenetic trees from distance matrices. Am Nat. 1972;106: 645–667.

[9] Kreft L, Botzki A, Coppens F, Vandepoele K, Van Bel M. PhyD3: A phylogenetic tree viewer with extended phyloXML support for functional genomics data visualization. Bioinformatics. 2017;33: 2946–2947. DOI: 10.1093/bioinformatics/btx324

[10] Meier-Kolthoff JP, Hahnke RL, Petersen J, Scheuner C, Michael V, Fiebig A, et al. Complete genome sequence of DSM 30083^T, the type strain (U5/41^T) of *Escherichia coli*, and a proposal for delineating subspecies in microbial taxonomy. Stand Genomic Sci. 2014;9: 2. DOI: 10.1186/1944-3277-9-2
