# Supplementary material for: Pesticide degradation capacity of a novel strain belonging to Serratia sarumanii with its genomic profile
Source: Biodegradation. 2025 Jun 1;36(3):49. doi: 10.1007/s10532-025-10144-2 (PMC12127232; doi:10.1007/s10532-025-10144-2)
Supplement: Supplementary file 1 — Supplementary file1 (ZIP 20243 KB) [file 10532_2025_10144_MOESM1_ESM.zip › Supplementary data6.pdf]

Sequence query

Please paste in your sequence to query against the database. Query sequences will be checked first for an exact match against the chosen (or all) loci - they do not need to be trimmed. The nearest partial matches will be identified if an exact match is not found. You can query using either DNA or peptide sequences.

Please select locus/scheme

Ribosomal MLST

Order results by

best match

Enter query sequence (single or multiple contigs up to whole genome in size)

Alternatively upload FASTA file

Select FASTA file:

Click to select or drag and drop...

Action

RESET SUBMIT

Predicted taxa

Rank	Taxon	Support	Taxonomy
SPECIES	Serratia sarumanii	81%	*Pseudomonadota* > *Gammaproteobacteria* > *Enterobacterales* > *Yersiniaceae* > *Serratia* > *Serratia sarumanii*
SPECIES	Serratia nematodiphila	10%	*Pseudomonadota* > *Gammaproteobacteria* > *Enterobacterales* > *Yersiniaceae* > *Serratia* > *Serratia nematodiphila*

Uploaded file: 23480194.3.contigs.fa

51 exact matches found.

Locus	Allele	Length	Contig	Start position	End position	Linked data values	Flags	Comments
BACT000001	96346	1674	G3_FDMS202324982-1a_1_(paired)_contig_112	39747	41420			
BACT000002	1633	726	G3_FDMS202324982-1a_1_(paired)_contig_154	5648	6373	rMLST genome database **species:** *Serratia sp.* [n=6]; *Serratia sarumanii* [n=3]		
BACT000003	1475	699	G3_FDMS202324982-1a_1_(paired)_contig_192	4700	5398	rMLST genome database **species:** *Serratia sp.* [n=8]; *Serratia sarumanii* [n=4]		
BACT000004	1522	621	G3_FDMS202324982-1a_1_(paired)_contig_192	12766	13386	rMLST genome database **species:** *Serratia ureilytica* [n=149]; *Serratia sp.* [n=19]; *Serratia sarumanii* [n=4]; *Serratia nevei* [n=2]		
BACT000005	1445	501	G3_FDMS202324982-1a_1_(paired)_contig_192	9211	9711	rMLST genome database **species:** *Serratia marcescens* [n=159]; *Serratia ureilytica* [n=149]; *Serratia sp.* [n=27]; *Serratia nematodiphila* [n=6]; *Serratia sarumanii* [n=4]; *Serratia bockelmannii* [n=1]		

BACT000006	1385	399	G3_FDMS202324982-1a_1_(paired)_contig_405	1069	1467	rMLST genome database **species:** *Serratia nevei* [n=16]; *Serratia sp.* [n=16]; *Serratia sarumanii* [n=4]; *Serratia surfactantfaciens* [n=4]
BACT000007	1411	471	G3_FDMS202324982-1a_1_(paired)_contig_287	2354	2824	rMLST genome database **species:** *Serratia marcescens* [n=170]; *Serratia ureilytica* [n=158]; *Serratia nevei* [n=108]; *Serratia sp.* [n=63]; *Serratia bockelmannii* [n=41]; *Serratia nematodiphila* [n=6]; *Serratia surfactantfaciens* [n=4]; *Serratia montpellierensis* [n=2]; *Serratia sarumanii* [n=2]
BACT000008	1339	393	G3_FDMS202324982-1a_1_(paired)_contig_192	7933	8325	rMLST genome database **species:** *Serratia ureilytica* [n=119]; *Serratia nevei* [n=96]; *Serratia sp.* [n=48]; *Serratia bockelmannii* [n=41]; *Serratia nematodiphila* [n=5]; *Serratia sarumanii* [n=4]; *Serratia montpellierensis* [n=2]; *Serratia marcescens* [n=1]
BACT000009	1408	393	G3_FDMS202324982-1a_1_(paired)_contig_505	92	484	rMLST genome database **species:** *Serratia sp.* [n=6]; *Serratia sarumanii* [n=4]
BACT000010	1340	312	G3_FDMS202324982-1a_1_(paired)_contig_192	1311	1622	rMLST genome database **species:** *Serratia sp.* [n=12]; *Serratia ureilytica* [n=12]; *Serratia marcescens* [n=10]; *Serratia sarumanii* [n=4]; *Serratia surfactantfaciens* [n=1]
BACT000011	1310	390	G3_FDMS202324982-1a_1_(paired)_contig_192	12346	12735	rMLST genome database **species:** *Serratia sp.* [n=8]; *Serratia sarumanii* [n=3]
BACT000012	3830	375	G3_FDMS202324982-1a_1_(paired)_contig_287	2921	3295	rMLST genome database **species:** *Serratia sp.* [n=4]; *Serratia ureilytica* [n=4]; *Serratia sarumanii* [n=1]
BACT000013	1288	357	G3_FDMS202324982-1a_1_(paired)_contig_192	11973	12329	rMLST genome database **species:** *Serratia marcescens* [n=169]; *Serratia ureilytica* [n=152]; *Serratia nevei* [n=112]; *Serratia sp.* [n=58]; *Serratia bockelmannii* [n=20]; *Serratia sarumanii* [n=3]; *Serratia montpellierensis* [n=2]

BACT000014	1260	306	G3_FDMS202324982-1a_1_(paired)_contig_192	7597	7902	rMLST genome database **species:** *Serratia marcescens* [n=171]; *Serratia ureilytica* [n=156]; *Serratia sp.* [n=51]; *Serratia bockelmannii* [n=41]; *Serratia nematodiphila* [n=6]; *Serratia sarumanii* [n=4]; *Streptomyces sp.* [n=1]
BACT000015	113115	270	G3_FDMS202324982-1a_1_(paired)_contig_375	14338	14607	
BACT000016	1318	249	G3_FDMS202324982-1a_1_(paired)_contig_340	8915	9163	rMLST genome database **species:** *Serratia sp.* [n=6]; *Serratia sarumanii* [n=2]
BACT000018	1261	228	G3_FDMS202324982-1a_1_(paired)_contig_405	511	738	rMLST genome database **species:** *Serratia ureilytica* [n=162]; *Serratia marcescens* [n=131]; *Serratia nevei* [n=112]; *Serratia sp.* [n=67]; *Serratia bockelmannii* [n=41]; *Serratia nematodiphila* [n=6]; *Serratia surfactantfaciens* [n=4]; *Serratia sarumanii* [n=3]; *Serratia montpellierensis* [n=2]
BACT000019	1208	279	G3_FDMS202324982-1a_1_(paired)_contig_192	4057	4335	rMLST genome database **species:** *Serratia marcescens* [n=171]<br>*Serratia ureilytica* [n=162]<br>*Serratia nevei* [n=112]<br>*Serratia sp.* [n=83]<br>*Serratia liquefaciens* [n=80]<br>*Serratia entomophila* [n=68]<br>*Serratia bockelmannii* [n=41]<br>*Serratia ficaria* [n=25]<br>*Serratia nematodiphila* [n=6]<br>*Serratia odorifera* [n=6]<br>*Serratia sarumanii* [n=4]<br>*Serratia surfactantfaciens* [n=4]<br>*Rahnella inusitata* [n=3]<br>*Serratia inhibens* [n=3]<br>*Chania multitudinisentens* [n=2]<br>*Serratia montpellierensis* [n=2]<br>*Serratia oryzae* [n=2]<br>*Rahnella rivi* [n=1]

						Rahnella sikkimica [n=1] *Rahnella sp.* [n=1]
BACT000020	5190	264	G3_FDMS202324982-1a_1_(paired)_contig_213	3013	3276	rMLST genome database **species:** *Serratia sp.* [n=4]; *Serratia sarumanii* [n=3]
BACT000021	1299	216	G3_FDMS202324982-1a_1_(paired)_contig_387	1879	2094	rMLST genome database **species:** *Serratia marcescens* [n=171]; *Serratia ureilytica* [n=117]; *Serratia nevei* [n=88]; *Serratia sp.* [n=54]; *Serratia bockelmannii* [n=41]; *Serratia nematodiphila* [n=5]; *Serratia surfactantfaciens* [n=4]; *Serratia sarumanii* [n=3]; *Serratia montpellierensis* [n=2]
BACT000030	1607	705	G3_FDMS202324982-1a_1_(paired)_contig_29	3704	4408	rMLST genome database **species:** *Serratia sp.* [n=5]; *Serratia sarumanii* [n=3]
BACT000031	6412	825	G3_FDMS202324982-1a_1_(paired)_contig_192	3218	4042	rMLST genome database **species:** *Serratia marcescens* [n=141]; *Serratia sp.* [n=10]; *Serratia nevei* [n=7]; *Serratia nematodiphila* [n=2]; *Serratia sarumanii* [n=1]; *Serratia ureilytica* [n=1]
BACT000032	9339	630	G3_FDMS202324982-1a_1_(paired)_contig_192	1655	2284	
BACT000033	4309	606	G3_FDMS202324982-1a_1_(paired)_contig_192	2295	2900	rMLST genome database **species:** *Serratia ureilytica* [n=110]; *Serratia sp.* [n=22]; *Serratia nevei* [n=3]; *Serratia sarumanii* [n=3]; *Serratia bockelmannii* [n=1]
BACT000036	1399	366	G3_FDMS202324982-1a_1_(paired)_contig_29	2439	2804	rMLST genome database **species:** *Serratia sp.* [n=7]; *Serratia sarumanii* [n=4]
BACT000038	4165	453	G3_FDMS202324982-1a_1_(paired)_contig_405	19	471	rMLST genome database **species:** *Serratia marcescens* [n=158]; *Serratia sp.* [n=16]; *Serratia nevei* [n=6]; *Serratia nematodiphila* [n=5]; *Serratia sarumanii* [n=4]
BACT000039	1418	498	G3_FDMS202324982-1a_1_(paired)_contig_29	2868	3365	rMLST genome database **species:** *Serratia marcescens* [n=169]; *Serratia ureilytica* [n=155]; *Serratia sp.* [n=55]; *Serratia bockelmannii* [n=39]; *Serratia nevei* [n=32]; *Serratia nematodiphila* [n=6]; *Serratia*

						sarumanii [n=4]; *Serratia surfactantfaciens* [n=4]; *Serratia montpellierensis* [n=2]
BACT000040	4058	429	G3_FDMS202324982-1a_1_(paired)_contig_29	4412	4840	rMLST genome database **species:** *Serratia bockelmannii* [n=13]; *Serratia sp.* [n=7]; *Serratia montpellierensis* [n=2]; *Serratia sarumanii* [n=2]
BACT000042	1397	429	G3_FDMS202324982-1a_1_(paired)_contig_505	500	928	rMLST genome database **species:** *Serratia marcescens* [n=169]; *Serratia ureilytica* [n=141]; *Serratia nevei* [n=109]; *Serratia sp.* [n=64]; *Serratia bockelmannii* [n=40]; *Serratia nematodiphila* [n=6]; *Serratia sarumanii* [n=4]; *Serratia surfactantfaciens* [n=4]; *Serratia montpellierensis* [n=2]
BACT000043	1290	372	G3_FDMS202324982-1a_1_(paired)_contig_192	6402	6773	rMLST genome database **species:** *Serratia marcescens* [n=168]; *Serratia ureilytica* [n=162]; *Serratia nevei* [n=104]; *Serratia sp.* [n=52]; *Serratia nematodiphila* [n=6]; *Serratia sarumanii* [n=4]; *Serratia surfactantfaciens* [n=1]
BACT000044	1422	435	G3_FDMS202324982-1a_1_(paired)_contig_192	9901	10335	rMLST genome database **species:** *Serratia marcescens* [n=168]; *Serratia ureilytica* [n=152]; *Serratia nevei* [n=111]; *Serratia sp.* [n=55]; *Serratia nematodiphila* [n=6]; *Serratia sarumanii* [n=4]; *Serratia surfactantfaciens* [n=4]
BACT000045	1311	411	G3_FDMS202324982-1a_1_(paired)_contig_192	5411	5821	rMLST genome database **species:** *Serratia marcescens* [n=169]; *Serratia ureilytica* [n=162]; *Serratia nevei* [n=112]; *Serratia sp.* [n=68]; *Serratia bockelmannii* [n=41]; *Serratia nematodiphila* [n=6]; *Serratia sarumanii* [n=4]; *Serratia surfactantfaciens* [n=4]; *Serratia montpellierensis* [n=2]
BACT000046	1354	390	G3_FDMS202324982-1a_1_(paired)_contig_192	14442	14831	rMLST genome database **species:** *Serratia marcescens* [n=168]; *Serratia ureilytica* [n=158]; *Serratia nevei* [n=112]; *Serratia sp.* [n=51]; *Serratia nematodiphila* [n=6];

						Serratia sarumanii [n=3]; *Serratia bockelmannii* [n=2]; *Serratia montpellierensis* [n=2]
BACT000047	1314	354	G3_FDMS202324982-1a_1_(paired)_contig_192	8843	9196	rMLST genome database **species:** *Serratia marcescens* [n=171]; *Serratia ureilytica* [n=156]; *Serratia sp.* [n=50]; *Serratia nevei* [n=38]; *Serratia nematodiphila* [n=6]; *Serratia sarumanii* [n=4]; *Serratia surfactantfaciens* [n=4]; *Serratia montpellierensis* [n=2]
BACT000048	1399	357	G3_FDMS202324982-1a_1_(paired)_contig_340	7123	7479	rMLST genome database **species:** *Serratia bockelmannii* [n=41]; *Serratia sp.* [n=20]; *Serratia sarumanii* [n=4]
BACT000050	1311	312	G3_FDMS202324982-1a_1_(paired)_contig_328	6763	7074	rMLST genome database **species:** *Serratia ureilytica* [n=150]; *Serratia sp.* [n=29]; *Serratia sarumanii* [n=4]
BACT000051	1329	333	G3_FDMS202324982-1a_1_(paired)_contig_192	4350	4682	rMLST genome database **species:** *Serratia ureilytica* [n=162]; *Serratia marcescens* [n=154]; *Serratia nevei* [n=112]; *Serratia entomophila* [n=68]; *Serratia sp.* [n=54]; *Serratia bockelmannii* [n=24]; *Serratia nematodiphila* [n=6]; *Serratia sarumanii* [n=4]; *Serratia surfactantfaciens* [n=4]; *Serratia montpellierensis* [n=2]
BACT000052	1286	303	G3_FDMS202324982-1a_1_(paired)_contig_192	2897	3199	rMLST genome database **species:** *Serratia bockelmannii* [n=41]; *Serratia sp.* [n=19]; *Serratia marcescens* [n=9]; *Serratia nevei* [n=6]; *Serratia montpellierensis* [n=2]; *Serratia sarumanii* [n=2]
BACT000056	1343	258	G3_FDMS202324982-1a_1_(paired)_contig_328	7092	7349	rMLST genome database **species:** *Serratia marcescens* [n=167]<br>*Serratia ureilytica* [n=162]<br>*Serratia nevei* [n=105]<br>*Serratia sp.* [n=69]<br>*Serratia entomophila* [n=68]<br>*Serratia bockelmannii* [n=41]

						Serratia ficaria [n=24]<br>*Serratia nematodiphila* [n=6]<br>*Serratia sarumanii* [n=4]<br>*Serratia surfactantfaciens* [n=4]<br>*Serratia montpellierensis* [n=2]
BACT000057	1174	237	G3_FDMS202324982-1a_1_(paired)_contig_79	730	966	rMLST genome database **species:** *Serratia marcescens* [n=171]; *Serratia ureilytica* [n=161]; *Serratia nevei* [n=110]; *Serratia sp.* [n=68]; *Serratia bockelmannii* [n=40]; *Serratia nematodiphila* [n=6]; *Serratia sarumanii* [n=4]; *Serratia surfactantfaciens* [n=4]; *Serratia montpellierensis* [n=2]
BACT000058	71545	192	G3_FDMS202324982-1a_1_(paired)_contig_192	5821	6012	
BACT000059	1009	180	G3_FDMS202324982-1a_1_(paired)_contig_192	9718	9897	rMLST genome database **species:** *Serratia marcescens* [n=170]; *Serratia ureilytica* [n=161]; *Serratia sp.* [n=62]; *Serratia bockelmannii* [n=41]; *Serratia nevei* [n=38]; *Serratia nematodiphila* [n=6]; *Serratia sarumanii* [n=4]; *Serratia surfactantfaciens* [n=4]; *Serratia montpellierensis* [n=2]
BACT000060	2124	255	G3_FDMS202324982-1a_1_(paired)_contig_182	28281	28535	rMLST genome database **species:** *Serratia sp.* [n=5]; *Serratia nematodiphila* [n=2]
BACT000060	5819	216	G3_FDMS202324982-1a_1_(paired)_contig_527	2215	2430	rMLST genome database **species:** *Serratia marcescens* [n=170]; *Serratia ureilytica* [n=140]; *Serratia bockelmannii* [n=41]; *Serratia sp.* [n=41]; *Serratia nevei* [n=29]; *Serratia sarumanii* [n=4]; *Serratia surfactantfaciens* [n=4]; *Serratia montpellierensis* [n=2]; *Serratia nematodiphila* [n=2]
BACT000061	1208	168	G3_FDMS202324982-1a_1_(paired)_contig_82	1183	1350	rMLST genome database **species:** *Serratia marcescens* [n=171]; *Serratia ureilytica* [n=156]; *Serratia nevei* [n=102]; *Serratia sp.* [n=55]; *Serratia nematodiphila* [n=6]; *Serratia bockelmannii* [n=5]; *Serratia*

						sarumanii [n=4]; *Serratia surfactantfaciens* [n=3]; *Serratia montpellierensis* [n=2]
BACT000062	1250	168	G3_FDMS202324982-1a_1_(paired)_contig_79	551	718	rMLST genome database **species:** *Serratia marcescens* [n=169]; *Serratia ureilytica* [n=160]; *Serratia sp.* [n=67]; *Serratia nevei* [n=62]; *Serratia bockelmannii* [n=40]; *Serratia nematodiphila* [n=6]; *Serratia sarumanii* [n=4]; *Serratia surfactantfaciens* [n=4]; *Serratia montpellierensis* [n=2]
BACT000063	1129	141	G3_FDMS202324982-1a_1_(paired)_contig_463	2036	2176	rMLST genome database **species:** *Serratia marcescens* [n=170]; *Serratia ureilytica* [n=161]; *Serratia nevei* [n=111]; *Serratia sp.* [n=67]; *Serratia bockelmannii* [n=40]; *Serratia nematodiphila* [n=6]; *Serratia sarumanii* [n=4]; *Serratia surfactantfaciens* [n=4]; *Serratia montpellierensis* [n=2]
BACT000064	1214	198	G3_FDMS202324982-1a_1_(paired)_contig_566	163	360	rMLST genome database **species:** *Serratia marcescens* [n=163]; *Serratia ureilytica* [n=157]; *Serratia nevei* [n=109]; *Serratia sp.* [n=66]; *Serratia bockelmannii* [n=41]; *Serratia nematodiphila* [n=6]; *Serratia sarumanii* [n=4]; *Serratia montpellierensis* [n=2]
BACT000065	5225	141	G3_FDMS202324982-1a_1_(paired)_contig_181	43602	43742	rMLST genome database **species:** *Serratia ureilytica* [n=154]; *Serratia nevei* [n=111]; *Serratia sp.* [n=45]; *Serratia sarumanii* [n=4]
BACT000065	532	117	G3_FDMS202324982-1a_1_(paired)_contig_192	11708	11824	rMLST genome database **species:** *Serratia marcescens* [n=171]<br>*Serratia ureilytica* [n=162]<br>*Serratia nevei* [n=111]<br>*Serratia sp.* [n=98]<br>*Serratia liquefaciens* [n=83]<br>*Serratia fonticola* [n=70]<br>*Serratia entomophila* [n=68]<br>*Serratia quinivorans* [n=44]<br>*Serratia bockelmannii* [n=39]

						Serratia proteamaculans [n=32]<br>*Serratia plymuthica* [n=30]<br>*Serratia ficaria* [n=25]<br>*Serratia grimesii* [n=13]<br>*Serratia rubidaea* [n=8]<br>*Serratia nematodiphila* [n=6]<br>*Serratia odorifera* [n=6]<br>*Serratia sarumanii* [n=4]<br>*Serratia surfactantfaciens* [n=4]<br>*Serratia inhibens* [n=3]<br>*Serratia montpellierensis* [n=2]<br>*Serratia rhizosphaerae* [n=2]<br>*Serratia silvae* [n=1]
BACT000065	13679	141	G3_FDMS202324982-1a_1_(paired)_contig_182	28128	28268	rMLST genome database **species:** *Serratia nematodiphila* [n=6]; *Serratia sp.* [n=5]; *Serratia ureilytica* [n=5]

Only exact matches are shown above. If a locus does not have an exact match, try querying specifically against that locus to find the closest match.

Text Excel

Ribosomal MLST

Matching profiles

Closest profile: rST-19799 and 2 others

Fields: **genus:** Serratia; **species:** Serratia sp.

Mismatches: 10

Loci matched: 43/53 (81.1%)

Similar profiles (determined by classification schemes)

Experimental schemes are subject to change and are not a stable part of the nomenclature.

rST-19799 belongs to the following clusters:

Classification scheme	Clustering method	Mismatch threshold	Status	Group	Profiles	Isolates	
Bact_rmlstc_20 ⓘ	Single-linkage	20	experimental	1041	531	rMLST genome database	501
Bact_rmlstc_10 ⓘ	Single-linkage	10	experimental	2696	121	rMLST genome database	12

Contact

Get in touch with us if you have any comments or suggestions concerning the website and the databases.

Cite us

Please cite Jolley *et al.* 2018 Wellcome Open Res 3:124 if you use data or analysis from PubMLST in your publications.

Follow

Supported by

wellcome

Disclaimer & Privacy | Cookies | Terms & Conditions

Website by Manta Ray Media
