# Supplementary material for: Pesticide degradation capacity of a novel strain belonging to Serratia sarumanii with its genomic profile
Source: Biodegradation. 2025 Jun 1;36(3):49. doi: 10.1007/s10532-025-10144-2 (PMC12127232; doi:10.1007/s10532-025-10144-2)
Supplement: Supplementary file 1 — Supplementary file1 (ZIP 20243 KB) [file 10532_2025_10144_MOESM1_ESM.zip › Supplementary data7.pdf]

Profile information for ST-66 (MLST)

ST	adk	fumC	gyrB	icd	mdh	recA
66	129	182	136	178	131	195

sender: Melissa Jansen van Rensburg, UKHSA
curator: Kayo Bianco, Fiocruz
update history: 1 update show details
date entered: 2024-01-23
datestamp: 2024-01-23

Tools

Export: **Profiles** **Sequences**

Contact

Get in touch with us if you have any comments or suggestions concerning the website and the databases.

Cite us

Please cite Jolley *et al.* 2018 Wellcome Open Res 3:124 if you use data or analysis from PubMLST in your publications.

Follow

Supported by

Disclaimer & Privacy | Cookies | Terms & Conditions
Website by Manta Ray Media
