# Supplementary material for: Pesticide degradation capacity of a novel strain belonging to Serratia sarumanii with its genomic profile
Source: Biodegradation. 2025 Jun 1;36(3):49. doi: 10.1007/s10532-025-10144-2 (PMC12127232; doi:10.1007/s10532-025-10144-2)
Supplement: Supplementary file 1 — Supplementary file1 (ZIP 20243 KB) [file 10532_2025_10144_MOESM1_ESM.zip › Supplementary data8.pdf]

KEGG Pathway Maps - Serratia sarumanii

- **Metabolism**
  - Global and overview maps
  - Carbohydrate metabolism
  - Energy metabolism
  - Lipid metabolism
  - Nucleotide metabolism
  - Amino acid metabolism
  - Metabolism of other amino acids
  - Glycan biosynthesis and metabolism
  - Metabolism of cofactors and vitamins
  - Metabolism of terpenoids and polyketides
  - Biosynthesis of other secondary metabolites
  - Xenobiotics biodegradation and metabolism
    - 00362 Benzoate degradation
    - 00627 Aminobenzoate degradation
    - 00364 Fluorobenzoate degradation
    - 00625 Chloroalkane and chloroalkene degradation
    - 00361 Chlorocyclohexane and chlorobenzene degradation
    - 00623 Toluene degradation
    - 00622 Xylene degradation
    - 00633 Nitrotoluene degradation
    - 00642 Ethylbenzene degradation
    - 00643 Styrene degradation
    - 00791 Atrazine degradation
    - 00930 Caprolactam degradation
    - 00363 Bisphenol degradation
    - 00621 Dioxin degradation
    - 00626 Naphthalene degradation
    - 00624 Polycyclic aromatic hydrocarbon degradation
    - 00365 Furfural degradation
    - 00984 Steroid degradation
    - 00980 Metabolism of xenobiotics by cytochrome P450
    - 00982 Drug metabolism - cytochrome P450
    - 00983 Drug metabolism - other enzymes
  - Chemical structure transformation maps
- **Genetic Information Processing**
- **Environmental Information Processing**
- **Cellular Processes**
- **Organismal Systems**
- **Human Diseases**
- **Drug Development**

Last updated: October 29, 2024
