# Supplementary material for: Pesticide degradation capacity of a novel strain belonging to Serratia sarumanii with its genomic profile
Source: Biodegradation. 2025 Jun 1;36(3):49. doi: 10.1007/s10532-025-10144-2 (PMC12127232; doi:10.1007/s10532-025-10144-2)
Supplement: Supplementary file 1 — Supplementary file1 (ZIP 20243 KB) [file 10532_2025_10144_MOESM1_ESM.zip › Supplementary data9.pdf]

results - Region 1 - betalactone

Location: 100,426 - 126,094 nt. (total: 25,669 nt) Show pHMM detection rules used

Download region SVG Download region GenBank file

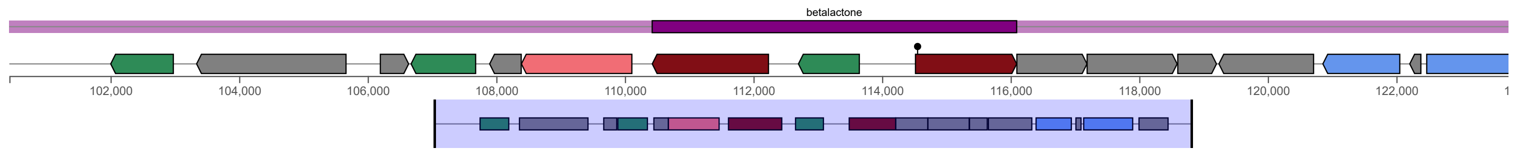

Legend:

core biosynthetic genes | additional biosynthetic genes | transport-related genes | regulatory genes | other genes | resistance | binding site

reset view | zoom to selection

Gene overview | MIBiG comparison | ClusterBlast | KnownClusterBlast | SubClusterBlast | TFBS Finder | Pfam domains | TIGRFAM domains

Similar gene clusters

Analysis type: Protocluster to Region

Query

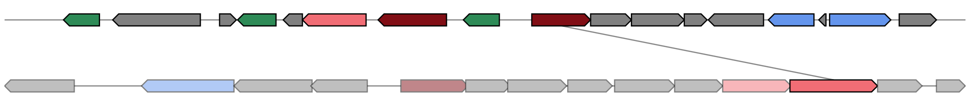

Reference: BGC0001859: 6609-19144

Reference	betalactone	Similarity score	Type	Compound(s)	Organism
BGC0001859		0.20	Other (Phosphonate)	fosfomycin	Pseudomonas syringae
BGC0000595		0.16	RiPP	SCO-2138	Streptomyces coelicolor A3(2)
BGC0000596		0.16	RiPP	SLI-2138	Streptomyces lividans TK24
BGC0000904		0.16	Other	FR-900098	Streptomyces rubellomurinus
BGC0002274		0.15	NRP	aspulvinone H, aspulvinone B1	Aspergillus terreus NIH2624
BGC0002075		0.14	NRP, Alkaloid	pyreudione A, pyreudione B, pyreudione C, pyreudione D, pyreudione E	Pseudomonas fluorescens
BGC0001135		0.14	NRP	bicornutin A1, bicornutin A2	Xenorhabdus budapestensis
BGC0002158		0.13	NRP, Polyketide	tenuazonic acid	Pyricularia oryzae 70-15
BGC0002351		0.13	NRP	thanafactin A	Pseudomonas sp. SHC52
BGC0001441		0.12	Other	belactosin A, belactosin C	Streptomyces sp.

Gene details

Select a gene to view the details available for it

TFBS Finder

TFBS definitions

Regulator	Description
BldD	Development and antibiotic global regulator
LexA	Repressor of DNA damage response
CelR	Cellobiose uptake repressor
AfsQ1	Two-component system AfsQ1-Q2, activator of antibiotic production

If you have found antiSMASH useful, please cite us.

antiSMASH version 7.1.0

Download About Help Contact

Select genomic region:

Overview 1.1 1.2 1.3 1.4 1.5 1.6 1.7 1.8 1.9 1.10 1.11 1.12 1.13 1.14 1.15 1.16 1.17 1.18 1.19 1.20 1.21 1.22 1.23 1.24 1.25

results - Region 2 - betalactone

Location: 140,931 - 166,600 nt. (total: 25,670 nt) Show pHMM detection rules used

Download region SVG Download region GenBank file

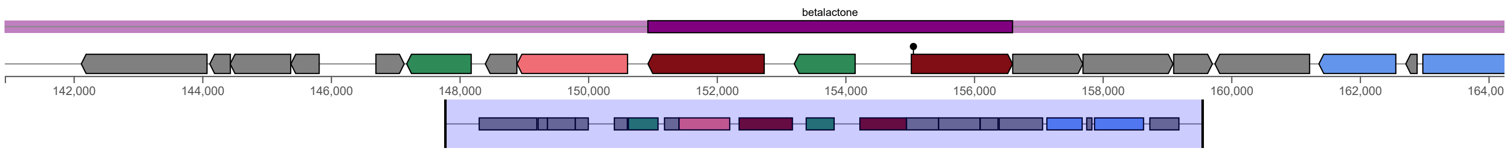

Legend:

core biosynthetic genes additional biosynthetic genes transport-related genes regulatory genes other genes resistance binding site

reset view zoom to selection

Gene overview MIBiG comparison ClusterBlast KnownClusterBlast SubClusterBlast TFBS Finder Pfam domains TIGRFAM domains

Similar gene clusters

Analysis type: Protocluster to Region

Query

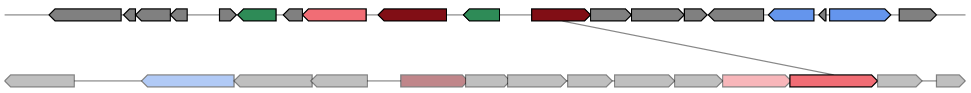

Reference: BGC0001859: 6609-19144

Reference	betalactone	Similarity score	Type	Compound(s)	Organism
BGC0001859		0.20	Other (Phosphonate)	fosfomycin	Pseudomonas syringae
BGC0000904		0.16	Other	FR-900098	Streptomyces rubellomurinus
BGC0002274		0.15	NRP	aspulvinone H, aspulvinone B1	Aspergillus terreus NIH2624
BGC0000595		0.14	RiPP	SCO-2138	Streptomyces coelicolor A3(2)
BGC0000596		0.14	RiPP	SLI-2138	Streptomyces lividans TK24
BGC0001168		0.14	NRP	livipeptin	Streptomyces lividans 1326
BGC0002158		0.13	NRP, Polyketide	tenuazonic acid	Pyricularia oryzae 70-15
BGC0001441		0.12	Other	belactosin A, belactosin C	Streptomyces sp.
BGC0000410		0.12	NRP	pseudomonine	Pseudomonas fluorescens
BGC0002071		0.12	NRP	virginiafactin A, virginiafactin B, virginiafactin C, virginiafactin D	Pseudomonas sp. QS1027

Gene details

Select a gene to view the details available for it

TFBS Finder

TFBS definitions

Regulator	Description
BldD	Development and antibiotic global regulator
CelR	Cellobiose uptake repressor

anti SMASH

If you have found antiSMASH useful, please cite us .

antiSMASH version 7.1.0

Download About Help Contact

Select genomic region:

Overview 1.1 1.2 1.3 1.4 1.5 1.6 1.7 1.8 1.9 1.10 1.11 1.12 1.13 1.14 1.15 1.16 1.17 1.18 1.19 1.20 1.21 1.22 1.23 1.24 1.25

results - Region 3 - NRPS

Location: 952,397 - 1,000,348 nt. (total: 47,952 nt) Show pHMM detection rules used

Download region SVG Download region GenBank file

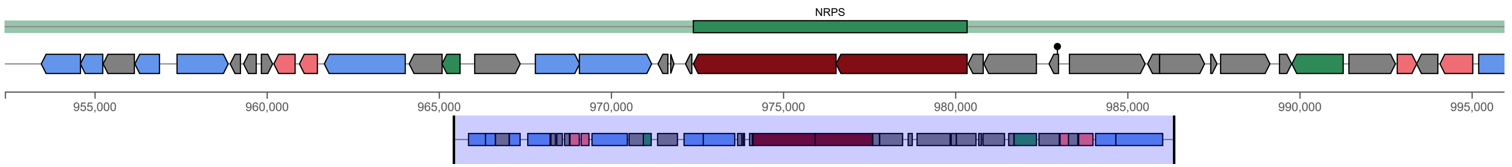

Legend:

core biosynthetic genes | additional biosynthetic genes | transport-related genes | regulatory genes | other genes | resistance | binding site

reset view | zoom to selection

Gene overview | NRPS/PKS domains | MIBiG comparison | ClusterBlast | KnownClusterBlast | SubClusterBlast | TFBS Finder | NRPS/PKS modules | Pfam domains | TIGRFAM domains

Similar gene clusters

Analysis type: Protocluster to Region

Query

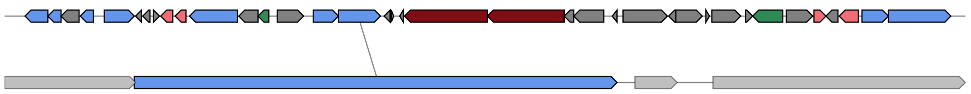

Reference: BGC0002630: 0-4291

Reference	NRPS	Similarity score	Type	Compound(s)	Organism
BGC0002630		0.37	RiPP	thatisin	Lysobacter antibioticus
BGC0001555		0.33	RiPP	colicin V	Escherichia coli chi7122
BGC0001050		0.33	NRP, Polyketide	thalassospiramide A	Tistrella bauzanensis
BGC0000426		0.33	NRP	sevadicin	Paenibacillus larvae
BGC0002071		0.32	NRP	virginiafactin A, virginiafactin B, virginiafactin C, virginiafactin D	Pseudomonas sp. QS1027
BGC0000465		0.32	NRP	xenortide A, xenortide B, xenortide C, xenortide D	Xenorhabdus nematophila ATCC 19061
BGC0000343		0.32	NRP	enterobactin	Pseudomonas sp. J465
BGC0000590		0.30	RiPP	microcin N	Escherichia coli
BGC0000457		0.30	NRP	vicibactin	Rhizobium etli CFN 42
BGC0002058		0.29	Polyketide, NRP	mutanocyclin	Streptococcus mutans B04Sm5

Gene details

Select a gene to view the details available for it

NRPS/PKS products | NRPS/PKS substrates | TFBS Finder

Predicted core structure(s)

For candidate cluster 3, location 952396 - 1000348:

Link to NORINE database query form

anti SMASH

If you have found antiSMASH useful, please cite us.

antiSMASH version 7.1.0 Download About Help Contact

Select genomic region:

Overview 1.1 1.2 1.3 1.4 1.5 1.6 1.7 1.8 1.9 1.10 1.11 1.12 1.13 1.14 1.15 1.16 1.17 1.18 1.19 1.20 1.21 1.22 1.23 1.24 1.25

results - Region 4 - NRPS

Location: 1,189,143 - 1,234,851 nt. (total: 45,709 nt) Show pHMM detection rules used

Download region SVG Download region GenBank file

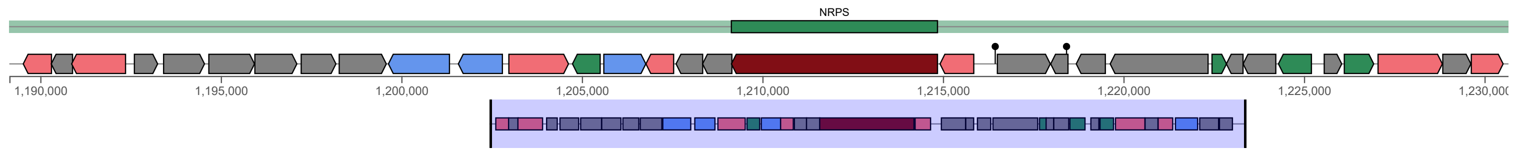

Legend:

core biosynthetic genes | additional biosynthetic genes | transport-related genes | regulatory genes | other genes | resistance | binding site

reset view | zoom to selection

Gene overview | NRPS/PKS domains | MIBiG comparison | ClusterBlast | KnownClusterBlast | SubClusterBlast | TFBS Finder | NRPS/PKS modules | Pfam domains | TIGRFAM domains

Similar gene clusters

Analysis type: Protocluster to Region

Query

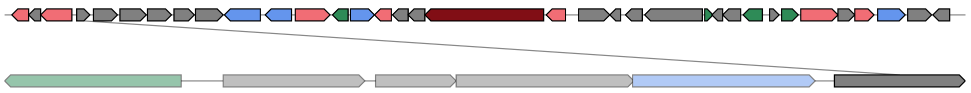

Reference: BGC0001992: 0-4749

Reference	NRPS	Similarity score	Type	Compound(s)	Organism
BGC0001992		0.37	Other	thioguanine	Erwinia amylovora CFBP1430
BGC0000016		0.32	Other	amphotericin B	Streptomyces nodosus
BGC0000457		0.32	NRP	vicibactin	Rhizobium etli CFN 42
BGC0000900		0.31	Other	ferrichrome	Aspergillus oryzae
BGC0001249		0.31	NRP	dimethylcoprogen	Alternaria alternata
BGC0002710		0.28	NRP	metachelin C, metachelin A, metachelin A-CE, metachelin B, dimerumic acid 11-mannoside, dimerumic acid	Metarhizium robertsii ARSEF 23
BGC0002157		0.28	NRP, Alkaloid	(-)-ditryptophenaline	Aspergillus flavus
BGC0000343		0.27	NRP	enterobactin	Pseudomonas sp. J465
BGC0001132		0.26	NRP	xenotetrapeptide	Xenorhabdus nematophila ATCC 19061
BGC0002058		0.26	Polyketide, NRP	mutanocyclin	Streptococcus mutans B04Sm5

Gene details

Select a gene to view the details available for it

NRPS/PKS products | NRPS/PKS substrates | TFBS Finder

Predicted core structure(s)

For candidate cluster 4, location 1189142 - 1234851:

Link to NORINE database query form

If you have found antiSMASH useful, please cite us .

results - Region 5 - NRPS

Location: 1,582,015 - 1,629,963 nt. (total: 47,949 nt) Show pHMM detection rules used

Download region SVG Download region GenBank file

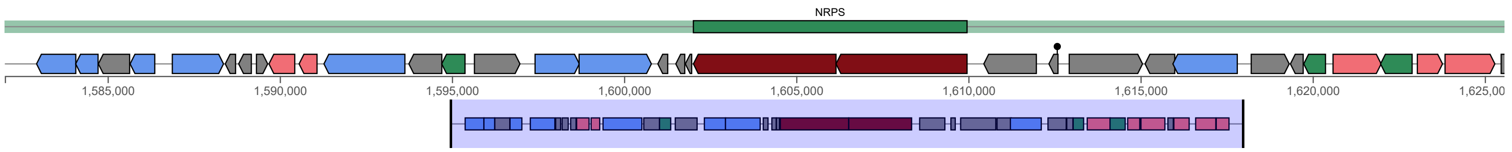

Legend:

core biosynthetic genes | additional biosynthetic genes | transport-related genes | regulatory genes | other genes | resistance | binding site

reset view | zoom to selection

Gene overview | NRPS/PKS domains | MIBiG comparison | ClusterBlast | KnownClusterBlast | SubClusterBlast | TFBS Finder | NRPS/PKS modules | Pfam domains | TIGRFAM domains

Similar gene clusters

Analysis type: Protocluster to Region

Query

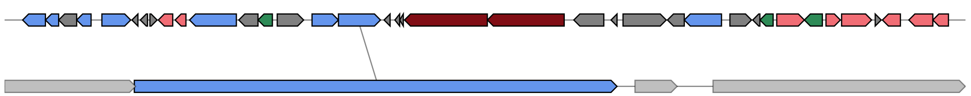

Reference: BGC0002630: 0-4291

Reference	NRPS	Similarity score	Type	Compound(s)	Organism
BGC0002630		0.37	RiPP	thatisin	Lysobacter antibioticus
BGC0001555		0.33	RiPP	colicin V	Escherichia coli chi7122
BGC0000465		0.32	NRP	xenortide A, xenortide B, xenortide C, xenortide D	Xenorhabdus nematophila ATCC 19061
BGC0000343		0.32	NRP	enterobactin	Pseudomonas sp. J465
BGC0002404		0.32	Other	falcarindiol	Solanum lycopersicum
BGC0001050		0.31	NRP, Polyketide	thalassospiramide A	Tistrella bauzanensis
BGC0000590		0.30	RiPP	microcin N	Escherichia coli
BGC0002071		0.30	NRP	virginiafactin A, virginiafactin B, virginiafactin C, virginiafactin D	Pseudomonas sp. QS1027
BGC0001925		0.30	Alkaloid	altemicidin, SB-203207, SB-203208	Streptomyces sp.
BGC0002494		0.28	NRP	vibriobactin	Vibrio cholerae

Gene details

Select a gene to view the details available for it

NRPS/PKS products | NRPS/PKS substrates | TFBS Finder

Predicted core structure(s)

For candidate cluster 5, location 1582014 - 1629963:

Link to NORINE database query form

anti SMASH

If you have found antiSMASH useful, please cite us .

results - Region 6 - NRPS

Location: 1,927,877 - 2,004,607 nt. (total: 76,731 nt) Show pHMM detection rules used

Download region SVG Download region GenBank file

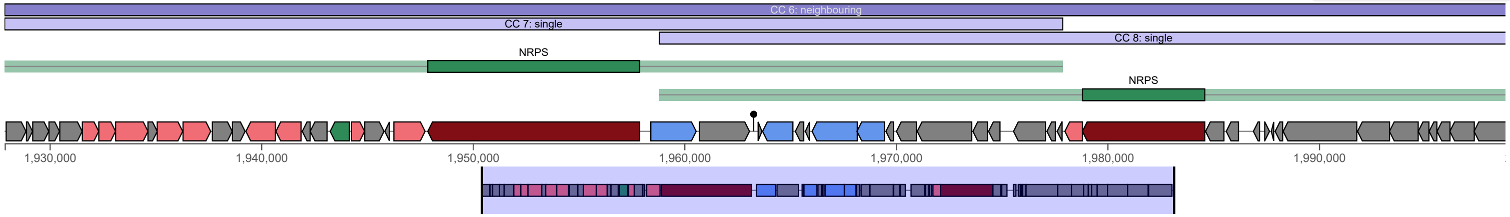

Legend:

core biosynthetic genes | additional biosynthetic genes | transport-related genes | regulatory genes | other genes | resistance | binding site

reset view | zoom to selection

Gene overview | NRPS/PKS domains | MIBiG comparison | ClusterBlast | KnownClusterBlast | SubClusterBlast | TFBS Finder | NRPS/PKS modules | Pfam domains | TIGRFAM domains

Similar gene clusters

Analysis type: Protocluster to Region

Query

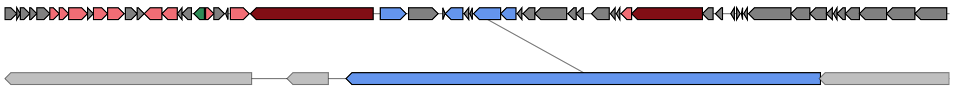

Reference: BGC0002630: 0-4291

Reference	NRPS	NRPS	Similarity score	Type	Compound(s)	Organism
BGC0002630			0.72	RiPP	thatisin	Lysobacter antibioticus
BGC0002005			0.69	RiPP	RaxX	Xanthomonas oryzae pv. oryzae
BGC0001555			0.64	RiPP	colicin V	Escherichia coli chi7122
BGC0000590			0.59	RiPP	microcin N	Escherichia coli
BGC0001132			0.52	NRP	xenotetrapeptide	Xenorhabdus nematophila ATCC 19061
BGC0002437			0.52	NRP	thermoactinoamide A	Thermoactinomyces sp. AS95
BGC0001825			0.51	NRP	xenematide	Xenorhabdus nematophila AN6/1
BGC0001833			0.51	NRP	icosalide A, icosalide B	Burkholderia gladioli
BGC0000588			0.50	RiPP	microcin L	Escherichia coli
BGC0001128			0.50	NRP	gamexpeptide C	Photorhabdus laumondii subsp. laumondii TTO1

Gene details

Select a gene to view the details available for it

NRPS/PKS products | NRPS/PKS substrates | TFBS Finder

Predicted core structure(s)

For candidate cluster 6, location 1927876 - 2004607:

For candidate cluster 7, location 1927876 - 1977893:

For candidate cluster 8, location 1958820 - 2004607:

Link to NORINE database query form

If you have found antiSMASH useful, please cite us.

results - Region 7 - NRPS

Location: 2,088,360 - 2,138,376 nt. (total: 50,017 nt) Show pHMM detection rules used

Download region SVG Download region GenBank file

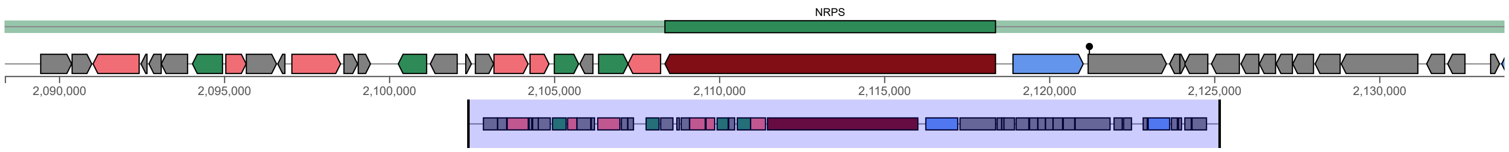

Legend:

core biosynthetic genes | additional biosynthetic genes | transport-related genes | regulatory genes | other genes | resistance | binding site

reset view | zoom to selection

Gene overview | NRPS/PKS domains | MIBiG comparison | ClusterBlast | KnownClusterBlast | SubClusterBlast | TFBS Finder | NRPS/PKS modules | Pfam domains | TIGRFAM domains

Similar gene clusters

Analysis type: Protocluster to Region

Query

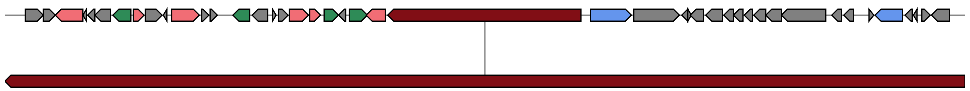

Reference: BGC0002135: 0-11508

Reference	NRPS	Similarity score	Type	Compound(s)	Organism
BGC0002135		0.32	NRP	bovienimide A	Xenorhabdus bovienii SS-2004
BGC0002518		0.32	NRP	syringafactin A, syringafactin C	Pseudomonas sp. SZ57
BGC0000457		0.32	NRP	vicibactin	Rhizobium etli CFN 42
BGC0001833		0.30	NRP	icosalide A, icosalide B	Burkholderia gladioli
BGC0002437		0.28	NRP	thermoactinoamide A	Thermoactinomyces sp. AS95
BGC0001128		0.28	NRP	gamexpeptide C	Photorhabdus laumondii subsp. laumondii TTO1
BGC0001135		0.28	NRP	bicornutin A1, bicornutin A2	Xenorhabdus budapestensis
BGC0002286		0.27	NRP	ririwpeptide A, ririwpeptide B, ririwpeptide C	Photorhabdus laumondii subsp. laumondii TTO1
BGC0002424		0.27	NRP	saccharochelin A, saccharochelin B, saccharochelin C, saccharochelin D, saccharochelin E, saccharochelin F, saccharochelin G, saccharochelin H	Saccharothrix sp.
BGC0001844		0.27	NRP	holrhizin	Paraburkholderia rhizoxinica HKI 454

Gene details

Select a gene to view the details available for it

NRPS/PKS products | NRPS/PKS substrates | TFBS Finder

Predicted core structure(s)

For candidate cluster 9, location 2088359 - 2138376:

Link to NORINE database query form

anti SMASH
If you have found antiSMASH useful, please cite us .

results - Region 8 - NRPS

Location: 3,051,774 - 3,100,284 nt. (total: 48,511 nt) Show pHMM detection rules used

Download region SVG Download region GenBank file

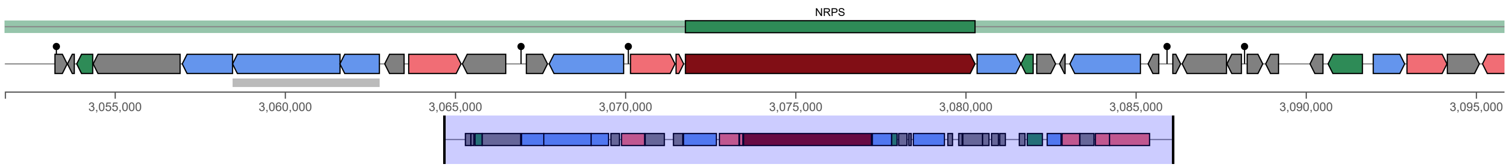

Legend:

core biosynthetic genes | additional biosynthetic genes | transport-related genes | regulatory genes | other genes | resistance | binding site

reset view | zoom to selection

Gene overview | NRPS/PKS domains | MIBiG comparison | ClusterBlast | KnownClusterBlast | SubClusterBlast | TFBS Finder | NRPS/PKS modules | Pfam domains | TIGRFAM domains

Similar gene clusters

Analysis type: Protocluster to Region

Query

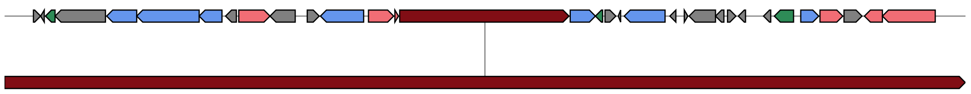

Reference: BGC0002075: 0-3894

Reference	NRPS	Similarity score	Type	Compound(s)	Organism
BGC0002075		0.32	NRP, Alkaloid	pyreudione A, pyreudione B, pyreudione C, pyreudione D, pyreudione E	Pseudomonas fluorescens
BGC0000343		0.32	NRP	enterobactin	Pseudomonas sp. J465
BGC0002359		0.31	NRP	dudomycin A	Streptomyces albus subsp. chlorinus
BGC0002689		0.28	NRP	2,3-dihydroxybenzoylserine	Stenotrophomonas maltophilia K279a
BGC0000392		0.28	NRP	mirubactin	Actinosynnema mirum DSM 43827
BGC0002414		0.27	NRP	trichrysobactin, cyclic trichrysobactin, chrysobactin, dichrysobactin	Dickeya chrysanthemi
BGC0000349		0.25	NRP	erythrochelin	Saccharopolyspora erythraea NRRL 2338
BGC0000597		0.25	RiPP	SWA-2138	Streptomyces sp. e14
BGC0002415		0.25	NRP	vanchrobactin, trivanchrobactin, divanchrobactin	Vibrio campbellii
BGC0000426		0.25	NRP	sevadicin	Paenibacillus larvae

Gene details

Select a gene to view the details available for it

NRPS/PKS products | NRPS/PKS substrates | TFBS Finder

Predicted core structure(s)

For candidate cluster 10, location 3051773 - 3100284:

Link to NORINE database query form

If you have found antiSMASH useful, please cite us.

antiSMASH version 7.1.0 Download About Help Contact

Select genomic region:

Overview 1.1 1.2 1.3 1.4 1.5 1.6 1.7 1.8 1.9 1.10 1.11 1.12 1.13 1.14 1.15 1.16 1.17 1.18 1.19 1.20 1.21 1.22 1.23 1.24 1.25

results - Region 9 - RRE-containing

Location: 3,116,538 - 3,136,816 nt. (total: 20,279 nt) Show pHMM detection rules used

Download region SVG Download region GenBank file

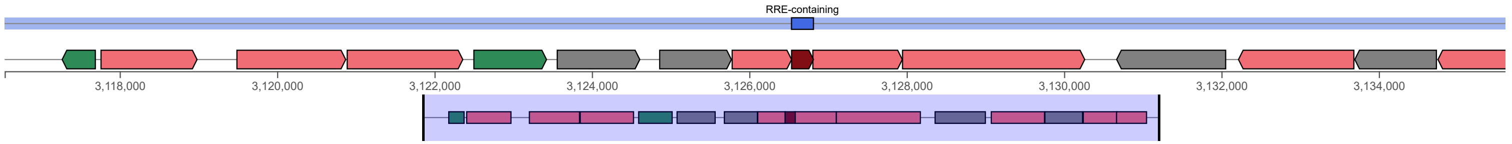

Legend:

core biosynthetic genes | additional biosynthetic genes | transport-related genes | regulatory genes | other genes | resistance | binding site

reset view | zoom to selection

Gene overview | MIBiG comparison | ClusterBlast | KnownClusterBlast | SubClusterBlast | TFBS Finder | Pfam domains | TIGRFAM domains

Similar gene clusters

Analysis type: Protocluster to Region

Query

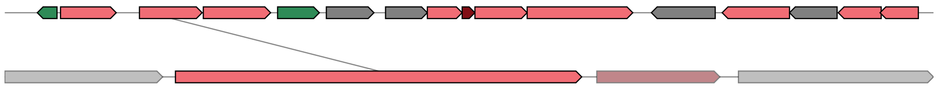

Reference: BGC0000859: 258-3305

Reference	RRE-containing	Similarity score	Type	Compound(s)	Organism
BGC0000859		0.26	Other	ectoine	Methylomicrobium alcaliphilum
BGC0000857		0.26	Other	ectoine	Methylophaga alcalica
BGC0000855		0.26	Other	ectoine	Methylomicrobium kenyense
BGC0000852		0.25	Other	ectoine	Sporosarcina pasteurii
BGC0000858		0.25	Other	ectoine	Methylobacter marinus
BGC0000856		0.25	Other	ectoine	Methylophaga thalassica
BGC0000860		0.25	Other	ectoine	Methylarcula marina
BGC0000919		0.23	Other	phaseolotoxin	Pseudomonas syringae
BGC0000854		0.23	Other	ectoine	Methylomicrobium alcaliphilum 20Z
BGC0002052		0.23	Other (Ectoine)	ectoine	Streptomyces sp.

Gene details

Select a gene to view the details available for it

RREFinder | TFBS Finder

RRE predictions

RRE-containing protocluster (3116537...3136816)
ctg1_2838

If you have found antiSMASH useful, please cite us .

antiSMASH version 7.1.0

Download About Help Contact

Select genomic region:

Overview 1.1 1.2 1.3 1.4 1.5 1.6 1.7 1.8 1.9 1.10 1.11 1.12 1.13 1.14 1.15 1.16 1.17 1.18 1.19 1.20 1.21 1.22 1.23 1.24 1.25

results - Region 10 - RRE-containing

Location: 4,089,325 - 4,109,603 nt. (total: 20,279 nt) Show pHMM detection rules used

Download region SVG Download region GenBank file

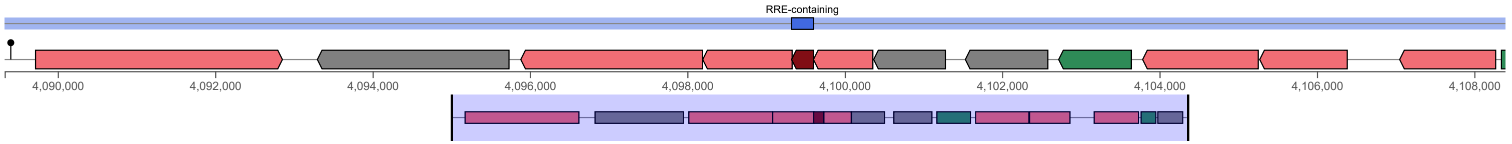

Legend:

core biosynthetic genes | additional biosynthetic genes | transport-related genes | regulatory genes | other genes | resistance | binding site

reset view | zoom to selection

Gene overview | MIBiG comparison | ClusterBlast | KnownClusterBlast | SubClusterBlast | TFBS Finder | Pfam domains | TIGRFAM domains

Similar gene clusters

Analysis type: Protocluster to Region

Query

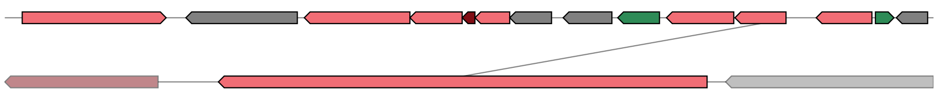

Reference: BGC0000852: 415-2854

Reference	RRE-containing	Similarity score	Type	Compound(s)	Organism
BGC0000852		0.26	Other	ectoine	Sporosarcina pasteurii
BGC0000855		0.25	Other	ectoine	Methylomicrobium kenyense
BGC0000857		0.25	Other	ectoine	Methylophaga alcalica
BGC0000858		0.25	Other	ectoine	Methylobacter marinus
BGC0000859		0.25	Other	ectoine	Methylomicrobium alcaliphilum
BGC0000856		0.25	Other	ectoine	Methylophaga thalassica
BGC0000860		0.25	Other	ectoine	Methylarcula marina
BGC0000919		0.24	Other	phaseolotoxin	Pseudomonas syringae
BGC0002052		0.22	Other (Ectoine)	ectoine	Streptomyces sp.
BGC0000853		0.22	Other	ectoine	Streptomyces anulatus

Gene details

Select a gene to view the details available for it

RREFinder | TFBS Finder

RRE predictions

RRE-containing protocluster (4089324...4109603)
ctg1_3736

If you have found antiSMASH useful, please cite us .

results - Region 11 - NRPS

Location: 4,121,110 - 4,169,620 nt. (total: 48,511 nt) Show pHMM detection rules used

Download region SVG Download region GenBank file

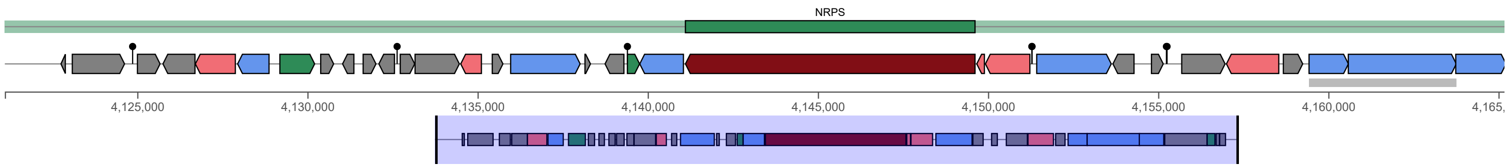

Legend:

core biosynthetic genes | additional biosynthetic genes | transport-related genes | regulatory genes | other genes | resistance | binding site

reset view | zoom to selection

Gene overview | NRPS/PKS domains | MIBiG comparison | ClusterBlast | KnownClusterBlast | SubClusterBlast | TFBS Finder | NRPS/PKS modules | Pfam domains | TIGRFAM domains

Similar gene clusters

Analysis type: Protocluster to Region

Query

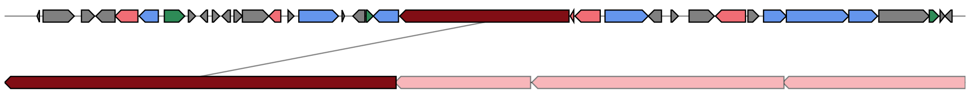

Reference: BGC0000343: 1-6208

Reference	NRPS	Similarity score	Type	Compound(s)	Organism
BGC0000343		0.32	NRP	enterobactin	Pseudomonas sp. J465
BGC0002075		0.32	NRP, Alkaloid	pyreudione A, pyreudione B, pyreudione C, pyreudione D, pyreudione E	Pseudomonas fluorescens
BGC0002359		0.31	NRP	dudomycin A	Streptomyces albus subsp. chlorinus
BGC0000392		0.28	NRP	mirubactin	Actinosynnema mirum DSM 43827
BGC0002689		0.27	NRP	2,3-dihydroxybenzoylserine	Stenotrophomonas maltophilia K279a
BGC0002414		0.26	NRP	trichrysobactin, cyclic trichrysobactin, chrysobactin, dichrysobactin	Dickeya chrysanthemi
BGC0000349		0.25	NRP	erythrochelin	Saccharopolyspora erythraea NRRL 2338
BGC0000597		0.25	RiPP	SWA-2138	Streptomyces sp. e14
BGC0002415		0.25	NRP	vanchrobactin, trivanchrobactin, divanchrobactin	Vibrio campbellii
BGC0000426		0.25	NRP	sevadicin	Paenibacillus larvae

Gene details

Select a gene to view the details available for it

NRPS/PKS products | NRPS/PKS substrates | TFBS Finder

Predicted core structure(s)

For candidate cluster 13, location 4121109 - 4169620:

Link to NORINE database query form

If you have found antiSMASH useful, please cite us.

results - Region 12 - NRPS

Location: 4,200,327 - 4,246,125 nt. (total: 45,799 nt) Show pHMM detection rules used

Download region SVG Download region GenBank file

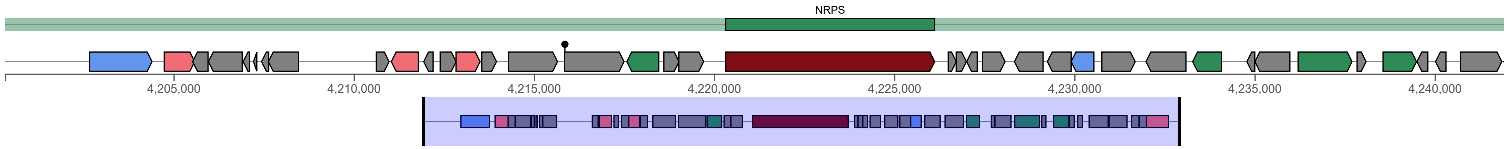

Legend:

core biosynthetic genes | additional biosynthetic genes | transport-related genes | regulatory genes | other genes | resistance | binding site

reset view | zoom to selection

Gene overview | NRPS/PKS domains | MIBiG comparison | ClusterBlast | KnownClusterBlast | SubClusterBlast | TFBS Finder | NRPS/PKS modules | Pfam domains | TIGRFAM domains

Similar gene clusters

Analysis type: Protocluster to Region

Query

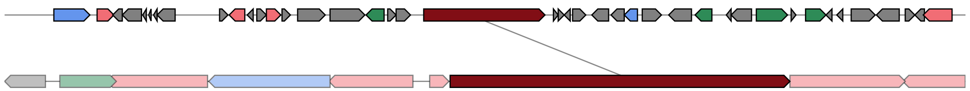

Reference: BGC0000457: 0-11274

Reference	NRPS	Similarity score	Type	Compound(s)	Organism
BGC0000457		0.32	NRP	vicibactin	Rhizobium etli CFN 42
BGC0001132		0.28	NRP	xenotetrapeptide	Xenorhabdus nematophila ATCC 19061
BGC0002058		0.27	Polyketide, NRP	mutanocyclin	Streptococcus mutans B04Sm5
BGC0002287		0.27	NRP	mutanocyclin, leuvalin, tyrvalin	Streptococcus mutans
BGC0001833		0.27	NRP	icosalide A, icosalide B	Burkholderia gladioli
BGC0002135		0.27	NRP	bovienimide A	Xenorhabdus bovienii SS-2004
BGC0001844		0.27	NRP	holrhizin	Paraburkholderia rhizoxinica HKI 454
BGC0001135		0.27	NRP	bicornutin A1, bicornutin A2	Xenorhabdus budapestensis
BGC0000292		0.27	NRP	acetylaranotin	Aspergillus terreus NIH2624
BGC0001128		0.27	NRP	gamexpeptide C	Photorhabdus laumondii subsp. laumondii TTO1

Gene details

Select a gene to view the details available for it

NRPS/PKS products | NRPS/PKS substrates | TFBS Finder

Predicted core structure(s)

For candidate cluster 14, location 4200326 - 4246125:

Link to NORINE database query form

If you have found antiSMASH useful, please cite us.

antiSMASH version 7.1.0

Download About Help Contact

Select genomic region:

Overview 1.1 1.2 1.3 1.4 1.5 1.6 1.7 1.8 1.9 1.10 1.11 1.12 1.13 1.14 1.15 1.16 1.17 1.18 1.19 1.20 1.21 1.22 1.23 1.24 1.25

results - Region 13 - opine-like-metallophore

Location: 4,783,197 - 4,805,294 nt. (total: 22,098 nt) Show pHMM detection rules used

Download region SVG Download region GenBank file

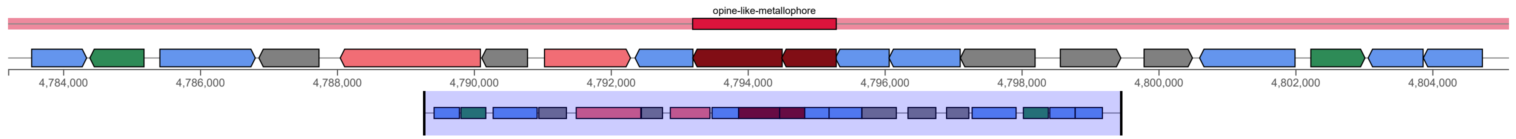

Legend:

core biosynthetic genes | additional biosynthetic genes | transport-related genes | regulatory genes | other genes | resistance | binding site

reset view | zoom to selection

Gene details

Select a gene to view the details available for it

Gene overview | MIBiG comparison | ClusterBlast | KnownClusterBlast | SubClusterBlast | TFBS Finder | Pfam domains | TIGRFAM domains

Similar gene clusters

Analysis type: Protocluster to Region

Query

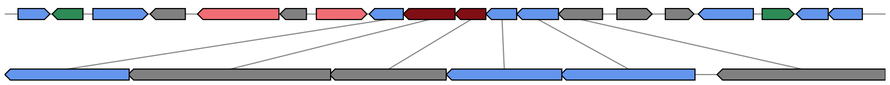

Reference: BGC0002490: 0-5965

Reference	opine-like-metallophore	Similarity score	Type	Compound(s)	Organism
BGC0002490		0.47	Other	yersinopine	Yersinia pestis CO92
BGC0002489		0.27	Other	pseudopaline	Pseudomonas aeruginosa PAO1
BGC0001498		0.16	Other	aerobactin	Xenorhabdus szentirmaii DSM 16338
BGC0002073		0.16	NRP	dehydroxynocardamine	Corynebacterium propinquum
BGC0002325		0.16	RiPP	mycetohabin-16	Mycetohabitans rhizoxinica HKI 454
BGC0002144		0.16	RiPP	phazolicin	Rhizobium sp. Pop5
BGC0000519		0.16	RiPP	labyrinthopeptin A2, labyrinthopeptin A1, labyrinthopeptin A3	Actinomadura namibiensis
BGC0000506		0.16	RiPP	entianin	Bacillus subtilis subsp. spizizenii
BGC0000544		0.16	RiPP	planosporicin	Planomonospora alba
BGC0002635		0.16	RiPP	daspyromycin A, daspyromycin B	Actinokineospora diospyrosa

TFBS Finder

TFBS definitions

Regulator	Description
ZuR	Zinc-responsive repressor
BldD	Development and antibiotic global regulator

anti SMASH
If you have found antiSMASH useful, please cite us .

results - Region 14 - RiPP-like

Location: 5,029,023 - 5,041,548 nt. (total: 12,526 nt) Show pHMM detection rules used

Download region SVG Download region GenBank file

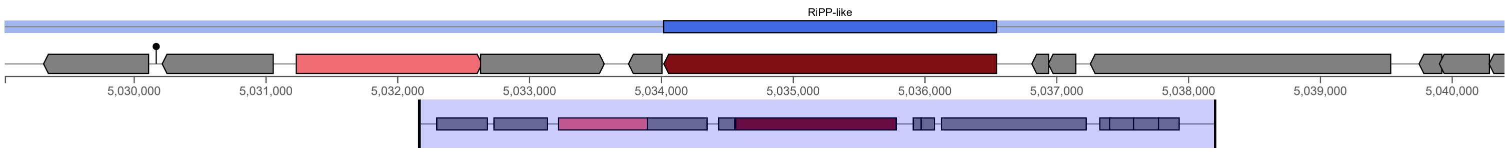

Legend:

core biosynthetic genes | additional biosynthetic genes | transport-related genes | regulatory genes | other genes | resistance | binding site

reset view | zoom to selection

Gene details

Select a gene to view the details available for it

Gene overview | MIBiG comparison | ClusterBlast | KnownClusterBlast | SubClusterBlast | TFBS Finder | Pfam domains | TIGRFAM domains

Similar gene clusters

Analysis type: Protocluster to Region

Query

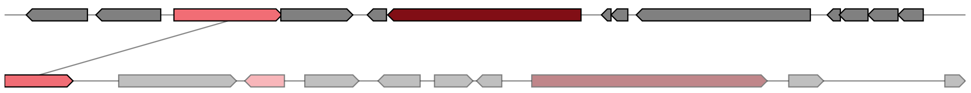

Reference: BGC0000057: 0-26552

Reference	RiPP-like	Similarity score	Type	Compound(s)	Organism
BGC0000057		0.08	Polyketide	F9775A, F9775B, orsellinic acid	Aspergillus nidulans FGSC A4
BGC0000340		0.03	NRP	echoside A, echoside B, echoside C, echoside D, echoside E	Streptomyces sp. LZ35

TFBS Finder

TFBS definitions

Regulator	Description
ZuR	Zinc-responsive repressor
NrtR	NAD synthesis repressor

If you have found antiSMASH useful, please cite us.

results - Region 15 - RiPP-like

Location: 5,622,538 - 5,634,301 nt. (total: 11,764 nt) Show pHMM detection rules used

Download region SVG Download region GenBank file

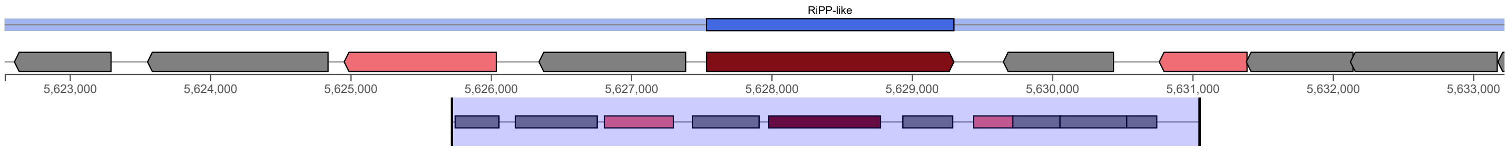

Legend:

core biosynthetic genes | additional biosynthetic genes | transport-related genes | regulatory genes | other genes | resistance | binding site

reset view | zoom to selection

Gene details

Select a gene to view the details available for it

Gene overview | MIBiG comparison | ClusterBlast | KnownClusterBlast | SubClusterBlast | Pfam domains | TIGRFAM domains

Similar gene clusters

Analysis type: Protocluster to Region

Query

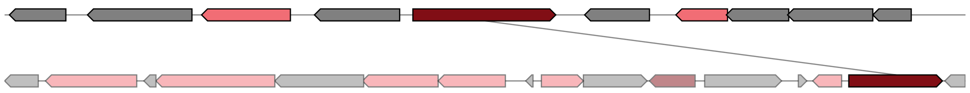

Reference: BGC0001285: 8-22479

Reference	RiPP-like	Similarity score	Type	Compound(s)	Organism
BGC0001285		0.10	Other (Fatty acid)	pseudopyronine A, pseudopyronine B	Pseudomonas putida
BGC0002439		0.09	Other	diastaphenazine, izumiphenazine C	Streptomyces diastaticus
BGC0002362		0.08	Polyketide	loseolamycin A1, loseolamycin A2	Micromonospora endolithica
BGC0001302		0.06	Other	lomofungin	Streptomyces lomondensis
BGC0000698		0.05	Saccharide	hygromycin A	Streptomyces hygroscopicus
BGC0002012		0.04	Polyketide	julichrome Q3-3, julichrome Q3-5	Streptomyces afghaniensis 772
BGC0000700		0.04	Saccharide	istamycin	Streptomyces tenjimariensis
BGC0001283		0.04	Polyketide	arsono-polyketide	Streptomyces lividans 1326
BGC0000957		0.04	NRP, Polyketide	mycotrienin I	Streptomyces sp. XZQH13
BGC0000001		0.03	Polyketide	abyssomicin C, atrop-abyssomicin C	Verrucosispora maris AB-18-032

If you have found antiSMASH useful, please cite us .

results - Region 16 - HR-T2PKS

Location: 6,072,461 - 6,116,415 nt. (total: 43,955 nt) Show pHMM detection rules used

Download region SVG Download region GenBank file

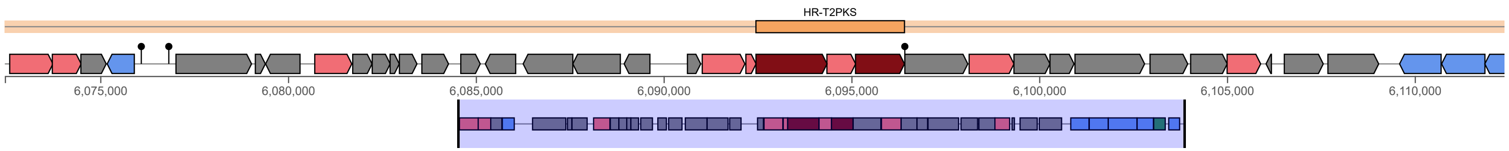

Legend:

core biosynthetic genes | additional biosynthetic genes | transport-related genes | regulatory genes | other genes | resistance | binding site

reset view | zoom to selection

Gene overview | NRPS/PKS domains | MIBiG comparison | ClusterBlast | KnownClusterBlast | SubClusterBlast | TFBS Finder | Pfam domains | TIGRFAM domains

Similar gene clusters

Analysis type: Protocluster to Region

Query

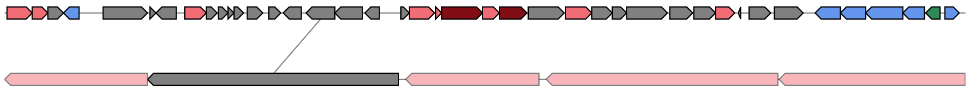

Reference: BGC0000278: 25-5408

Reference	HR-T2PKS	Similarity score	Type	Compound(s)	Organism
BGC0000278		0.27	Polyketide	urdamycin	Streptomyces fradiae
BGC0000882		0.26	Other	mildiomycin	Streptomyces rimofaciens
BGC0000898		0.25	Saccharide	desosamine, pikromycin, neomethymycin, narbomycin	Streptomyces venezuelae
BGC0001238		0.25	Other	biotin	Aspergillus nidulans
BGC0000193		0.24	Polyketide	aclacinomycin	Streptomyces galilaeus
BGC0002043		0.23	RiPP	cittilin A, cittilin B	Myxococcus xanthus DK 1622
BGC0002476		0.23	NRP	enterobactin	Escherichia coli str. K-12 substr. MG1655
BGC0000917		0.22	Other	molybdenum cofactor	Rhodobacter capsulatus
BGC0001239		0.22	Other	biotin	Aspergillus nidulans
BGC0000025		0.22	Polyketide	avermectin	Streptomyces avermitilis

Gene details

Select a gene to view the details available for it

NRPS/PKS substrates | TFBS Finder

NRPS/PKS substrate predictions

anti SMASH

If you have found antiSMASH useful, please cite us .

antiSMASH version 7.1.0

Download About Help Contact

Select genomic region:

Overview 1.1 1.2 1.3 1.4 1.5 1.6 1.7 1.8 1.9 1.10 1.11 1.12 1.13 1.14 1.15 1.16 1.17 1.18 1.19 1.20 1.21 1.22 1.23 1.24 1.25

results - Region 17 - NRP-metallophore,NRPS

Location: 6,156,546 - 6,220,599 nt. (total: 64,054 nt) Show pHMM detection rules used

Download region SVG Download region GenBank file

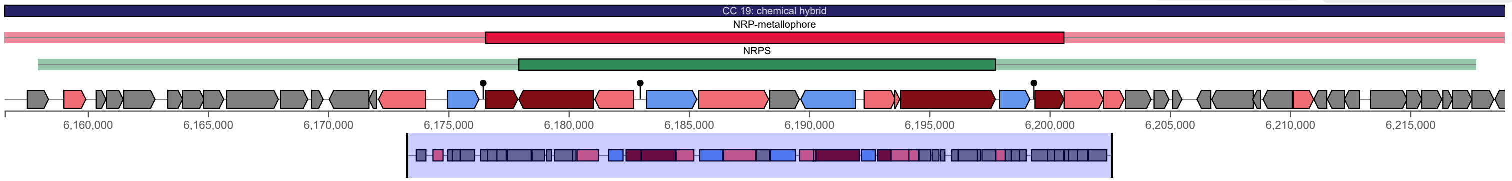

Legend:

core biosynthetic genes | additional biosynthetic genes | transport-related genes | regulatory genes | other genes | resistance | binding site

reset view | zoom to selection

Gene overview | NRPS/PKS domains | MIBiG comparison | ClusterBlast | KnownClusterBlast | SubClusterBlast | TFBS Finder | NRPS/PKS modules | Pfam domains | TIGRFAM domains

Similar gene clusters

Analysis type: Protocluster to Region

Query

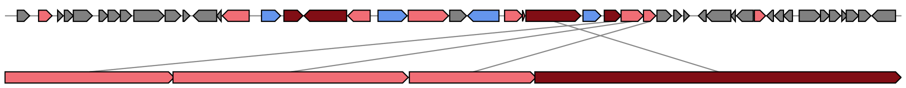

Reference: BGC0000343: 1-6208

Reference	NRP-metallophore	NRPS	Similarity score	Type	Compound(s)	Organism
BGC0000343			0.91	NRP	enterobactin	Pseudomonas sp. J465
BGC0002075			0.65	NRP, Alkaloid	pyreudione A, pyreudione B, pyreudione C, pyreudione D, pyreudione E	Pseudomonas fluorescens
BGC0001185			0.62	NRP	bacillibactin	Bacillus velezensis FZB42
BGC0002528			0.58	NRP	aminochelin, azotochelin, protochelin	Azotobacter vinelandii CA
BGC0002476			0.58	NRP	enterobactin	Escherichia coli str. K-12 substr. MG1655
BGC0002359			0.57	NRP	dudomycin A	Streptomyces albus subsp. chlorinus
BGC0001615			0.53	NRP	hexose-palythine-serine, hexose-shinorine	Heteroscytonema crispum UCFS10
BGC0002495			0.52	NRP	photobactin	Photorhabdus luminescens
BGC0002474			0.52	NRP	agrobactin	Agrobacterium tumefaciens
BGC0001133			0.52	NRP	taxlllaid A	Xenorhabdus bovienii SS-2004

Gene details

Select a gene to view the details available for it

NRPS/PKS products | NRPS/PKS substrates | TFBS Finder

Predicted core structure(s)

For candidate cluster 19, location 6156545 - 6220599:

Link to NORINE database query form

If you have found antiSMASH useful, please cite us .

antiSMASH version 7.1.0

Download About Help Contact

Select genomic region:

Overview 1.1 1.2 1.3 1.4 1.5 1.6 1.7 1.8 1.9 1.10 1.11 1.12 1.13 1.14 1.15 1.16 1.17 1.18 1.19 1.20 1.21 1.22 1.23 1.24 1.25

results - Region 18 - NRPS

Location: 6,297,834 - 6,343,542 nt. (total: 45,709 nt) Show pHMM detection rules used

Download region SVG Download region GenBank file

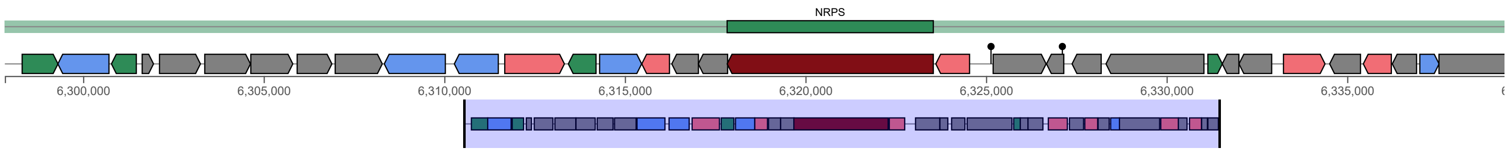

Legend:

core biosynthetic genes additional biosynthetic genes transport-related genes regulatory genes other genes resistance binding site

reset view zoom to selection

Gene overview NRPS/PKS domains MIBiG comparison ClusterBlast KnownClusterBlast SubClusterBlast TFBS Finder NRPS/PKS modules Pfam domains TIGRFAM domains

Similar gene clusters

Analysis type: Protocluster to Region

Query

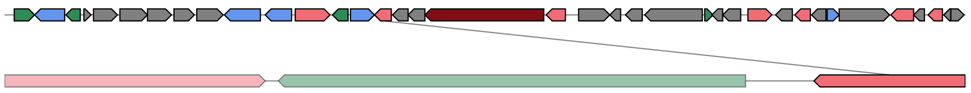

Reference: BGC0000016: 226-5727

Reference	NRPS	Similarity score	Type	Compound(s)	Organism
BGC0000016		0.33	Other	amphotericin B	Streptomyces nodosus
BGC0000457		0.32	NRP	vicibactin	Rhizobium etli CFN 42
BGC0001992		0.31	Other	thioguanine	Erwinia amylovora CFBP1430
BGC0000900		0.31	Other	ferrichrome	Aspergillus oryzae
BGC0001249		0.31	NRP	dimethylcoprogen	Alternaria alternata
BGC0001585		0.28	Alkaloid	melinacidin IV	Escovopsis weberi
BGC0002157		0.28	NRP, Alkaloid	(-)-ditryptophenaline	Aspergillus flavus
BGC0000343		0.27	NRP	enterobactin	Pseudomonas sp. J465
BGC0001261		0.27	NRP	AM-toxin	Alternaria alternata
BGC0001132		0.26	NRP	xenotetrapeptide	Xenorhabdus nematophila ATCC 19061

Gene details

Select a gene to view the details available for it

NRPS/PKS products NRPS/PKS substrates TFBS Finder

Predicted core structure(s)

For candidate cluster 20, location 6297833 - 6343542:

Link to NORINE database query form

anti SMASH

If you have found antiSMASH useful, please cite us .

results - Region 19 - hserlactone

Location: 6,461,624 - 6,482,298 nt. (total: 20,675 nt) Show pHMM detection rules used

Download region SVG Download region GenBank file

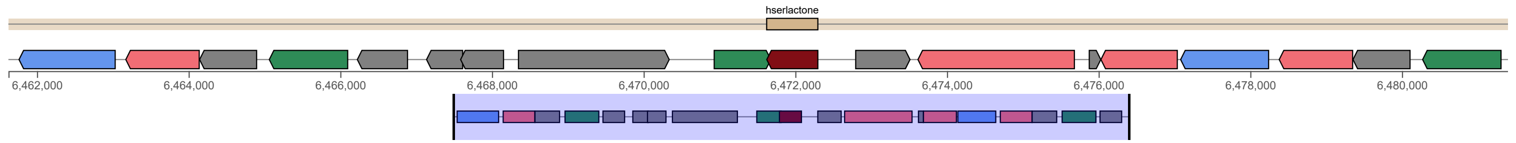

Legend:

core biosynthetic genes | additional biosynthetic genes | transport-related genes | regulatory genes | other genes | resistance | binding site

reset view | zoom to selection

Gene overview | MIBiG comparison | ClusterBlast | KnownClusterBlast | SubClusterBlast | Pfam domains | TIGRFAM domains

Similar gene clusters

Analysis type: Protocluster to Region

Query

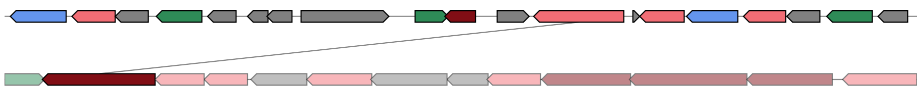

Reference: BGC0001510: 425-14535

Reference	hserlactone	Similarity score	Type	Compound(s)	Organism
BGC0001510		0.12	Other	anisomycin	Streptomyces hygrospinosus
BGC0002716		0.12	NRP	gamexpeptide A, gamexpeptide B, gamexpeptide E, luminmide B, luminmide D, luminmide E, luminmide F, luminmide G	Photorhabdus laumondii subsp. laumondii
BGC0001636		0.11	NRP	KK-1	Curvularia clavata
BGC0001047		0.10	NRP, Polyketide	syringolin A	Pseudomonas syringae
BGC0000804		0.09	Saccharide	acarviostatin I03, acarviostatin II03, acarviostatin III03, acarviostatin IV03	Streptomyces coelicoflavus ZG0656
BGC0000691		0.09	Saccharide	acarbose	Actinoplanes sp. SE50/110
BGC0002282		0.08	Other	formycin A	Streptomyces kaniharaensis
BGC0002040		0.08	Other	coformycin, formycin A	Streptomyces kaniharaensis
BGC0002657		0.08	Polyketide, NRP	deoxyhangtaimycin	Streptomyces spectabilis
BGC0001705		0.06	NRP, Polyketide	nodularin	Nostoc sp. CENA543

Gene details

Select a gene to view the details available for it

anti SMASH
If you have found antiSMASH useful, please cite us .

antiSMASH version 7.1.0 Download About Help Contact

Select genomic region:

Overview 1.1 1.2 1.3 1.4 1.5 1.6 1.7 1.8 1.9 1.10 1.11 1.12 1.13 1.14 1.15 1.16 1.17 1.18 1.19 1.20 1.21 1.22 1.23 1.24 1.25

results - Region 20 - NRPS,T1PKS

Location: 7,439,405 - 7,494,026 nt. (total: 54,622 nt) Show pHMM detection rules used

Download region SVG Download region GenBank file

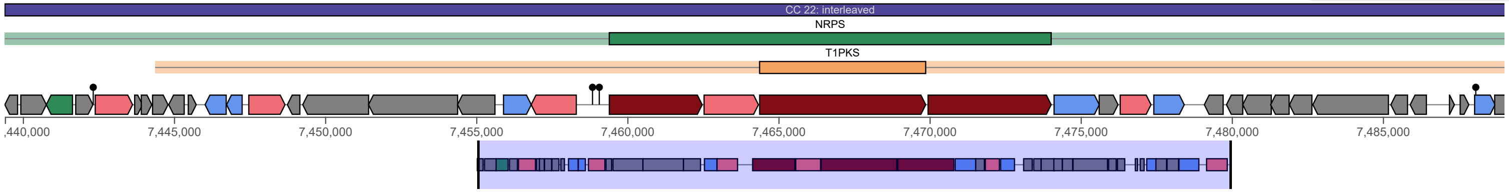

Legend:

core biosynthetic genes | additional biosynthetic genes | transport-related genes | regulatory genes | other genes | resistance | binding site

reset view | zoom to selection

Gene details

Select a gene to view the details available for it

Gene overview | NRPS/PKS domains | MIBiG comparison | ClusterBlast | KnownClusterBlast | SubClusterBlast | TFBS Finder | NRPS/PKS modules | Pfam domains | TIGRFAM domains

Similar gene clusters

Analysis type: Protocluster to Region

Query

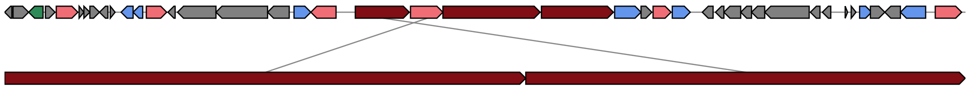

Reference: BGC0001133: 0-25888

Reference	NRPS	T1PKS	Similarity score	Type	Compound(s)	Organism
BGC0001133			0.70	NRP	taxlllaid A	Xenorhabdus bovienii SS-2004
BGC0000979			0.69	NRP, Polyketide	cylindrospermopsin	Aphanizomenon sp. 10E9
BGC0000980			0.69	NRP, Polyketide	cylindrospermopsin	Aphanizomenon sp. 22D11
BGC0000375			0.68	NRP	indigoidine	Streptomyces chromofuscus
BGC0000465			0.67	NRP	xenortide A, xenortide B, xenortide C, xenortide D	Xenorhabdus nematophila ATCC 19061
BGC0002071			0.67	NRP	virginiafactin A, virginiafactin B, virginiafactin C, virginiafactin D	Pseudomonas sp. QS1027
BGC0002528			0.64	NRP	aminochelin, azotochelin, protochelin	Azotobacter vinelandii CA
BGC0000343			0.64	NRP	enterobactin	Pseudomonas sp. J465
BGC0002075			0.64	NRP, Alkaloid	pyreudione A, pyreudione B, pyreudione C, pyreudione D, pyreudione E	Pseudomonas fluorescens
BGC0001876			0.64	NRP	thalassospiramide A	Thalassospira sp. CNJ-328

NRPS/PKS products | NRPS/PKS substrates | TFBS Finder

Predicted core structure(s)

For candidate cluster 22, location 7439404 - 7494026:

Link to NORINE database query form

anti SMASH

If you have found antiSMASH useful, please cite us .

results - Region 21 - NRPS

Location: 7,692,464 - 7,736,402 nt. (total: 43,939 nt) Show pHMM detection rules used

Download region SVG Download region GenBank file

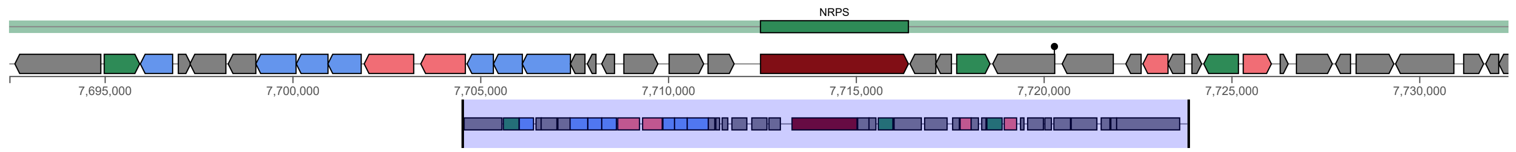

Legend:

core biosynthetic genes | additional biosynthetic genes | transport-related genes | regulatory genes | other genes | resistance | binding site

reset view | zoom to selection

Gene overview | NRPS/PKS domains | MIBiG comparison | ClusterBlast | KnownClusterBlast | SubClusterBlast | TFBS Finder | NRPS/PKS modules | Pfam domains | TIGRFAM domains

Similar gene clusters

Analysis type: Protocluster to Region

Query

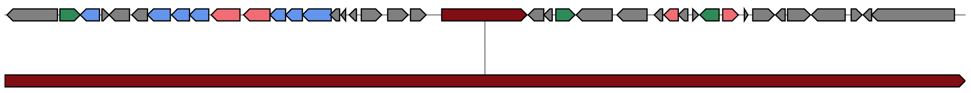

Reference: BGC0002075: 0-3894

Reference	NRPS	Similarity score	Type	Compound(s)	Organism
BGC0002075		0.32	NRP, Alkaloid	pyreudione A, pyreudione B, pyreudione C, pyreudione D, pyreudione E	Pseudomonas fluorescens
BGC0002476		0.30	NRP	enterobactin	Escherichia coli str. K-12 substr. MG1655
BGC0002494		0.29	NRP	vibriobactin	Vibrio cholerae
BGC0002415		0.27	NRP	vanchrobactin, trivanchrobactin, divanchrobactin	Vibrio campbellii
BGC0000343		0.27	NRP	enterobactin	Pseudomonas sp. J465
BGC0002685		0.27	NRP	enterobactin	Rothia mucilaginosa ATCC 25296
BGC0001132		0.26	NRP	xenotetrapeptide	Xenorhabdus nematophila ATCC 19061
BGC0001736		0.26	NRP	pepteridine A, pepteridine B	Photorhabdus laumondii subsp. laumondii TTO1
BGC0001833		0.26	NRP	icosalide A, icosalide B	Burkholderia gladioli
BGC0002689		0.25	NRP	2,3-dihydroxybenzoylserine	Stenotrophomonas maltophilia K279a

Gene details

Select a gene to view the details available for it

NRPS/PKS products | NRPS/PKS substrates | TFBS Finder

Predicted core structure(s)

For candidate cluster 23, location 7692463 - 7736402:

Link to NORINE database query form

If you have found antiSMASH useful, please cite us .

antiSMASH version 7.1.0

Download About Help Contact

Select genomic region:

Overview 1.1 1.2 1.3 1.4 1.5 1.6 1.7 1.8 1.9 1.10 1.11 1.12 1.13 1.14 1.15 1.16 1.17 1.18 1.19 1.20 1.21 1.22 1.23 1.24 1.25

results - Region 22 - NRPS-like

Location: 8,566,404 - 8,609,391 nt. (total: 42,988 nt) Show pHMM detection rules used

Download region SVG Download region GenBank file

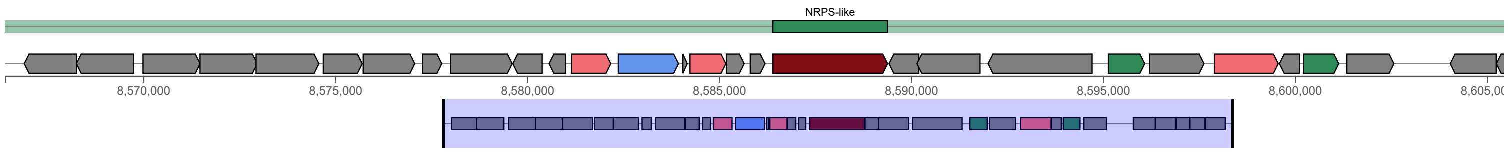

Legend:

core biosynthetic genes | additional biosynthetic genes | transport-related genes | regulatory genes | other genes | resistance | binding site

reset view | zoom to selection

Gene details

Select a gene to view the details available for it

Gene overview | NRPS/PKS domains | MIBiG comparison | ClusterBlast | KnownClusterBlast | SubClusterBlast | TFBS Finder | NRPS/PKS modules | Pfam domains | TIGRFAM domains

Similar gene clusters

Analysis type: Protocluster to Region

Query

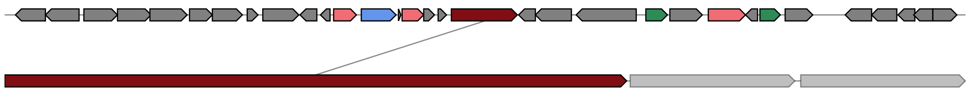

Reference: BGC0001168: 0-4582

Reference	NRPS-like	Similarity score	Type	Compound(s)	Organism
BGC0001168		0.32	NRP	livipeptin	Streptomyces lividans 1326
BGC0001599		0.28	NRP	fragin	Burkholderia cenocepacia H111
BGC0002285		0.27	RiPP	aborycin	Streptomyces sp. ZS0098
BGC0002582		0.27	NRP	guanipiperazine A, guanipiperazine B	Streptomyces chrestomyceticus
BGC0000894		0.24	Other	citrinin	Monascus aurantiacus
BGC0000343		0.23	NRP	enterobactin	Pseudomonas sp. J465
BGC0002278		0.23	Alkaloid, NRP	brasiliamide I, brasiliamide A, brasiliamide B, 2,2-dibenzylpiperazine, brasiliamide D	Penicillium brasilianum
BGC0000375		0.22	NRP	indigoidine	Streptomyces chromofuscus
BGC0002295		0.22	NRP, Saccharide	minimycin	Streptomyces hygroscopicus
BGC0002518		0.19	NRP	syringafactin A, syringafactin C	Pseudomonas sp. SZ57

NRPS/PKS products | NRPS/PKS substrates | TFBS Finder

Predicted core structure(s)

For candidate cluster 24, location 8566403 - 8609391:

Link to NORINE database query form

If you have found antiSMASH useful, please cite us.

antiSMASH version 7.1.0

Download About Help Contact

Select genomic region:

Overview 1.1 1.2 1.3 1.4 1.5 1.6 1.7 1.8 1.9 1.10 1.11 1.12 1.13 1.14 1.15 1.16 1.17 1.18 1.19 1.20 1.21 1.22 1.23 1.24 1.25

results - Region 23 - NRP-metallophore,NRPS

Location: 8,762,914 - 8,865,751 nt. (total: 102,838 nt) Show pHMM detection rules used

Download region SVG Download region GenBank file

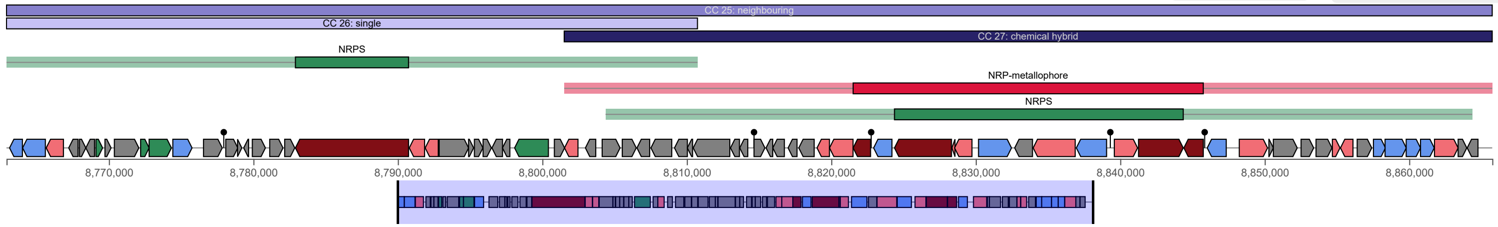

Legend:

core biosynthetic genes · additional biosynthetic genes · transport-related genes · regulatory genes · other genes · resistance · binding site

reset view · zoom to selection

Gene details

Select a gene to view the details available for it

Gene overview · NRPS/PKS domains · MIBiG comparison · ClusterBlast · KnownClusterBlast · SubClusterBlast · TFBS Finder · NRPS/PKS modules · Pfam domains · TIGRFAM domains

Similar gene clusters

Analysis type: Protocluster to Region

Query

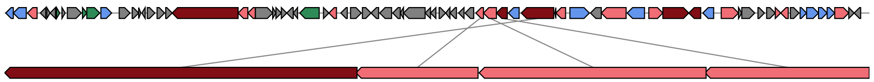

Reference: BGC0000343: 1-6208

Reference	NRPS	NRP-metallophore	NRPS	Similarity score	Type	Compound(s)	Organism
BGC0000343				1.23	NRP	enterobactin	Pseudomonas sp. J465
BGC0002075				0.98	NRP, Alkaloid	pyreudione A, pyreudione B, pyreudione C, pyreudione D, pyreudione E	Pseudomonas fluorescens
BGC0001185				0.91	NRP	bacillibactin	Bacillus velezensis FZB42
BGC0002528				0.84	NRP	aminochelin, azotochelin, protochelin	Azotobacter vinelandii CA
BGC0000401				0.82	NRP	paenibactin	Paenibacillus elgii B69
BGC0001132				0.82	NRP	xenotetrapeptide	Xenorhabdus nematophila ATCC 19061
BGC0001833				0.79	NRP	icosalide A, icosalide B	Burkholderia gladioli
BGC0001128				0.77	NRP	gamexpeptide C	Photorhabdus laumondii subsp. laumondii TTO1
BGC0001758				0.76	NRP	rhizomide A, rhizomide B, rhizomide C	Paraburkholderia rhizoxinica HKI 454
BGC0001844				0.75	NRP	holrhizin	Paraburkholderia rhizoxinica HKI 454

NRPS/PKS products · NRPS/PKS substrates · TFBS Finder

Predicted core structure(s)

For candidate cluster 25, location 8762913 - 8865751:

For candidate cluster 26, location 8762913 - 8810743:

For candidate cluster 27, location 8801516 - 8865751:

Link to NORINE database query form

anti SMASH

If you have found antiSMASH useful, please cite us .

antiSMASH version 7.1.0 Download About Help Contact

Select genomic region:

Overview 1.1 1.2 1.3 1.4 1.5 1.6 1.7 1.8 1.9 1.10 1.11 1.12 1.13 1.14 1.15 1.16 1.17 1.18 1.19 1.20 1.21 1.22 1.23 1.24 1.25

results - Region 24 - NRPS

Location: 9,773,201 - 9,817,754 nt. (total: 44,554 nt) Show pHMM detection rules used

Download region SVG Download region GenBank file

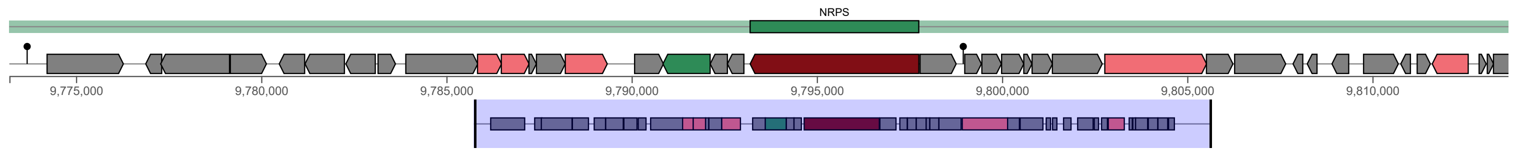

Legend:

core biosynthetic genes | additional biosynthetic genes | transport-related genes | regulatory genes | other genes | resistance | binding site

reset view | zoom to selection

Gene overview | NRPS/PKS domains | MIBiG comparison | ClusterBlast | KnownClusterBlast | SubClusterBlast | TFBS Finder | NRPS/PKS modules | Pfam domains | TIGRFAM domains

Similar gene clusters

Analysis type: Protocluster to Region

Query

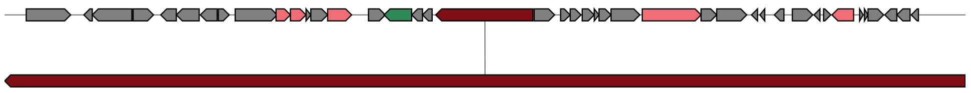

Reference: BGC0001249: 663-5994

Reference	NRPS	Similarity score	Type	Compound(s)	Organism
BGC0001249		0.32	NRP	dimethylcoprogen	Alternaria alternata
BGC0000900		0.31	Other	ferrichrome	Aspergillus oryzae
BGC0000375		0.29	NRP	indigoidine	Streptomyces chromofuscus
BGC0000925		0.28	Other	quinolobactin	Pseudomonas fluorescens
BGC0001132		0.27	NRP	xenotetrapeptide	Xenorhabdus nematophila ATCC 19061
BGC0001128		0.27	NRP	gamexpeptide C	Photorhabdus laumondii subsp. laumondii TTO1
BGC0002286		0.27	NRP	ririwpeptide A, ririwpeptide B, ririwpeptide C	Photorhabdus laumondii subsp. laumondii TTO1
BGC0001135		0.27	NRP	bicornutin A1, bicornutin A2	Xenorhabdus budapestensis
BGC0000917		0.27	Other	molybdenum cofactor	Rhodobacter capsulatus
BGC0002710		0.26	NRP	metachelin C, metachelin A, metachelin A-CE, metachelin B, dimerumic acid 11-mannoside, dimerumic acid	Metarhizium robertsii ARSEF 23

Gene details

Select a gene to view the details available for it

NRPS/PKS products | NRPS/PKS substrates | TFBS Finder

Predicted core structure(s)

For candidate cluster 28, location 9773200 - 9817754:

Link to NORINE database query form

anti SMASH

If you have found antiSMASH useful, please cite us .

antiSMASH version 7.1.0

Download About Help Contact

Select genomic region:

Overview 1.1 1.2 1.3 1.4 1.5 1.6 1.7 1.8 1.9 1.10 1.11 1.12 1.13 1.14 1.15 1.16 1.17 1.18 1.19 1.20 1.21 1.22 1.23 1.24 1.25

results - Region 25 - RiPP-like

Location: 10,043,161 - 10,054,060 nt. (total: 10,900 nt) Show pHMM detection rules used

Download region SVG Download region GenBank file

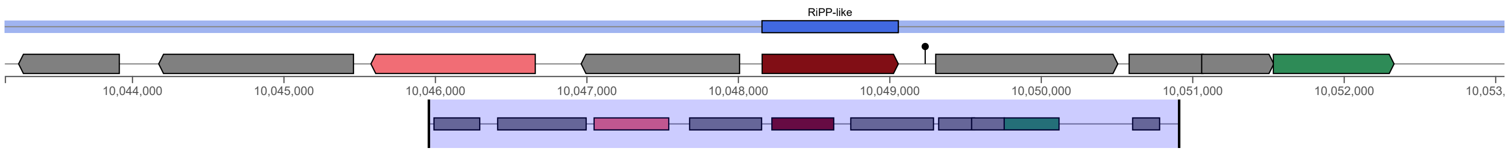

Legend:

core biosynthetic genes | additional biosynthetic genes | transport-related genes | regulatory genes | other genes | resistance | binding site

reset view | zoom to selection

Gene overview | MIBiG comparison | ClusterBlast | KnownClusterBlast | SubClusterBlast | TFBS Finder | Pfam domains | TIGRFAM domains

Similar gene clusters

Analysis type: Protocluster to Region

Query

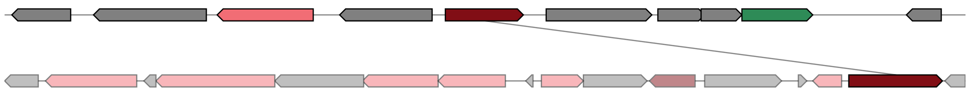

Reference: BGC0001285: 8-22479

Reference	RiPP-like	Similarity score	Type	Compound(s)	Organism
BGC0001285		0.08	Other (Fatty acid)	pseudopyronine A, pseudopyronine B	Pseudomonas putida
BGC0002439		0.06	Other	diastaphenazine, izumiphenazine C	Streptomyces diastaticus
BGC0001302		0.06	Other	lomofungin	Streptomyces lomondensis
BGC0002046		0.05	NRP, Polyketide	lobatamide A	Gynuella sunshinyii YC6258
BGC0002012		0.04	Polyketide	julichrome Q3-3, julichrome Q3-5	Streptomyces afghaniensis 772
BGC0001283		0.04	Polyketide	arsono-polyketide	Streptomyces lividans 1326
BGC0000957		0.04	NRP, Polyketide	mycotrienin I	Streptomyces sp. XZQH13

Gene details

Select a gene to view the details available for it

TFBS Finder

TFBS definitions

Regulator	Description
NrtR	NAD synthesis repressor

If you have found antiSMASH useful, please cite us .
