# Supplementary material for: Pesticide degradation capacity of a novel strain belonging to Serratia sarumanii with its genomic profile
Source: Biodegradation. 2025 Jun 1;36(3):49. doi: 10.1007/s10532-025-10144-2 (PMC12127232; doi:10.1007/s10532-025-10144-2)
Supplement: Supplementary file 2 — Supplementary file2 (PDF 365 KB) [file 10532_2025_10144_MOESM2_ESM.pdf]

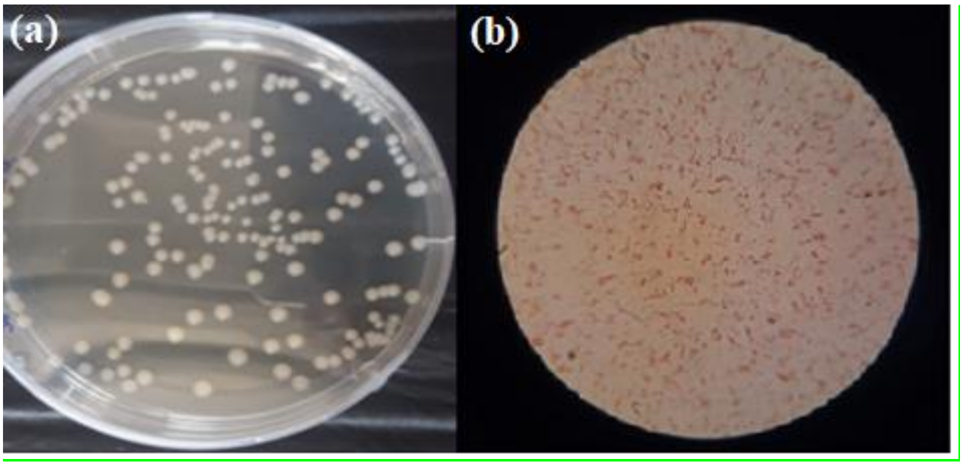

Figure S1. Visual appearance of **a)** pure bacterial colonies and **b)** microscopic image of Gram staining.

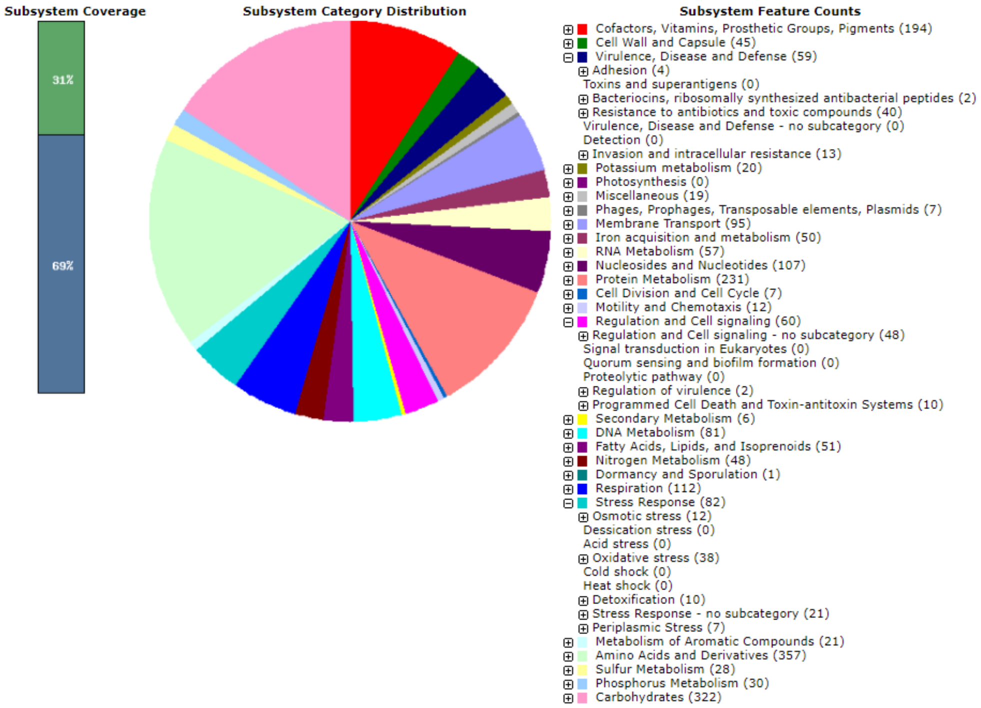

Figure S2. Pie chart of metabolic pathways and their related feature counts.

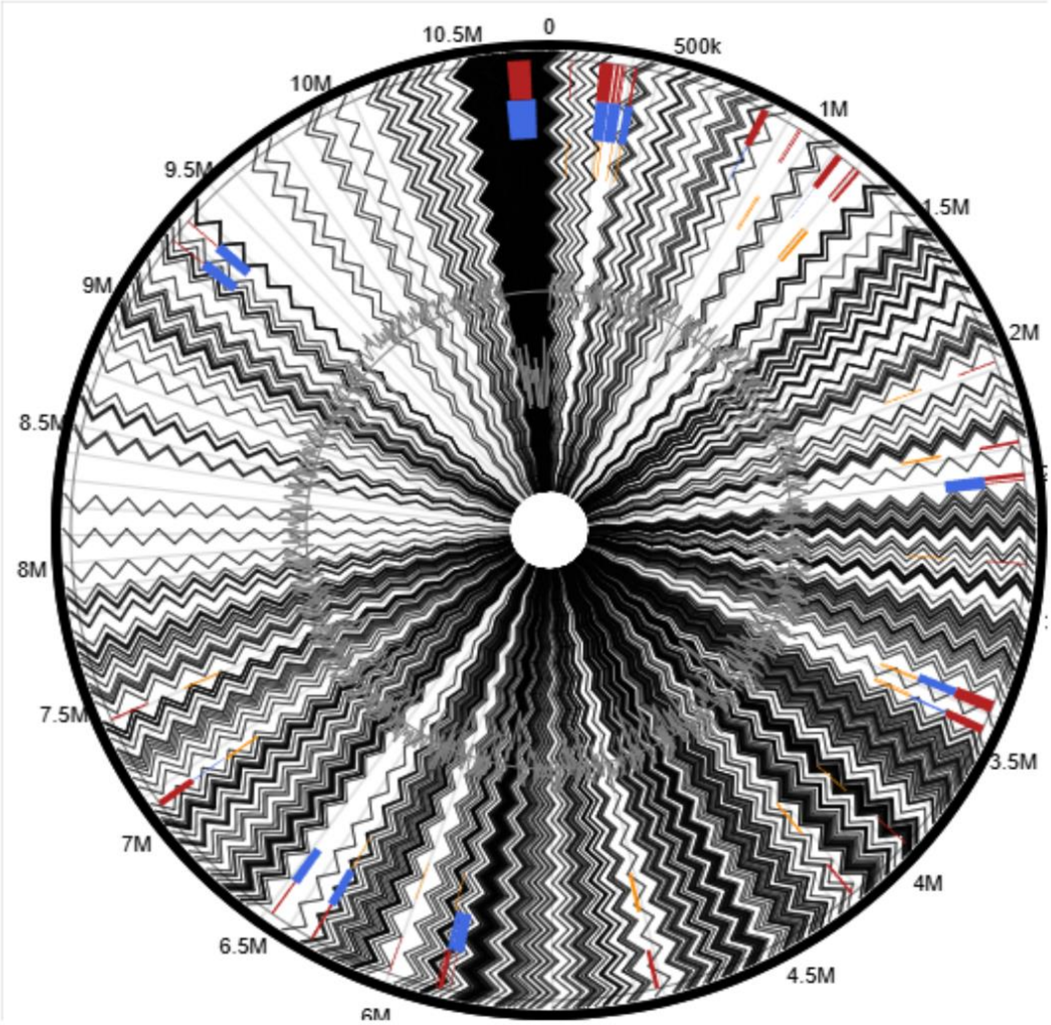

Figure S3. Pathogen IslandViewer 4.0 output of GBS19.
