# Supplementary material for: Pesticide degradation capacity of a novel strain belonging to Serratia sarumanii with its genomic profile
Source: Biodegradation. 2025 Jun 1;36(3):49. doi: 10.1007/s10532-025-10144-2 (PMC12127232; doi:10.1007/s10532-025-10144-2)
Supplement: Supplementary file 3 — Supplementary file3 (PDF 408 KB) [file 10532_2025_10144_MOESM3_ESM.pdf]

Supplementary Tables

Table S1. List of the commercial pesticides tested for pesticide biodegradation according to their active constituents and chemical components.

Pesticides	Active ingredients	Fillers (inert) materials
İnsecticide	Abamectin (18g/l)<br>CAS No: 71751-41-2	Cyclohexanol (%64-68)<br>CAS No: 108-93-0
Fungicide	Penconazole (100 g/l)<br>CAS No: 66246-88-6	Ksilen (%40-60)<br>CAS No: 1330-20-7<br>Cyclohexanone (%20-25)<br>CAS No: 108-94-1
Herbicide	Glyphosate isopropylamine salt (480 g/l)-(%41-42)<br>CAS No: 38641-94-0	POE-(15)-C13/C15 Synthetic amine (%15)<br>CAS No: 61791-26-2<br>Ethoxylated Fatty Amine (%1)<br>CAS No: 26635-92-7

Table S2. The media contents used for testing the pesticide biodegradation ability of bacteria.

**Pesticide groups**	**Tested concentrations**		
	**1. administration**	**2. administration**	**3. administration**
Insecticide Abamectin(18g/l)	25 µl insecticide<br>100 ml Distilled Water<br>1.5 g Agar	50 µl insecticide<br>100 ml Distilled Water<br>1.5 g Agar	75 µl insecticide<br>100 ml Distilled Water<br>1.5 g Agar
Fungicide Penconazole (100 g/l)	25 µl Fungicide<br>100 ml Distilled Water<br>1.5 g Agar	35 µl Fungicide<br>100 ml Distilled Water<br>1.5 g Agar	50 µl Fungicide<br>100 ml Distilled Water<br>1.5 g Agar
Herbicide Glyphosate isopropylamine salt (480 g/l)	300 µl Herbicide<br>100 ml Distilled Water<br>1.5 g Agar	600 µl Herbicide<br>100 ml Distilled Water<br>1.5 g Agar	1000 µl Herbicide<br>100 ml Distilled Water<br>1.5 g Agar

Table S3 The pesticide list linked to their conventional agricultural application that were tested using LC-MS analysis in our assays.

Active Ingredient	Target Organism	Administration dose (100 l water)	Harvesting Time after Administration (days)	g, ml / 5 l water
Penconazole 100 g/l	Vineyard powdery mildew	25 ml	21	1,25
Mancozeb (%60) + Dimethomorph(%9)	Downy mildew	200 g	28	10
Spinetoram	Cluster moth-thrips	20 g	7	1
Metiram (%55) + Pyraclostrobin(%5)	Vineyard powdery mildew	200 g	28	10
Pyrimethanil300 g/l	Gray mold	100 ml	21	5
Alpha- cypermethrin100 g/l	Ligament lock	30 ml	14	1,5
Fenhexamid500 g/l	Gray mold	100 ml	7	5
Indoxacarb150 g/l	Cluster moth	25 ml	3	1,25
Spirodiclofen240 g/l	Red spider	25 ml	14	1,25
Thiophanatemethyl%70	Powdery mildew	100 ml	14	5
Pyrimethanil300 g/l	Gray mold	100 ml	21	5
Lambda-cyhalothrin50 g/l	Cluster moth	20 ml	7	1
Ametoctradin (300 g/l)+Dimethomorph (225g/l)	Downy mildew	100 ml	10	5
EmamectinBenzoate%5	Cluster moth	20 g	14	1
Metrafenone500 g/l	Powdery mildew	20 ml	28/10	1
Azoxystrobin+ Difenoconazole (125 g/l)	Vineyard downy mildew	80 ml	21	4
Cyprodinil (%37.5) + Fludioxonil (%25)	Gray mold	50 g	7	2,5
Lambda-cyhalothrin50 g/l	Cluster moth	20 ml	7	1
Cyprodinil300 g/l	Gray mold	100 ml	7	5
Gamma- cyhalothrin 60 g/l	Cluster moth	15 ml	7	0,75
Boscalid%50	Gray mold	120 g	7	6
Spirotetramat100 g/l	Mealybug	100 ml	14	5

Table S4 a) Pesticide information used in LC-MS analysis.

Pesticide	Pesticide active ingredient
Fungicide	Penconazole, mancozeb, dimethomorp, spinetoram, pyraclostrobin, pyrimethanil, fenhexamid, thiophanate methyl, ametoctradin, metrafenone, azoxystrobin, difenoconazole, Cyprodinil, fludioxonil, fluopyram
Insecticide	alpha cypermethrin, indoxacarb, lambda cyhalothrin, emamectin benzoate, gamma cyhalothrin, tebuconazole, boscalid, spirotetramat, chlorantraniliprole
Acaricide	Spirodiclofen
Nematicide	Fluopyram

b) Information on the LC-MS/MS device used in chromatographic analysis.

Brand/Model: TSQ Quantiva/ Ultimate 3000 (Pump)/ Ultimate 3000 (Autosempler Optiplex 9020/Sogevac_ Vacuum Compressor -Di Ester Oil Vg32-Lv0200/ Parker Balston_ Nitrogen Generator -3848-Nitroflow Lab

Serial No:TQHQ10641/81258594/8123646

Colon: Poroshell 120 EC-C18, (2,7 x 3 µm), 50mm

Mobile A; 5 mM Ammonium formate + 0.1% formic acid dissolved in water

Mobile B; 5 mM Ammonium formate 0.1% formic acid dissolved in methanol

Injection volume:20 µL

Active Ingredient	Ionization Mode	Q_1 (m/z)	Q_3 (m/z)	Coll (V)
Difenoconazole	ESI(+)	406	188,015	18
			251,015	26
Azoxystrobin	ESI(+)	404,12	344,076	27
			372,067	16
Penconazole	ESI(+)	284,12	70,35	20
			159,039	30
Dimethomorph	ESI(+)	388,155	165,118	34
			301,105	23
Spinetoram	ESI(+)	748,7	98,2	44
			142,1	28
Pyraclostrobin	ESI(+)	388,15	149,093	31
			163,107	26
Pyrimethanil	ESI(+)	200,195	168,102	33
			181,14	40
Fenhexamide	ESI(+)	302,12	55,424	38
			97,255	26
Indoxacarb	ESI(+)	528,055	150,06	25
			203,023	38
Spirodiclofen	ESI(+)	411,085	71,376	18
			313,027	14
Thiophanate-Methyl	ESI(+)	343,025	151,135	22
			160,124	32
Ametoctradin	ESI(+)	276,1	149	35

			176	36
Emamectin-benzoate	ESI(+)	872,495	82,279	46
			158,178	37
Metrafenone	ESI(+)	409,3	209	13
			226,9	15
Cyprodinil	ESI(+)	226,205	93,226	37
			118,164	34
Fludioxonil	ESI(-)	247	126,05	32
			151	31
Chlorantraniliprole	ESI(+)	481,88	283,955	13
			450,948	17
Abamectin	ESI(+)	890,4	305,236	27
			567,427	15
Fluopyram	ESI(+)	397,1	144,9	60
			207,9	24
Tebuconazole	ESI(+)	308,2	70,338	24
			125,106	35
Boscalid	ESI(+)	343,04	272,091	34
			307,065	21
Spirotetramat	ESI(+)	374,2	216,032	15
			302,15	16
Lambda cyhalothrin	ESI(+)	467,15	141,375	42,51
			224,929	17,58
Gamma cyhalothrin	ESI(-)	448	421	41
			385	20
Cypermethrin	ESI(+)	433	127	29,89
			191,054	16,88

c) Retention time and flow parameters.

Retention Time (min)	Flow (mL/min)	%A	%B
0	0,5	80	20
0,1	0,5	80	20
0,8	0,5	30	70
3,2	0,5	5	95
4	0,5	5	95
4,2	0,5	80	20
5	0,5	80	20
6	0,5	80	20

Injection mode: Full Loop

PositiveIon (V): 3500.00

NegativeIon (V): 2500.00

Ion Transfer Tube Temp (°C): 300

Vaporizer Temp (°C): 300

Start Time (min): 0

End Time (min): 12 SRM

UseCycle Time: True

Cycle Time (sec): 1.1

UseCalibrated RF Lens: False

Q1 Resolution (FWHM): 0.7

Q3 Resolution (FWHM): 0.7

CID Gas (mTorr): 1.5

Source Fragmentation (V): 0

d) Information on the GC-MS device used in chromatographic analyses.

Brand / Model: TRACH 1300 (Gas Chromatograph)/ ISQ QD/ TRIPLUS RSH

Serial No: 717302476/ISQ1709518/419811

Colon: TR-WAXMS *30 m*0,32 mm*0,25 µm P/N 260X143P S/N 1194341A17 ISQ Series

	Rate(0C/min)	Temp(0C)	Hold time(min)
Initial		80	2.10
1	50	200	0.5

MS Transfer line temp: 280 °C
Ion source Temp: 230 °C
Ionisation mode: EI
GC Parameters:
Max temp: 350°C
Prep-run time out: 50 min
Oven run time: 5 min
Headspace Autosampler
Syringe volume (mL): 2.5
Needle length: 65 mm
Analysis time: 5 min
Sample draw: 1.5 mL
Enrichment delay: 1.0 mm

Incubation
Agitation temp: 95°C
Incubation time: 20 mm
Agitator on: 60 s
Agitator off: 30s
Syringe temp: 100 °C
Filling volume: 1 mL
Post injection syringe flush: 30 s
Filling speed: 5mL/min
Injection speed: 70 mL/min
Injection depth: 35 mm
Penetration speed: 25 mm/s
Post injection delay: 2 s
Solvent Volume: 0.5 mL
Dry time 5s
Anticipitated time: 3 min
Agitator stand temp: 95 °C
Syringe stand-by temp: 100 °C
Needle speed in vial: 20 mm/s

e) Information on pesticides used in physical and chromatographic analyses.

Active Ingredient	Target diseases and pests	Application Dosage (100 l water)	Waiting Time (days)	g, ml / 5 L water
100 g/l Penconazole	Powdery mildew of grape	25 ml	21	1,25
%60 Mancozeb+%9 Dimethomorph	Downy mildew	200 g	28	10
Spinetoram	European Grapevine Moth- Thrips	20 g	7	1
%55 Metiram + %5 Pyraclostrobin	Powdery mildew of grape	200 g	28	10
300 g/l Pyrimethanil	Gray mold	100 ml	21	5
100 g/l Alpha- cypermethrin	Vine weevil	30 ml	14	1,5
500 g/l Fenhexamid	Gray mold	100 ml	7	5
150 g/l Indoxacarb	European grapevine moth	25 ml	3	1,25
240 g/l Spirodiclofen	red spider mite	25 ml	14	1,25
%70 Thiophanate methyl	Powdery mildew	100 ml	14	5
300 g/l Pyrimethanil	Gray mold	100 ml	21	5
50 g/l Lambda-cyhalothrin	European grapevine moth	20 ml	7	1
224.6 g/l *Reynoutria* spp.	Powdery mildew	100 ml	0	5
Ametoctradin 300 g/l+Dimethomorph 225 g/l	Downy mildew	100 ml	10	5
%5 Emamectin Benzoate	European grapevine moth	20 g	14	1
500 g/l Metrafenone	Powdery mildew	20 ml	28	1
Azoxystrobin+ 125 g/l Difenoconazole	Grapevine downy mildew	80 ml	21	4
%37.5 Cyprodinil+%25 Fludioxonil	Gray mold	50 g	7	2,5
50 g/l Lambda-cyhalothrin	European grapevine moth	20 ml	7	1
300 g/l Cyprodinil	Gray mold	100 ml	7	5
200 g/l Chlorantraniliprole	European grapevine moth	15 ml	28	0,75
45g/l Chlorantraniliprole+18 g/l Abamectin	European grapevine moth	70 ml	3	3,5
Gamma- cyhalothrin 60 g/l	European grapevine moth	15 ml	7	0,75
%50 Boscalid	Gray mold	120 g	7	6
100 g/l Spirotetramat	Citrus mealybug	100 ml	14	5

f) LC-MS analysis results.

	Pesticide Active Ingredients	Active g/kg-	Application Dosage g/mL	Concentration (ppb)	1th dilution	2nd dilution	3th dilution	Last concentrati	Standard	GBS19 (+) 72 hours	GBS19 (-)
**1. Group**	**Penconazole**	100	25	25000	50	10	1	50	68±0.01	94±0.01	85±0.01
	**Mancozeb**	600	200	1200000	50	10	1	2400	151±0.03	199±0.01	182±0.02
	**Dimethomorp**	90	200	180000	50	10	1	360	48±0.02	51±0.03	62±0.01
	**Spinetoram**	250	20	50000	50	10	1	100	61±0.01	113±0.02	117±0.03
**2. Group**	**Pyraclostrobin**	50	200	100000	50	10	1	200	634±0.02	2029±0.03	2283±0.01
	**Pyrimethanil**	300	100	300000	50	10	1	600	957±0.02	435±0.01	899±0.02
	**Alpha Cypermethrin**	100	30	30000	50	10	1	60	66±0.01	61±0.03	105±0.02
**3. Group**	**Fenhexamid**	500	100	500000	50	10	1	1000	2829±0.03	1458±0.02	1771±0.01
	**Indoxacarb**	150	25	37500	50	10	1	75	131±0.03	111±0.02	217
	**Spirodiclofen**	240	25	60000	50	10	1	120	420±0.02	98±0.03	306±0.03
**4. Group**	**Thiophanate Methyl**	700	100	700000	50	10	1	1400	1477±0.03	4445±0.03	3435±0.01
	**Pyrimethanil**	300	100	300000	50	10	1	600	1257±0.01	869±0.03	749±0.01
	**Lambda Cyhalothrin**	50	20	10000	50	10	1	20	26±0.02	36±0.03	39±0.02
**5. Group**	**Ametocradin**	300	100	300000	50	10	1	600	545±0.03	570±0.01	588±0.03
	**Dimethomorph**	225	100	225000	50	10	1	450	918±0.01	672±0.03	535±0.02
	**Emamectin Benzoate**	50	20	10000	50	10	1	20	25±0.03	21±0.01	21±0.01
	**Metrafenon**	500	20	100000	50	10	1	200	426±0.01	302±0.03	354±0.01
**6. Group**	**Azoxystrobin**	200	80	160000	50	10	1	320	613±0.01	379±0.03	416±0.01
	**Difenoconazole**	125	80	100000	50	10	1	200	324±0.03	319±0.02	411±0.01
	**Cyprodinil**	375	50	187500	50	10	1	375	380±0.01	514±0.03	437±0.02
	**Fludioxonil**	250	50	125000	50	10	1	250	477±0.03	255±0.03	334
	**Lambda Cyhalothrin**	50	20	10000	50	10	1	20	29±0.03	24±0.02	27±0.01
**7. Group**	**Cyprodinil**	300	100	300000	50	10	1	600	620±0.01	669±0.03	816±0.03
	**Gamma Cyhalothrin**	60	15	9000	50	10	1	18	42±0.02	40±0.03	48±0.01
**8. Group**	**Fluopyram**	200	25	50000	50	10	1	100	236±0.02	267±0.03	248±0.01
	**Tebuconazole**	200	25	50000	50	10	1	100	210±0.03	238±0.01	214±0.01
	**Chlorantraniliprole**	200	15	30000	50	10	1	60	205±0.02	182±0.01	161±0.03
	**Boscalid**	500	120	600000	50	10	1	1200	5503±0.01	4199±0.01	4388±0.03
	**Spirotetramat**	100	100	100000	50	10	1	200	322±0.02	199±0.01	287±0.03

*SD=Standard Error: Calculated using the standard deviation function in Excel.

*µg/kg(ppb)= **PPB**, **Parts Per Billion** In any mixture, 1 part per billion of the total amount of the substance is called 1 ppb. It is the value of the dissolved substance in micrograms in one liter of solution.

Table S5. Biochemical tests on isolated bacterial colony.

Test or substrate	Reaction	Percent
Gram reaction	Gram-negative rod	
Catalase	+	
Oxidase	-	
Motility	+	
Glucose	+	
Mannitol	+	
Sucrose	+	
Methyl red (37 C)	+	20
Methyl red (22 C)	-	7.6
Voges-Proskauer	+	100
Adonitol	+	60
Sorbitol gas	-	0
Arabinose	-	0
Glycerol gas	-	0

Table S6. Degradation pathways associated with genes detected in *S. sarmanuii* GBS19 strain

**Metabolic Pathway**	**KEGG Map Number**
Synthesis and Degradation of Ketone Bodies	00072
Ethylbenzene Degradation	00642
Atrazine Degradation	00791
Styrene Degradation	00643
Steroid Degradation	00984
Caprolactam Degradation	00930
Nitrotoluene Degradation	00633
Fluorobenzoate Degradation	00364
Chloroalkane and Chloroalkene Degradation	00625
Glycosaminoglycan Degradation	00531
Xylene Degradation	00622
Toluene Degradation	00623
Valıne, Leucıne and Isoleucıne Degradation	00280
Naphthalene Degradation	00626
Fatty Acid Degradation	00071
Limonene and Pinene Degradation	00903
Lysine Degradation	00310
Dioxin Degradation	00621
Benzoate Degradation	00362
Aminobenzoate Degradation	00627
Chlorocyclohexane and Chlorobenzene Degradation	00361
Polycyclic Aromatic Hydrocarbon Degradation	00624
Other Glycan Degradation	00511
Geraniol Degradation	00281

Table S7. Xenobiotic degradation pathways associated with genes detected in *S. sarumanii* GBS19 strain

Metabolic Pathway	code
Metabolism of xenobiotics by cytochrome p450	00980
Ethylbenzene Degradation	00642
Drug metabolism - cytochrome p450	00982
Styrene Degradation	00643
Drug metabolism - other enzymes	00983
Steroid degradation	00984
Nitrotoluene Degradation	00633
Fluorobenzoate Degradation	00364
Chloroalkane and Chloroalkene Degradation	00625
Xylene Degradation	00622
Toluene Degradation	00623
Naphthalene Degradation	00626
Dioxin Degradation	00621
Benzoate Degradation	00362
Aminobenzoate Degradation	00627
Chlorocyclohexane and Chlorobenzene Degradation	00361
Polycyclic Aromatic Hydrocarbon Degradation	00624

Table S8. Genes encoded at key points of degradation pathways associated with genes of *S. sarumanii* GBS19 strain

**Metabolic Pathway**	**Gene Expression**	**Gene Symbol**
Ethylbenzene degradation	acetyl-CoA acyltransferase	FadA
Geraniol degradation	acetyl-CoA acyltransferase	FadA
Geraniol degradation	enoyl-CoA hydratase	PaaF
Geraniol degradation	3-hydroxyacyl-CoA dehydrogenase	HADH
Caprolactam degradation	3-hydroxyacyl-CoA dehydrogenase	HADH
Caprolactam degradation	enoyl-CoA hydratase	PaaF
Chloroalkane and chloroalkene degradation	alcohol dehydrogenase	AdhP
Chloroalkane and chloroalkene degradation	Acetaldehyde dehydrogenase	adhE
Glycosaminoglycan degradation	Beta-N-acetylhexosaminidase	NagZ
Xylene degradation	Acetaldehyde dehydrogenase	adhE
Toluene degradation	3-hydroxyacyl-CoA dehydrogenase	HADH
Naphthalene degradation	alcohol dehydrogenase	AdhP
Naphthalene degradation	Acetaldehyde dehydrogenase	adhE
Aminobenzoate degradation	enoyl-CoA hydratase	PaaF
Aminobenzoate degradation	acylphosphatase	AcyP
Aminobenzoate degradation	3-hydroxyacyl-CoA dehydrogenase	HADH
Benzoate degradation	acetyl-CoA acyltransferase	FadA
Benzoate degradation	3-oxoadipyl-CoA thiolase.	PcaF
Benzoate degradation	Acetaldehyde dehydrogenase	adhE
Benzoate degradation	3-hydroxyacyl-CoA dehydrogenase	HADH
Benzoate degradation	enoyl-CoA hydratase	PaaF
Atrazine degradation	Allophanate hydrolase	AtzF
Dioxin degradation	Acetaldehyde dehydrogenase	adhE
